# Silylative Amide to Nitrile Conversion Mediated by Simple Lanthanide–Organoamides: Scope and Mechanism

**DOI:** 10.1002/anie.202513996

**Published:** 2025-09-26

**Authors:** Zhiyu Feng, Qingheng Lai, Yuang Wang, Alessandro Motta, Yosi Kratish, Tobin J. Marks

**Affiliations:** ^1^ Department of Chemistry Northwestern University 2145 Sheridan Rd Evanston Illinois 60208–3113 USA; ^2^ Dipartimento di Scienze Chimiche Università di Roma “La Sapienza” and INSTM UdR Roma Roma I‐00185 Italy

**Keywords:** DFT analysis, Kinetic/Mechanistic evaluation, Nitrile synthesis, Organolanthanide

## Abstract

Efficient, selective, and environmentally benign catalytic nitrile synthesis is attractive for pharmaceuticals, specialty chemicals and materials, and large‐scale industrial applications. In this regard, metal‐catalyzed silylative conversion of primary amides to nitriles is emerging as a promising approach. This contribution reports the utilization of readily available lanthanide‐organic amido precatalysts, Ln[N(SiMe_3_)_2_]_3_, Ln = lanthanide, to selectively convert primary alkyl and aryl/heterocyclic amides having diverse functional groups to nitriles, including pharma building blocks, in high yields using the silane reagents PhSiH_3_ and TMS‐O‐[Si(H)(Me)‐O‐]_n_‐TMS in a solvent‐free process. Kinetic and mechanistic data reveal the role of lanthanide amidates as the catalytically‐active species, while DFT analysis indicates a catalytic pathway unlike that found in transition metal complex‐catalyzed processes. Thus, the lanthanide amidate resting state actively participates in the catalysis, where rate‐determining bound amidate silylation is activated by the metal center and influenced by the bound amidate electronic and steric characteristics. DFT analysis of the catalytic cycle reveals that the relative energies of three intermediate endergonic steps, hence the rate‐determining step, depends on the silane concentration.

## Graphical abstract

Described here is a La^NTMS^ mediated silylative conversion of primary amides to nitriles under neat reaction conditions. The reaction is highly efficient and selective under such solventless conditions, where all materials are commercially available, demonstrating the robustness and potential of lanthanide heteroatom catalysis.

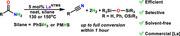

## Introduction

Nitrile functionalities are of great importance in pharmaceuticals,^[^ ^1^, ^2^ ^]^ synthetic building blocks,^[^ ^3^ ^]^ as well as in functional polymeric and magnetic materials,^[^ ^4^, ^5^ ^]^ and many synthetic strategies have been developed to create nitriles. Conventional approaches include the use of toxic cyanides to effect halide or alcohol substitution,^[^ ^6^, ^7^ ^]^ and alkene or alkyne hydrocyanation,^[^ ^8^ ^]^ which typically require harsh conditions, while promising alternative electrochemical and photochemical pathways are currently under active investigation.^[^ ^9^, ^10^ ^]^ Note that the catalytic conversion of primary amides to nitriles provides an attractive functional group transformation since primary amides are abundant synthetic and natural product building blocks,^[^ ^11^ ^]^ and the only formal coproduct is water, which aligns with green chemistry principles.^[^ ^12^ ^]^ Nevertheless, primary amide → nitrile conversions have traditionally been achieved with environmentally harsh acid‐generating dehydrating agents such as POCl_3_ and TiCl_4_,^[^ ^13^, ^14^ ^]^ and more recently with Cu,^[^ ^15^ ^]^ Fe,^[^ ^16^, ^17^, ^18^, ^19^ ^]^ Co,^[^ ^20^ ^]^ Ru,^[^ ^21^ ^]^ Zn,^[^ ^22^, ^23^ ^]^ V,^[^ ^24^ ^]^ and Mn,^[^ ^25^ ^]^ homogeneous catalysts. Many of these processes are ostensibly silylative dehydrations, driven by the thermodynamically leveraged formation of strong Si‐O linkages and H_2_ elimination,^[^ ^26^, ^27^ ^]^ and if sufficiently rapid and selective, are attractive (e.g. Equation 1).^[^ ^28^ ^]^

(1) $$\begin{array}{cc} & 2\mathrm{PhSi}H_{3}+\mathrm{RC}\left(O\right){\mathrm{NH}}_{2}\to \mathrm{RC}\equiv N\\ & +\mathrm{PhSi}{\left(H\right)}_{2}-O-\mathrm{Si}{\left(H\right)}_{2}\mathrm{Ph}+H_{2}\;\Delta H\approx -24\;\mathrm{kcal}\;\mathrm{mo}l^{-1} \end{array}$$

As homogeneous catalysts, lanthanides are distinctive due to their earth‐abundance (as abundant as Cu and Ni), minimal toxicity,^[^ ^29^ ^]^ high electrophilicity, and kinetic lability,^[^ ^30^, ^31^, ^32^ ^]^ large and tunable ionic radii, and generally predictable +3 oxidation states.^[^ ^33^ ^]^ The scope of catalytic transformations is broad, involving both polar and non‐polar substrates, and encompassing olefin, alkyne hydroamination,^[^ ^34^ ^]^ olefin/polyolefin, alkyne hydrophosphination,^[^ ^35^ ^]^ olefin/polyolefin hydrosilylation,^[^ ^36^ ^]^ olefin/polyolefin hydroboration,^[^ ^37^, ^38^ ^]^ olefin/polyolefin, alkyne hydrogenation,^[^ ^39^ ^]^ olefin, alkyne polymerization,^[^ ^40^ ^]^ nylon depolymerization,^[^ ^41^ ^]^ formamide decarbonlylation,^[^ ^42^ ^]^ and C─H borylation.^[^ ^43^ ^]^ Turnover frequencies as high as 10^4^–10^6^ h^−1^ are achieved in high selectivities via non‐classical mechanistic pathways. More importantly, as a well‐established catalyst for over a quarter century,^[^ ^44^ ^]^ homoleptic lanthanide Ln[N(SiMe_3_)_2_]_3_ (Ln^NTMS^) catalysts are known to mediate transformations such as azide addition,^[^ ^45^ ^]^ imidazole synthesis,^[^ ^46^ ^]^ hydroamination and Tishchenko reactions.^[^ ^34^, ^47^, ^48^ ^]^ Particularly relevant to the amide → nitrile challenge are facile and chemoselective Ln^NTMS^‐mediated aldehyde and ketone hydroborations,^[^ ^37^ ^]^ and hydroborylative amide → amine deoxygenations,^[^ ^49^ ^]^ raising the intriguing question of whether Ln^NTMS^ catalysts might be effective in Equation (1), and if so, with what scope and by what mechanistic pathway(s).

**Scheme 1:**
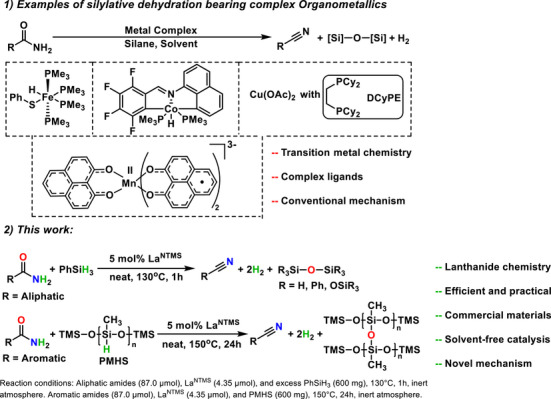
La^NTMS^ catalyst efficiently mediates the conversion of primary amides to nitriles with silanes under solventless conditions.

Here we report that Ln^NTMS^ catalysts efficiently and selectively mediate the silylative conversion of primary amides to nitriles (Scheme 1). It will be seen that this system is compatible with a variety of amides, including pharmaceutical precursors, under solvent‐free conditions, can use inexpensive silanes, and enables substantial conversion rates and functional group tolerance. Moreover, kinetic/mechanistic studies and a DFT analysis implicate a reaction mechanism differing significantly from those of typical homogeneous transition metal catalysts.

## Results and Discussion

Initial NMR‐scale survey reactions were carried out under anhydrous/anaerobic conditions, first monitoring the reaction of the model substrate, hexanamide, with PhSiH_3_ in the presence of a series of Ln complexes to probe metal ionic radius correlations with rates, followed by examination of catalyst ancillary ligand effects, optimization of reaction conditions, and then substrate/functional group and silane screening. The results then feed into experimental kinetic/mechanistic and computational DFT analyses.

### Lanthanide Ion Size and Ancillary Ligand Effects

Lanthanide complex catalytic activity for Equation (1) was initiated with a screen of Ln^NTMS^ catalysts of varying ionic radius under the conditions of Table 1. All reactions proceed with high selectivity, and activity falls with declining ionic radius, La^NTMS ^> Nd^NTMS ^> Eu^NTMS ^> Gd^NTMS ^> Lu^NTMS^. Next investigated were catalyst ancillary ligand effects. Note that Nd[CH(SiMe_3_)_2_]_3_ and Cp*_2_Lu[CH(SiMe_3_)_2_] have significantly lower activity than their Ln^NTMS^ congeners, likely reflecting greater steric constraints in accord also with the superiority of the La^NTMS^ catalyst versus the other Ln^NTMS^ complexes. Note also that La(OTf)_3_ and LaCl_3_ are catalytically inert, consistent with low nucleophilicity and polarity of the La‐ligand bonding, despite the high metal center Lewis acidity.

**Table 1:** Yields versus Lanthanide Ion Size and Ancillary Ligands in Amide to Nitrile Conversion.^a)^

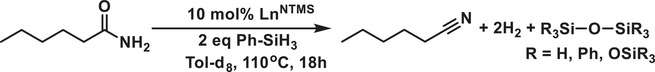			
Entry	Pre‐catalyst	Yield (%)	Ln^3+^ ionic radius (Å)
1	La^NTMS^	84	1.03
2	Nd^NTMS^	81	0.98
3	Eu^NTMS^	64	0.95
4	Gd^NTMS^	63	0.94
5	Lu^NTMS^	52	0.86
6	Nd[CH(SiMe_3_)_2_]_3_	75	0.98
7	Cp*_2_Lu[CH(SiMe_3_)_2_]	13	0.86

^a)^ Reaction conditions: 0.17 mmol hexanamide, 10 mol% pre‐catalyst loading, 2 equiv. PhSiH_3_, 0.5 mL Tol‐d_8_, 110 °C, 18 h, inert atmosphere. Yields assayed by ^1^H NMR with mesitylene internal standard.

### Reaction Condition Optimization

With the optimal choice of La[N(SiMe_3_)_2_]_3_ pre‐catalyst in hand, optimization of reaction conditions was carried out, beginning with 10 mol% La^NTMS^, and varied silane concentrations, solvent, and temperature (Table 2 **, entries 1–4**). It is found that increases in temperature and silane concentration afford higher conversion rates. Solvent effects were next examined, and polar dioxane was found to be compatible with the La^NTMS^ species but to have little effect on conversion rate (Table 2 **, entry 5**). Furthermore, amide to nitrile conversion proceeds well under solvent‐free conditions (Table 2 **, entry 6**), using stoichiometric excess phenylsilane (PhSiH_3_) or the inexpensive silicone industry byproduct, polymethylhydrosiloxane (PMHS) (Table 2 **, entry 9**), with full conversion achieved in 1 h at 5 mol% catalyst loading (Table 2 **, entries 7, 9**). Therefore, solvent‐free conditions were adopted for subsequent substrate screening studies.

**Table 2:** Reaction Condition Optimization for Amide to Nitrile Conversion.

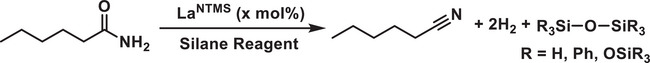					
Entry	Mol% La^NTMS^	Equiv. silane	Solvent	Temp/time	Yield^1)^ (%)
1	10	3 PhSiH_3_	Tol‐d_8_	110 °C 24h	86
2	10	2 PhSiH_3_	Tol‐d_8_	110 °C 18h	84
3	10	2 PhSiH_3_	Tol‐d_8_	100 °C 18h	78
4	10	2 PhSiH_3_	Tol‐d_8_	r.t. 18h	trace
5	10	2 PhSiH_3_	1,4‐dioxane	90 °C 18h	85
6	10	2 PhSiH_3_	Solventless	110 °C 24h	71
7	5	neat PhSiH_3_	Solventless	130 °C 1h	98
8	0.5	neat PhSiH_3_	Solventless	130 °C 18h	41
9	5	neat PMHS	Solventless	150 °C 1h	99

^1)^ Yields determined by ^1^H NMR with mesitylene internal standard. Neat [PhSiH_3_] indicates an approximate molar excess of 60 equiv.

## Substrate Scope

### Alkyl Amides

This study began with a survey of aliphatic primary amides using 5 mol% La^NTMS^ and stoichiometrically excess neat PhSiH_3_ (Table 3). Aliphatic amides are generally compatible with these conditions and uniform conversions independent of alkyl chain length are achieved. The cyanocyclohexylnitrile **3h** synthesis provides a yield of only 62% reflects product decomposition under the reaction conditions. This hypothesis is validated by diminishing of the ^1^H NMR peak integral when neat cyanocyclohexylnitrile is heated under the same conditions. Similarly, adamantylnitrile **3g** synthesis reaches 73% yield within the first hour and then converts to an unknown product. In the case of t‐butylnitrile **3c**, presumed steric encumbrance significantly hinders the conversion rate. A preparative scale synthesis in good yield was achieved for the pharma nitrile derivative **3i** although the rate is sluggish due to poor amide solubility in PhSiH_3_. This limitation was rectified with PHMS as described below.

**Table 3:** Yields of Catalytic Amide to Nitrile Conversion using PhSiH_3_.^a)^

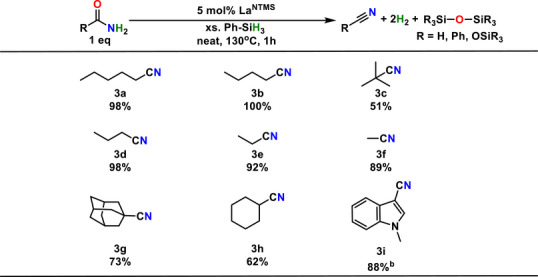

^a)^ Reaction conditions: Amide (87.0 µmol), La^NTMS^ (4.35 µmol), and excess PhSiH_3_ (600 mg), 130 °C, 1 h, inert atmosphere. Yields by ^1^H NMR with mesitylene internal standard.

^b)^ Preparative scale reaction. Yield, 198 mg. Reaction conditions: Amide (250 mg, 1.44 mmol), La^NTMS^ (71.8 µmol), and excess PhSiH_3_ (10 mL). Reaction at 130 °C under inert atmosphere for 30 h. Product purification by column chromatography eluting with 40% ethyl acetate in hexane.

### Aryl/Aromatic Amides

To achieve reasonable yields, aryl amide to nitrile conversion with PhSiH_3_ requires 24–48 h reflecting low substrate solubility, presumably reflecting π‐π stacking and lower basicity/electron‐richness for La^+3^ coordination/activation. However higher boiling, arene‐free (important for ^1^H NMR monitoring of the reaction), and lower cost PMHS delivers higher activities, and a variety of aromatic amides are readily converted to nitriles within 24 h (Table 4). Next, reported rotational barrier values and molecular electrostatic potentials (MESPs) were introduced to qualitatively assess substrate substituent stereo‐electronic effects.^[^ ^50^, ^51^ ^]^ Comparing *para*‐positioned *tert*‐butyl **4k** and methyl **4j** substituents, it appears that steric congestion correlates with lower activity, in accord with greater steric congestion for isopropyl ($\Delta G_{\mathrm{rot}}^{‡}$ = 36.7 kcal mol^−1^) versus methyl ($\Delta G_{\mathrm{rot}}^{‡}$ = 33.7 kcal mol^−1^). Electronic effects also play an apparent role with more electron‐withdrawing naphthyl‐amide **4e** (Δ*V_c_* = 0.2 kcal mol^−1^ for C_6_H_5_) affording a lower yield than benzamide **4a**, and the more electron‐withdrawing F (**4f)** affording lower activity than less electron‐withdrawing Cl (**4g)**.^[^ ^52^ ^]^ Similarly, activity is greater when F is *meta*‐substituted in **4h** versus *para*‐substituted in **4f**. Note also that *meta*‐methoxy‐benzamide substrate **4c** is more active than the *ortho‐*substituted **4d** and *para*‐substituted **4b** isomers. Cl substitution also has an inverse effect in the meta position **4i**, having lower activity than F (**4h)**, likely reflecting dominant steric effects, where Cl ($\Delta G_{\mathrm{rot}}^{‡}$ =  31.9 kcal mol^−1^) is more sterically hindered than F ($\Delta G_{\mathrm{rot}}^{‡}$ = 19.7 kcal mol^−1^), even if F is more electron‐withdrawing.

**Table 4:** Yields of Catalytic Amide to Nitrile Conversion using PMHS.^a)^

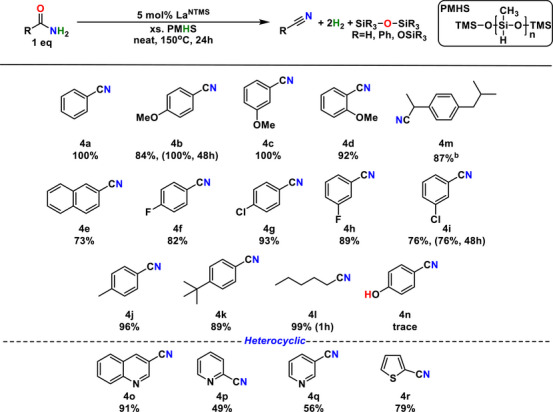

^a)^ Reaction conditions: Amide (87.0 µmol), La^NTMS^ (4.35 µmol), and PMHS (600 mg), 150 °C, 24 h, inert atmosphere. Yields by ^1^H NMR with the mesitylene internal standard.

^b)^ Preparative scale reaction. Isolated yield, 200 mg. Conditions: amide (250 mg, 1.22 mmol), La^NTMS^ (61.0 µmol), and 4 equiv. PMHS (293 mg) in toluene (10 mL total volume). Reaction proceeds at 110 °C under inert atmosphere for 30 h. Product purification by column chromatography (10% ethyl acetate in hexane).

**Scheme 2:**
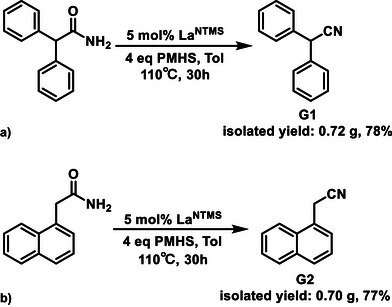
Gram scale syntheses of diphenylacetonitrile and 1‐naphthaleneacetonitrile. a) Gram scale synthesis of diphenylacetonitrile. Conditions: Amide (1 g, 4.73 mmol), La^NTMS^ (237 µmol), and 4 eq. PMHS (1.14 g) in toluene (25 mL total volume). Product purification with column chromatography (10% ethyl acetate in hexane). b) Gram scale synthesis of 1‐naphthaleneacetonitrile. Conditions: Amide (1 g, 5.40 mmol), La^NTMS^ (270 µmol), and 4 eq. PMHS (1.3 g) in toluene (25 mL total volume). Product purification with column chromatography (15% ethyl acetate in hexane).

Note that PMHS readily converts alkyl substrate **4l**, there remain limitations in the functional group tolerance. The hydroxy group, as shown in **4n**, appears to deactivate the La^NTMS^ catalyst via the oxygen binding to lanthanum and the release of HN(SiMe_3_)_2_, considering that the pKa of OH is much lower than the pKa of HN(SiMe_3_)_2_.^[^ ^53^, ^54^ ^]^ Different types of heterocyclic functionalities were examined as well, including piperidine (**4o**), pyridine (**4p** and **4q**), and thiophene (**4r**). These functionalities are tolerated well by the lanthanum catalyst, although the molecules containing pyridine functionality (**4p** and **4q**) react more slowly compared to others, presumably because the nitrogen lone pair electrons can coordinate to the La center, competitively blocking the active catalytic site.^[^ ^55^ ^]^ In contrast, the bulky piperidine group of **4o** prevents such coordination, although it also has a nitrogen lone pair. Although the sulfur electrons should also coordinate to the La center, the conversion rate is not significantly impacted, as seen for **4r**. Since sulfur is a softer Lewis base compared to nitrogen, its coordination to the La^+3^ hard acid is reasonably less favored.^[^ ^56^ ^]^ Lastly, Ibuprofen nitrile derivative **4m** was prepared and isolated on a preparative scale using 4 equiv. of PMHS in toluene solution to demonstrate the scalability of this catalytic process. A lower stoichiometry of PMHS was used in some large‐scale syntheses because at this scale, PMHS forms a gel‐like higher‐Mw polymer, which is less reactive.^[^ ^57^ ^]^ Considering this challenge, adding a toluene solvent is more practical for large‐scale synthesis with PMHS, and the combination of PMHS and toluene is more economical than neat phenylsilane, with negligible reactivity being compromised. In addition to **4m** (Table 4), gram‐scale syntheses were demonstrated for two other aromatic substrates to verify scalability (Scheme 2, G1 and G2). Since both substrates are soluble in toluene at elevated temperatures, the same reaction conditions for **4m** were utilized, and the reaction conditions for Indole derivative **3i** (Table 3) are available for substrates with polar functional groups that are not readily soluble in toluene.

## Kinetics and Mechanism

### Initial‐Rate Kinetics and Activation Parameters

**Figure 1:**
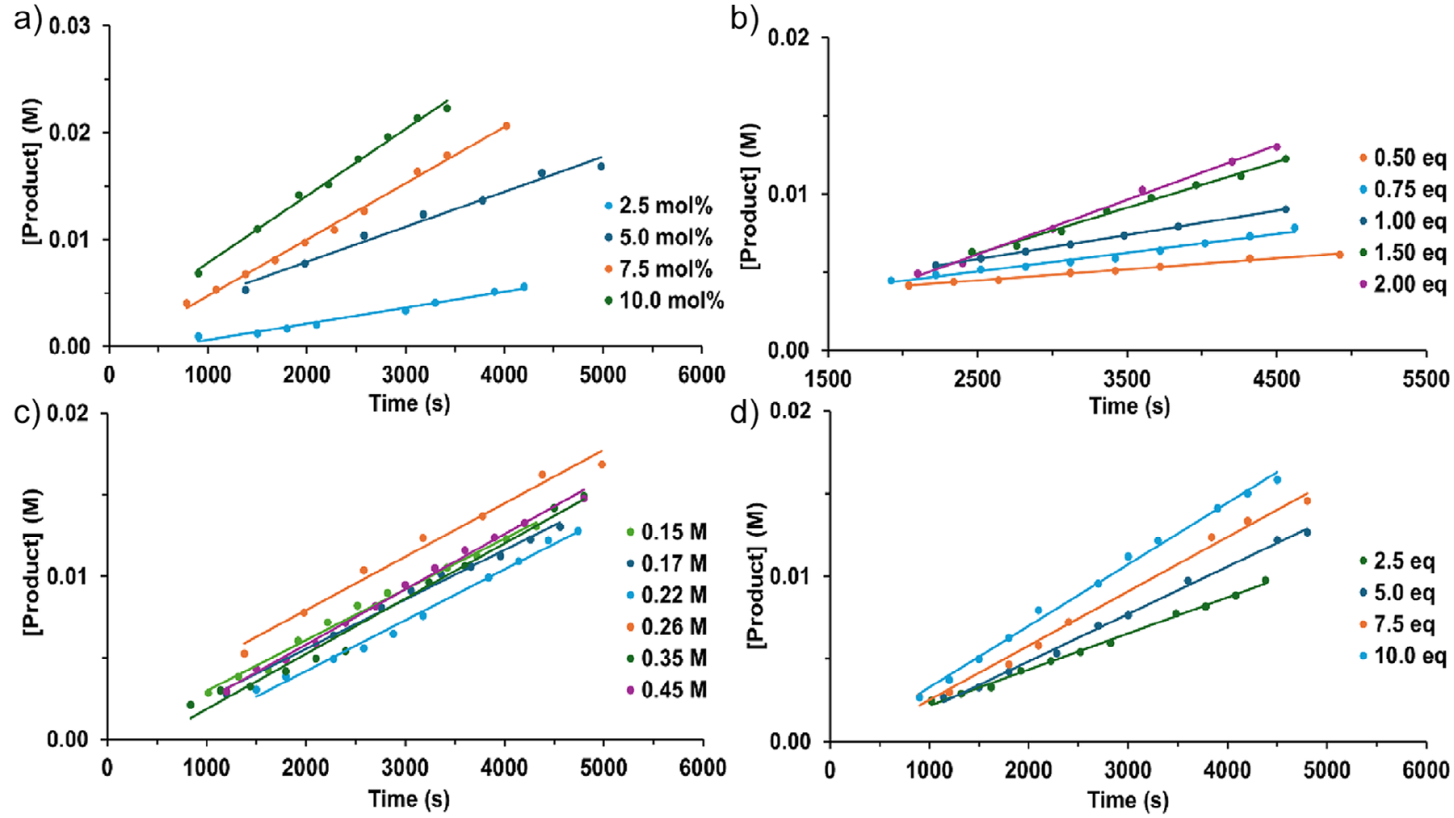
a) Initial rates at different La^NTMS^ concentrations. b) Initial rates at different PhSiH_3_ concentrations. c) Initial rates at different hexanamide concentrations. d) Initial rates at excess PhSiH_3_ concentrations.

**Figure 2:**
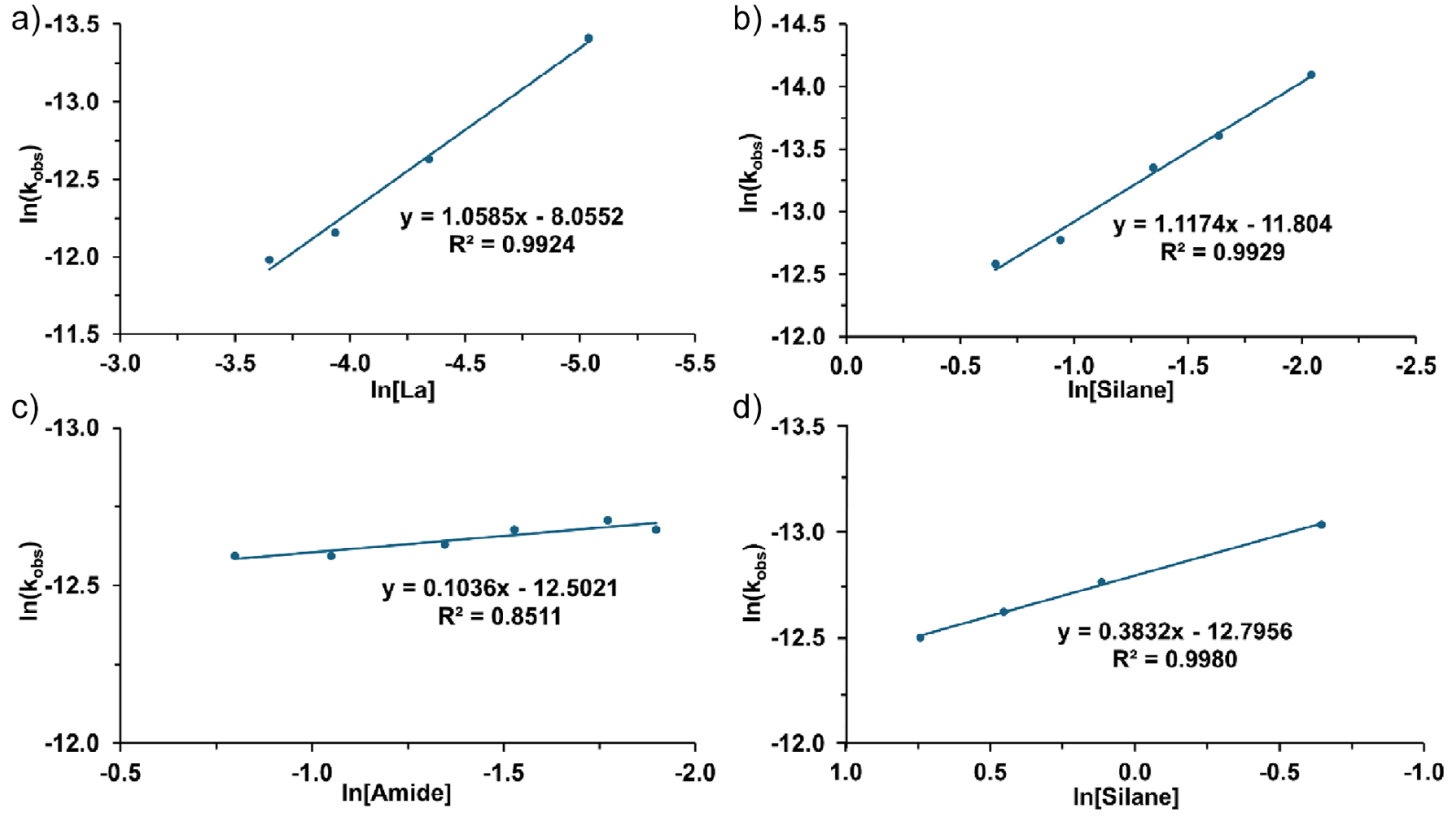
a) van't Hoff plot for the reaction rate law order in [La^NTMS^]. b) van't Hoff plot for the reaction rate law order in [PhSiH_3_]. c) van't Hoff plot for the reaction rate law order in [hexanamide]. d) Van't Hoff plot for the reaction rate law order in excess [PhSiH_3_].

To obtain further mechanistic insight, the rate law for catalytic hexanamide conversion to the corresponding nitrile was studied by an initial rate analysis over range of La^NTMS^ catalyst, hexanamide, and PhSiH_3_ concentrations (Figures 1 and 2).^[^ ^58^, ^59^ ^]^ Under the present conditions, the rate law is first‐order in [Lanthanum] and [Silane], and zero‐order in [Amide], as in Equation 2, presumably involving turnover‐limiting silane attack of on a La‐bound/activated amide.^[^ ^60^ ^]^ It is also suggested that one Si─H bond is cleaved in the transition state considering that PhSiH_3_ was previously reported by DFT calculation to undergo dehydrogenation via a single Si─H bond in the highest energy transition state.^[^ ^28^ ^]^ Note that if [silane] is in very large stochiometric excess, especially in neat catalytic reactions, the impact on reaction rate is diminished, ultimately tending to zero‐order in silane. In the present case, the rate law order in [Ph‐SiH_3_] was determined to be 0.38 as shown in Figure 2d, and first‐order in [silane] when it serves as the limiting reagent (<2 equiv.). Importantly, the reaction rate is solely dependent on [La[N(SiMe_3_)_2_]_3_] under neat conditions with excess silane.

(2) $$\mathrm{rate}=k{\left[\mathrm{Amide}\right]}^{0}{\left[La^{\mathrm{NTMS}}\right]}^{1}{\left[\mathrm{Sila}\mathrm{ne}\right]}^{1}$$

### Reaction Stoichiometry. Fate of PhSiH_3_ with Multiple Si─H Bonds

**Scheme 3:**
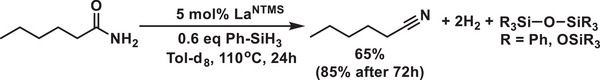
Hexanamide conversion to n‐hexylnitrile using sub‐stoichiometric PhSiH_3__._reaction conditions: 87.0 µmol hexanamide, 5 mol% pre‐catalyst loading, 0.6 equiv. PhSiH_3_, 0.5 mL tol‐d_8_, 110 °C, 18 h, inert atmosphere. Yields assayed by 1H NMR with mesitylene internal standard.

The present silane sources have multiple Si─H bonds available under either saturation or sub‐stoichiometric conditions. Intuitively, all the Si─H bonds are available for conversion when silane is the limiting reagent, and, in contrast, its impact on the reaction rate is invariant if silane is in large stoichiometric excess. The assumption that every Si─H bond can be utilized aligns with the ^1^H NMR spectroscopic observations in Scheme 3, where the reaction is pushed to near full conversion with 0.60 equiv. of PhSiH_3_ yielding polymeric phenyl siloxanes. Thus, ^1^H NMR identifies 2% unreacted PhSiH_3_, and broad signals between 6.5‐8 ppm assignable to polymeric siloxanes.^[^ ^61^ ^]^ The ^29^Si NMR spectra only identify trace amounts of unreacted PhSiH_3_ as well as ligand‐derived HN(SiMe_3_)_2_, and no signal for PhH_2_Si‐O‐SiH_2_Ph validating that essentially all Si─H bonds of PhSiH_3_ are consumed (see Figure S84).

### Activation Parameters

**Figure 3:**
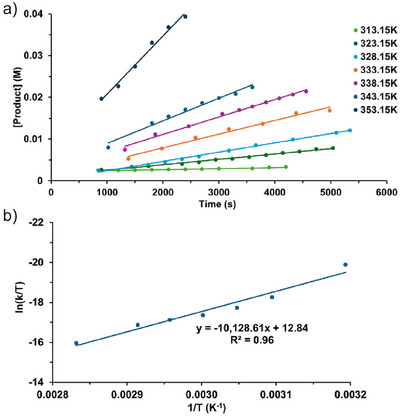
a) Temperature variation of initial rates of La^NTMS^‐catalyzed hexanamide conversion by PhSiH_3_. b) Eyring plot of the data.

For La^NTMS^‐catalyzed hexanamide conversion by PhSiH_3_, initial reaction rates were measured over a temperature range (Figure 3a), and the resulting Eyring analysis yields Δ*H* ^‡^ = 20.1 (1.9) kcal mol^−1^, Δ*S* ^‡^ = −21.7 (5.7) cal mol^−1^K^−1^(Figure 3b), while an Arrhenius analysis yields *E*_a_ = 20.8 (1.9) kcal mol^−1^(Figure S86), and the large negative Δ*S* ^‡^ = −21.7 (5.7) cal mol^−1^K^−1^ indicates a highly ordered transition state.^[^ ^62^ ^]^

## Isolation and Characterization of a La‐Acetamidate Active Species

**Scheme 4:**
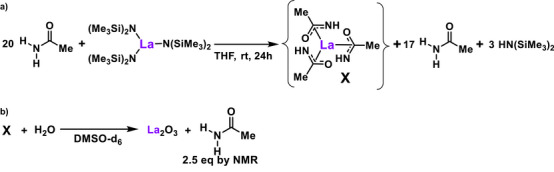
a) Proposed formation of a La‐trisamidate‐type species in concert with catalyst generation. b) Hydrolysis of La‐acetamidate species in wet DMSO‐d_6_^.^Conditions: 8.3 mg X in wet DMSO‐d_6_ (0.7 mL).

Experiments were next conducted to survey the catalyst resting state structure and activity. As shown in Scheme 4a, adding 20 equiv. of acetamide to 1 equiv. La[N(SiMe_3_)_2_]_3_ in THF yielded a La‐containing white powder after removing unreacted acetamide and HN(SiMe_3_)_2_ ligand under vacuum (Supporting Information Page 85). Full ligand exchange might theoretically yield a mononuclear complex similar to **X** or variously ligated mono‐nuclear species.

**Figure 4:**
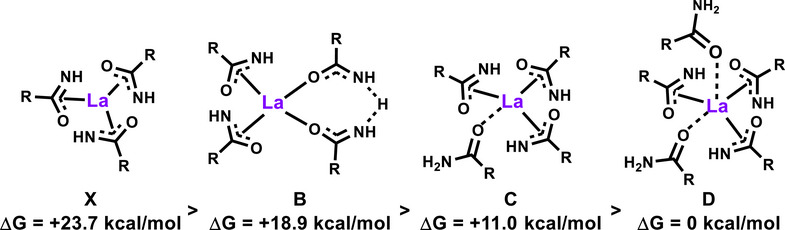
DFT computed Gibbs free energies for variously ligated mononuclear La‐amidate complexes.

**Figure 5:**
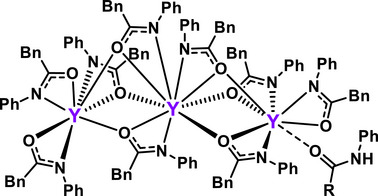
Single crystal trimetallic Y‐N‐phenylbenzylamidate structure reported by Zhou and coworkers.^[^ ^42^ ^]^

To survey these possibilities DFT screening was carried out and the relative stabilities are shown in Figure 4. Also plausible are polynuclear complexes such as **Y_3_(L_9_)LH** (Figure 5), similar to structure **C**, where L ═ N‐phenylbenzylamidate characterized by single‐crystal x‐ray diffraction.^[^ ^63^, ^64^, ^65^ ^]^ However, inductively coupled plasma optical emission spectrometry and combustion C,H,N analysis of **X** suggest a different stoichiometry, LaC_5_H_10.5_O_3_N_2.5_, for the isolated species (Supporting Informatin page 87). Furthermore, an ^1^H NMR hydrolysis titration (Scheme 4b) indicates that ∼2.5 equiv. of acetamide are released from each La^+3^ center, aligning with the above analytical data and suggesting a more O‐rich species. ^1^H and ^13^C NMR are not definitive and to date this material has resisted all attempts to grow diffraction‐quality crystals.

### Catalytic Properties of La‐Acetamidate Species **X**

**Scheme 5:**
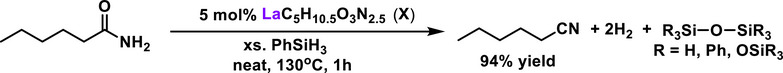
Silylative conversion of hexanamide to n‐hexylnitrile catalyzed by La‐acetamidate complex X.

The potential catalytic activity of the isolated La‐acetamidate **X** was next screened using the silylative conversion of hexanamide to *n*‐hexylnitrile under the conditions of Table 3, and afforded a 94% yield, comparable to that using La^NTMS^ under identical conditions (Scheme 5). Note that La‐acetamidate species **X** immediately dissolves in PhSiH_3_, suggesting a facile ligand exchange with hexanamide, yielding a catalytically‐active La‐trisamidate, and that any additional bound oxide ligands are removed by the silane.

Conditions: 87.0 µmol hexanamide, 5 mol% La‐trisacetamidate, excess PhSiH_3_ (600 mg), 130 °C, 1 h, inert atmosphere. Yields assayed by ^1^H NMR with a mesitylene internal standard.

## DFT Analysis of the Catalytic Cycle

**Figure 6:**
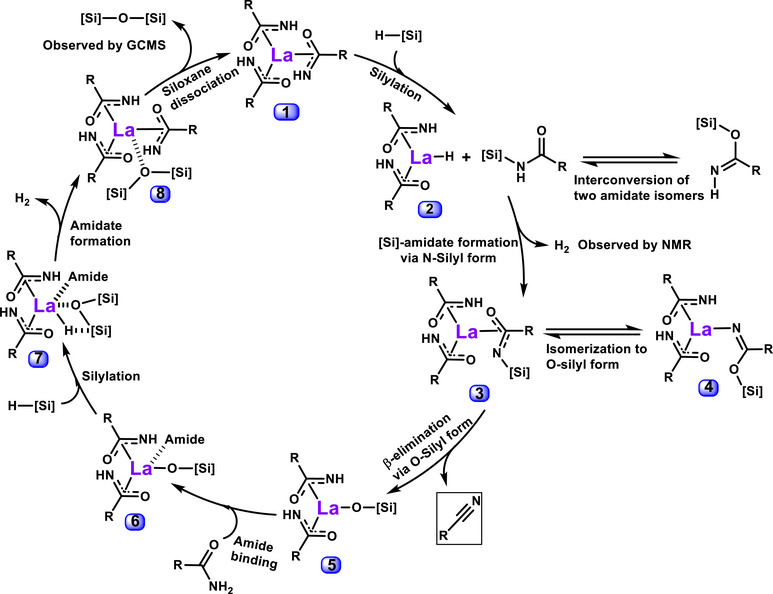
DFT‐computed catalytic cycle for primary amide to nitrile conversion. Two additional bound amide ligands are omitted for clarity and included in the representation La. Phenylsilane is abbreviated as [Si].

**Figure 7:**
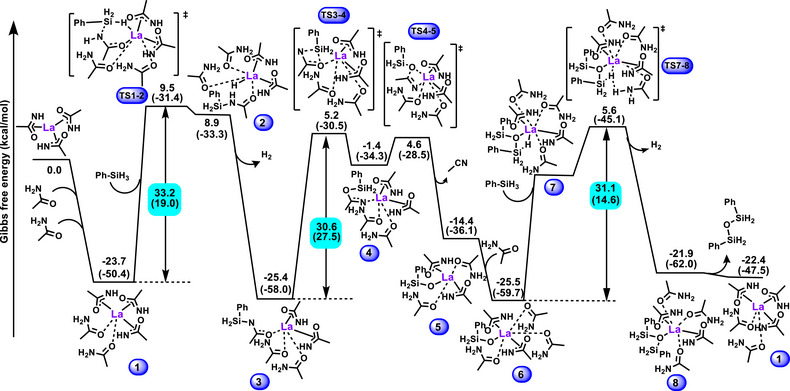
DFT computed transition state and intermediate energetics. For the three light‐blue highlighted endergonic steps, the values without parentheses refer to calculated Gibbs Free Energy of Activation (***ΔG*** ^‡^), and the values in parentheses refer to calculated Enthalpy of Activation (***ΔH*** ^‡^).

Based on the chemoselection and kinetic data in hand, it is possible to propose a catalytic cycle for the silylative conversion of primary amides to nitriles which can then be probed by DFT analysis (Figures 6 and 7). Starting from the putative La‐trisamidate active catalyst **1**, a silane undergoes La‐assisted silylation forming interconverting N‐silyl and O‐silyl imidates. DFT computation reveals that the N‐silyl form is more stable by 10 kcal mol^−1^ and then forms six‐membered transition state **TS1‐2** with La‐H intermediate **2** to eliminate H_2_, which is observed by ^1^H NMR at 4.5 ppm. Continuing from intermediate **3**, the N‐silyl form slowly isomerizes via **TS3‐4** to generate O‐silyl species **4**, which then undergoes β‐elimination to eliminate the nitrile product and generating La‐O‐Si species **5**. The energy required for the N‐silyl to O‐silyl isomerization is lower than that of the first endergonic step, hence does not affect the overall system kinetics and thermodynamics (Figure 7). Another free amide can coordinate to the La center to stabilize intermediate **5** to yield **6**. Next, another silane that enters and undergoes H─Si⋯La─O σ‐bond metathesis via **7**, generating La‐H **TS7‐8** and the irreversible formation of active catalyst **1** via H_2_ + siloxane product release, observable by GCMS (Figure S82). Insertion of the silane into intermediate **6** to eventually yield siloxane is another highly endergonic step similar to its insertion into **1**.

From the present energy profile (Figure 7), the turn‐over determining intermediate (TDI) is located at intermediate **1**, and the turn‐over determining transition state (TDTS) is located a **TS1‐2**, resulting in first‐order behavior in [silane] and [La] species, as well as zero‐order in [primary amide]. The relatively high Δ*G* ^‡^ =  33.2 kcal mol^−1^ aligns well with the experimental observations that reaction proceeds slowly in solutions with stoichiometric silane, while efficient turnover rates require high silane concentrations. The transition from first‐order to zero‐order with respect to [silane] observed at high silane concentrations implies that high silane concentrations lower the energy profile of the first and third steps (where [silane] enters), but not that of the second step, which then becomes the rate‐determining step. Moreover, the computed Δ*H* ^‡^ =  19.0 kcal mol^−1^ for the turn‐over determining step coincides well with the experimentally determined activation parameter, Δ*H* ^‡^ =  20.1 (1.9) kcal mol^−1^, further validating the consistency of the computational results and experimental observations. Lastly, kinetic isotope effect (KIE) experiments with PhSiH_3_ versus PhSiD_3_ yield k_Si‐H_/k_Si‐D _= 1.4(0.1) at 60 °C, indicating a primary KIE with the cleavage of Si─H bond at the rate‐determining step (Figure S87). This KIE value is consistent with other reported values involving Si─H bond cleavage.^[^ ^66^, ^67^, ^68^, ^69^ ^]^ The DFT calculated KIE values are 1.89 for the first and 1.43 for the third endergonic steps, and 0.94 for the second step. Note that considering the similar energy barriers of three endergonic steps, a combination of them contributes to the computed overall KIE value which aligns with the present experimental KIE value of 1.4(0.1).^[^ ^70^ ^]^

## Conclusion

These results demonstrate the efficacy of readily available Ln[N(SiMe_3_)_2_]_3_ precatalysts in the silylative conversion of primary amides to nitriles with good selectivity and activity. The lanthanide trisamides convert to lanthanide trisamidates under catalytic conditions, exhibiting broad substrate functional group tolerance and selectivity in the generation of nitrile functionalities at 5 mol% catalyst loading with PhSiH_3_ and TMS‐O‐[Si(H)(Me)‐O‐]_n_‐TMS under neat conditions. Both experimental kinetic mechanistic analysis and DFT computation provide in‐depth insight into the overall catalytic pathway. Based on the kinetic rate law of *rate*  =  *k*[*Amide*]^0^[*La^NTMS^*]^1^[*Silane*]^1^ and isotope effect data, as well as isolation of a catalytically active species, a complete catalytic cycle is proposed, with the DFT calculations showing how the La‐trisamidate catalyst undergoes silylation to mediate the transformation of diverse primary amides to nitriles with siloxane co‐formation and release of H_2_. Therefore, given the efficiency and robustness of the lanthanide‐organic mediated silyative conversion of primary amides to nitriles, it is believed that lanthanide catalysis can play an important role in future heteroatom catalysis.

## Conflict of Interests

The authors declare no conflict of interest.

## Supporting information



Supporting Information

## Data Availability

The data that support the findings of this study are available in the supplementary material of this article.
