# Supplementary material for: Silylative Amide to Nitrile Conversion Mediated by Simple Lanthanide–Organoamides: Scope and Mechanism
Source: Angew Chem Int Ed Engl. 2025 Sep 26;64(46):e202513996. doi: 10.1002/anie.202513996 (PMC12603988; doi:10.1002/anie.202513996)
Supplement: Supplementary file 1 — Supporting Information [file ANIE-64-e202513996-s001.docx]

**Silylative Amide to Nitrile Conversion Mediated by Simple Lanthanide--Organoamides: Scope and Mechanism**

**Supporting Information**

Zhiyu Feng^[a]^, Qingheng Lai^[a]^, Yuang Wang^[a]^, Alessandro Motta*^[b]^, Yosi Kratish*^[a]^, Tobin J. Marks*^[a]^

*[a] Department of Chemistry, Northwestern University, Evanston, Illinois 60208-3113, United States;*

*[b] Dipartimento di Scienze Chimiche, Università di Roma “La Sapienza” and INSTM, UdR Roma, I-00185 Roma, Italy;*

Table of Contents

[1. General Information 2](#_Toc208240045)

[1.1 General Materials Information 2](#_Toc208240046)

[1.2 General Analytical Instrumentation Information 2](#_Toc208240047)

[1.3 Procedure for General NMR Scale Reactions with and without Solvent 3](#_Toc208240048)

[1.4 Procedure for Preparative (250 mg) and Gram (1.0 g) Scale Synthesis 3](#_Toc208240049)

[2. Experimental Details and Characterization of Products 4](#_Toc208240050)

[2.1 Lanthanide Ion Size and Ancillary Ligand Effects (Table 1) 4](#_Toc208240051)

[2.2 Reaction Condition Optimization (Table 2) 5](#_Toc208240052)

[2.3 Substrate Scope for Alkyl Amides (Table 3) 7](#_Toc208240053)

[2.4 Substrate Scope of Aromatic Amides (Table 4) 18](#_Toc208240054)

[2.5 Gram Scale Synthesis 40](#_Toc208240055)

[3. Experimental Details and Data for Mechanistic Study 43](#_Toc208240056)

[3.1 Initial-Rate Kinetic Analysis 43](#_Toc208240057)

[3.2 Investigation of the Siloxane Byproducts 75](#_Toc208240058)

[3.3 Determination of Activation Parameters 78](#_Toc208240059)

[3.4 Kinetic Isotope Effect Experiment for Phenylsilane 80](#_Toc208240060)

[3.5 Protonolysis Experiments 83](#_Toc208240061)

[3.6 Isolation and Characterization of the La-acetamidate active species 85](#_Toc208240062)

[3.7 Computational Details 90](#_Toc208240063)

[3.8 Cartesian Coordinates (Å) of all Presented Species 92](#_Toc208240064)

[References 110](#_Toc208240065)

1. General Information

## 1.1 General Materials Information

Considering the air and moisture sensitivity of organolanthanides employed in this study, unless noted otherwise, manipulations of all materials were conducted with rigorous exclusion of O_2_ and moisture in flame or oven dried Schlenk-type glassware, J-Young NMR tubes that connected to a high vacuum manifold (10^-6^ Torr), or an Ar filled MBraun glovebox with a high-capacity recirculator (<1 O_2_ ppm) was used to store and prepare starting materials. Argon (Airgas) was purified by passing a MnO column to remove O_2_ and a column of Davison 3Å molecular sieves to remove water before use in the high vac line. Amide substrates were purchased from Sigma Aldrich or TCI-America and purged under high-vac line overnight before bringing into the glovebox for use. Phenylsilane (PhSiH_3_) was purchased from Sigma Aldrich, polymethylhydrosiloxane (PMHS) was purchased from Alfa Aesar and dimethoxymethylsilane (DMMS, moisture sensitive) was purchased from TCI America. All silane materials were further dried over activated 3Å molecular sieves, purchased from ThermalScientificChemicals, before use. Tris(N,N-bis(trimethylsilyl)amide)lanthanum(III), min. 98% (La[N(SiMe_3_)_2_]_3_) was purchased from Strem Chemicals and used as supplied. Cp*_2_LuCH(TMS)_2_ was prepared following procedures reported previously.^[1]^ Toluene-d_8_ was purchased from Sigma Aldrich and stored over Na/K alloy in vacuo and filtered in glovebox before use. THF was purchased from Sigma Aldrich, distilled from calcium hydride and stored in a dry Schlenk tube before use. SiliaFlash® Irregular Silica Gels (F60, 40-63 µm, 230-400 mesh, 60 Å) was used for column chromatography with HPLC grade methylene chloride (DCM), ethyl acetate (EtOAc) and hexane were purchased from Thermal Scientific Chemicals.

## 1.2 General Analytical Instrumentation Information

^1^H and ^13^C NMR were recorded on Bruker Avance III HD (BBFO Smart Probe, FT, 400 MHz, 1H; 77 400 MHz, 13C; 101 MHz, 11B; 128 MHz), Bruker Avance III 600 (BBFO Smart Probe, 600 MHz, 78 1H; 600, 13C; 151, 11B; 193 MHz)), Bruker Avance III HD (TXO 5mm Prodigy Probe, 500MHz, 79 1H; 1200, 13C; 700). Internal solvent resonances are used for referencing the chemical shifts. J. Young sample tubes were used for NMR measurement on air sensitive samples. High resolution mass spectra (HRMS) were acquired on the Agilent 6890 Series GCMSD using processed using Enhanced Chemstation Version E.02.02.1431 for data acquisition. Data was processed using Agilent Chemstation. The mass spectrometer was configured with an Agilent 7683 Series Injector and auto sampler, and 5973 Network Mass Selective Detector. Analytical TLC was performed using Silicycle SilicaPlate® glass-backed extra-hard-layer TLC plates (60 Å, 250 µm thickness, 20x20 cm, UV-254 indicator) and visualization with 254 nm light. ICP-OES service was provided by Rebecca Sponenburg at Northwestern University, and Midwest Microlab provided services for elemental analysis.

## 1.3 Procedure for General NMR Scale Reactions with and without Solvent

In a glovebox, the Amide substrate and corresponding catalytic percentage of La[N(SiMe_3_)_2_]_3_ were weighed and transferred to the J. Young NMR tube. The silane, the solvent (0.5 mL) and mesitylene (14.7 $\mu$L) were syringed into the NMR tube. The tube was then sealed and transferred out of the glovebox to be placed in an oil bath at designated temperatures for indicated reaction times. The sample liquid level was fully submerged below the oil bath level to ensure accurate heating. The resulting mixture was analyzed by in situ by ^1^H and ^13^C NMR. For reactions without solvent, stoichiometrically excess silane (600 mg) was added. A DMSO-d_6_ capillary was placed inside the NMR tube for in situ NMR calibration and measurement. Other reagents and procedures remained the same.

## 1.4 Procedure for Preparative (250 mg) and Gram (1.0 g) Scale Synthesis

In the glovebox, to a high-pressure glass reactor was added the Amide substrate (250 mg or 1 g), 5 mol% La[N(SiMe_3_)_2_]_3_ catalyst, desired amount of silane (stoichiometric or excess for neat conditions), and Na/K dried toluene solvent (20 mL) if needed. The reactor was sealed and transferred out of the glovebox into an oil bath for heating, and the reaction mixture was allowed to react for measured amounts of time. After the reaction was complete, the reactor cap was carefully unscrewed to release H_2_ gas, and then DCM (2 to 5 mL) was directly added to the mixture to dissolve and extract the product for column chromatography. Ethyl acetate and hexane were used as eluting solvents to isolate the pure product by column chromatography.

2. Experimental Details and Characterization of Products

## 2.1 Lanthanide Ion Size and Ancillary Ligand Effects (Table 1)

NMR characterization for the product *n*-hexylnitrile. All data agree with literature reported spectrum.^[2]^ A representative NMR spectrum for the product of Table 1, Entry 1 is provided as below.

**Table 1, Entry 1**

^1^H NMR (500 MHz, Toluene-*d*_8_) δ 1.41 (t, *J*=7.0 Hz, 2H), 1.03 – 0.82 (m, 6H), 0.70 (t, *J*=7.0 Hz, 3H).

^13^C NMR (126 MHz, Toluene-*d*_8_) δ 119.07, 30.85, 25.26, 22.07, 16.49, 13.78.


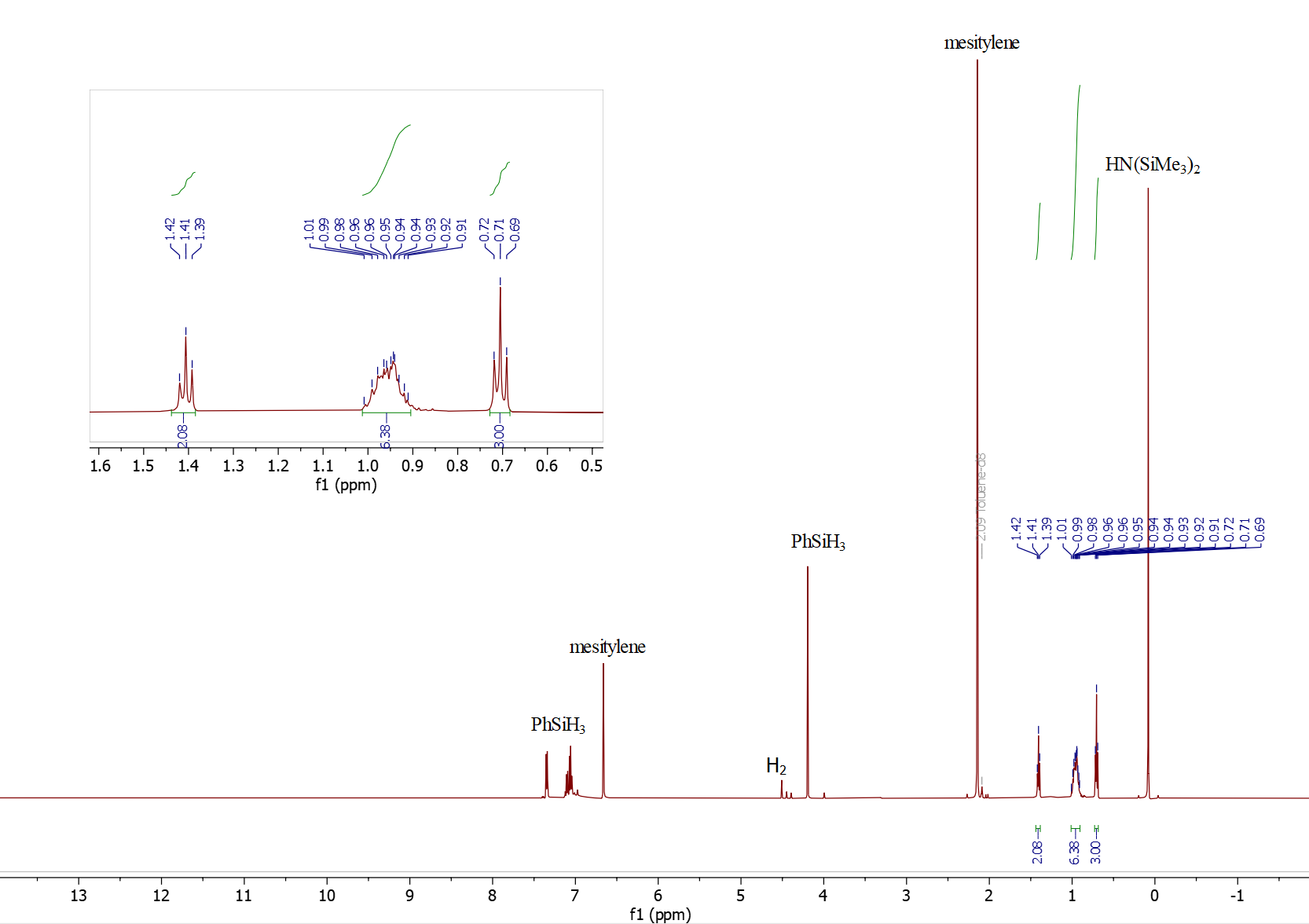


Figure S1. ^1^H NMR (500 MHz, Toluene-*d*_8_) spectrum of the n-hexylnitrile in Table 1, entry 1.

Figure S2. ^13^C NMR (126 MHz, Toluene-*d*_8_) spectrum of the n-hexylnitrile in Table 1, entry 1.

## 2.2 Reaction Condition Optimization (Table 2)

All NMR characterization for the product n-hexylnitrile. A representative NMR spectrum for the product of Table 2, Entry 1 is provided as below.

**Table 2, Entry 1**

^1^H NMR (500 MHz, Toluene-*d*_8_) δ 1.40 (t, *J* = 7.0 Hz, 2H), 1.02 – 0.87 (m, 6H), 0.70 (t, *J* = 7.0 Hz, 3H).

^13^C NMR (126 MHz, Toluene-*d*_8_) δ 119.08, 30.84, 25.24, 22.06, 16.49, 13.78.

Figure S3. ^1^H NMR (500 MHz, Toluene-*d*_8_) spectrum of the n-hexylnitrile in Table 2, entry 1.

Figure S4. ^13^C NMR (126 MHz, Toluene-*d*_8_) spectrum of the n-hexylnitrile in Table 2, entry 1.

## 2.3 Substrate Scope for Alkyl Amides (Table 3)

**3a**

^1^H NMR (400 MHz, DMSO-*d*_6_) δ 1.31 (t, *J* = 6.7 Hz, 2H), 0.89 – 0.70 (m, 6H), 0.47 (t, *J* = 6.7 Hz, 3H).

^13^C NMR (101 MHz, DMSO-*d*_6_) δ 117.80, 29.87, 24.28, 21.09, 15.70, 12.92.

**3b**^[3]^

^1^H NMR (600 MHz, DMSO-*d*_6_) δ 1.29 (t, *J* = 6.8 Hz, 2H), 0.83 – 0.76 (m, 4H), 0.38 (t, *J* = 7.2 Hz, 3H).

^13^C NMR (151 MHz, DMSO-*d*_6_) δ 117.76, 26.48, 20.92, 15.43, 12.28.

**3c**^[4]^

^1^H NMR (600 MHz, DMSO-*d*_6_) δ 0.64 (s, 1H).

^13^C NMR (151 MHz, DMSO-*d*_6_) δ 123.83, 27.14, 27.12.

**3d**^[5]^

^1^H NMR (600 MHz, DMSO-*d*_6_) δ 1.25 (t, *J* = 7.1 Hz, 2H), 0.82 (q, *J* = 7.2 Hz, 2H), 0.40 (t, *J* = 7.4 Hz, 3H).

^13^C NMR (151 MHz, DMSO-*d*_6_) δ 117.61, 18.23, 17.53, 12.14.

**3e**^[6]^

^1^H NMR (600 MHz, DMSO-*d*_6_) δ 1.23 (q, *J* = 7.6 Hz, 2H), 0.39 (t, *J* = 7.6 Hz, 3H).

^13^C NMR (151 MHz, DMSO-*d*_6_) δ 118.65, 9.40, 9.31.

**3f**^[4]^

^1^H NMR (400 MHz, DMSO-*d*_6_) δ 0.70 (s, 3H).

^13^C NMR (101 MHz, DMSO-*d*_6_) δ 114.35, 1.79.

**3g**^[7]^

^1^H NMR (600 MHz, DMSO-*d*_6_) δ 1.46 – 1.41 (m, 9H), 1.15 – 1.05 (m, 6H).

^13^C NMR (151 MHz, DMSO-*d*_6_) δ 123.29, 39.00, 34.81, 29.12, 26.36.

**3h**^[8]^

^1^H NMR (600 MHz, DMSO-*d*_6_) δ 1.68 – 1.61 (m, 2H), 1.16 – 1.03 (m, 6H).

^13^C NMR (151 MHz, DMSO-*d*_6_) δ 120.57, 28.60, 26.91, 24.37, 23.16.

**3i**^[9]^

Brown/Pink viscous oil

^1^H NMR (600 MHz, Chloroform-*d*) δ 7.77 (d, *J* = 7.9 Hz, 1H), 7.57 (s, 1H), 7.42 – 7.29 (m, 3H), 3.86 (s, 3H).

^13^C NMR (151 MHz, Chloroform-*d*) δ 136.16, 135.65, 127.96, 124.01, 122.29, 120.05, 116.05, 110.44, 85.73, 33.77.

Figure S5. ^1^H NMR (400 MHz, DMSO-*d*_6_) spectrum of 3a.

Figure S6. ^13^C NMR (101 MHz, DMSO-*d*_6_) spectrum of 3a.

Figure S7. ^1^H NMR (600 MHz, DMSO-*d*_6_) spectrum of 3b.

Figure S8. ^13^C NMR (151 MHz, DMSO-*d*_6_) spectrum of 3b.

Figure S9. ^1^H NMR (600 MHz, DMSO-*d*_6_) spectrum of 3c.

Figure S10. ^13^C NMR (151 MHz, DMSO-*d*_6_) spectrum of 3c.

Figure S11. ^1^H NMR (600 MHz, DMSO-*d*_6_) spectrum of 3d.

Figure S12. ^13^C NMR (151 MHz, DMSO-*d*_6_) spectrum of 3d.

Figure S13. ^1^H NMR (600 MHz, DMSO-*d*_6_) spectrum of 3e.

Figure S14. ^13^C NMR (151 MHz, DMSO-*d*_6_) spectrum of 3e.

Figure S15. ^1^H NMR (400 MHz, DMSO-*d*_6_) spectrum of 3f.

Figure S16. ^13^C NMR (101 MHz, DMSO-*d*_6_) spectrum of 3f.

Figure S17. ^1^H NMR (600 MHz, DMSO-*d*_6_) spectrum of 3g.

Figure S18. ^13^C NMR (151 MHz, DMSO-*d*_6_) spectrum of 3g.

Figure S19. ^1^H NMR (600 MHz, DMSO-*d*_6_) spectrum of 3h.

Figure S20. ^13^C NMR (151 MHz, DMSO-*d*_6_) spectrum of 3h.

Figure S21. ^1^H NMR (600 MHz, DMSO-*d*_6_) spectrum of 3i.

Figure S22. ^13^C NMR (151 MHz, DMSO-*d*_6_) spectrum of 3i.

## 2.4 Substrate Scope of Aromatic Amides (Table 4)

**4a**^[10]^

^1^H NMR (600 MHz, DMSO-*d*_6_) δ 7.55 (d, *J* = 7.6 Hz, 2H), 7.51 (t, *J* = 7.6 Hz, 1H), 7.40 (t, *J* = 7.8 Hz, 2H).

^13^C NMR (151 MHz, DMSO-*d*_6_) δ 131.41, 131.17, 128.34, 116.81, 113.07.

**4b**^[11]^

Reaction time: 24h

^1^H NMR (600 MHz, DMSO-*d*_6_) δ 7.47 (d, *J* = 8.8 Hz, 2H), 6.89 (d, *J* = 8.8 Hz, 2H), 3.79 (s, 3H).

^13^C NMR (151 MHz, DMSO-*d*_6_) δ 161.94, 132.86, 117.04, 113.95, 104.70, 54.29.

Reaction time: 48h

^1^H NMR (500 MHz, DMSO-*d*_6_) δ 7.48 (d, *J* = 8.8 Hz, 2H), 6.90 (d, *J* = 8.8 Hz, 2H), 3.80 (s, 3H).

^13^C NMR (126 MHz, DMSO-*d*_6_) δ 161.94, 132.86, 117.02, 113.96, 104.72, 54.29.

**4c**^[12]^

^1^H NMR (600 MHz, DMSO-*d*_6_) δ 7.31 – 7.25 (m, 1H), 7.14 – 7.10 (m, 2H), 7.06 – 7.02 (m, 2H), 3.75 (s, 3H).

^13^C NMR (151 MHz, DMSO-*d*_6_) δ 159.24, 129.43, 123.38, 118.08, 116.82, 115.79, 113.71, 54.25.

**4d**^[13]^

^1^H NMR (600 MHz, DMSO-*d*_6_) δ 7.46 – 7.40 (m, 2H), 6.95 – 6.86 (m, 2H), 3.83 (s, 3H).

^13^C NMR (151 MHz, DMSO-*d*_6_) δ 160.42, 132.90, 132.86, 119.94, 114.17, 110.47, 102.60, 54.75.

**4e**^[13]^

^1^H NMR (500 MHz, DMSO-*d*_6_) δ 8.08 (s, 1H), 7.92 – 7.71 (m, 3H), 7.62 – 7.42 (m, 3H).

^13^C NMR (126 MHz, DMSO-*d*_6_) δ 133.95, 132.82, 131.90, 128.37, 128.00, 127.70, 127.33, 126.81, 125.69, 117.23, 109.95.

**4f**^[11]^

^1^H NMR (500 MHz, DMSO-*d*_6_) δ 7.61 – 7.54 (m, 2H), 7.11 (t, *J* = 8.5 Hz, 2H).

^13^C NMR (126 MHz, DMSO-*d*_6_) δ 165.21, 163.18, 133.67, 133.60, 116.07, 116.02, 115.89, 109.10, 109.07.

**4g**^[11]^

^1^H NMR (500 MHz, DMSO-*d*_6_) δ 7.49 (d, *J* = 8.6 Hz, 2H), 7.39 (d, *J* = 8.6 Hz, 2H).

^13^C NMR (126 MHz, DMSO-*d*_6_) δ 138.40, 132.43, 128.90, 116.01, 111.28.

**4h**^[11]^

^1^H NMR (600 MHz, DMSO-*d*_6_) δ 7.40 – 7.32 (m, 2H), 7.25 – 7.20 (m, 2H).

^13^C NMR (151 MHz, DMSO-*d*_6_) δ 162.61, 160.95, 130.33, 130.27, 127.15, 127.12, 119.20, 119.07, 118.28, 118.12, 115.69, 115.68, 114.48.

**4i**^[11]^

^1^H NMR (600 MHz, DMSO-*d*_6_) δ 7.55 – 7.48 (m, 2H), 7.47 – 7.43 (m, 1H), 7.35 (t, *J* = 8.9 Hz, 1H).

^13^C NMR (151 MHz, DMSO-*d*_6_) δ 134.75, 131.96, 131.04, 129.70, 129.27, 115.59, 114.48.

**4j**^[3]^

^1^H NMR (500 MHz, DMSO-*d*_6_) δ 7.42 (d, *J* = 8.2 Hz, 2H), 7.19 (d, *J* = 8.3 Hz, 2H), 2.37 (s, 3H).

^13^C NMR (126 MHz, DMSO-*d*_6_) δ 141.96, 131.09, 129.01, 116.99, 110.10, 20.79.

**4k**^[14]^

^1^H NMR (600 MHz, DMSO-*d*_6_) δ 7.47 (d, *J* = 8.5 Hz, 2H), 7.42 (d, *J* = 8.5 Hz, 2H), 1.33 (s, 9H).

^13^C NMR (151 MHz, DMSO-*d*_6_) δ 155.03, 131.00, 125.29, 117.01, 110.03, 30.28.

**4l**^[2]^

^1^H NMR (400 MHz, DMSO-*d*_6_) δ 2.17 (t, *J* = 7.1 Hz, 2H), 1.59 (p, *J* = 7.0 Hz, 2H), 1.39 (qt, *J* = 14.8, 7.5 Hz, 2H), 0.95 (t, *J* = 7.2 Hz, 3H).

^13^C NMR (101 MHz, DMSO-*d*_6_) δ 117.15, 30.15, 24.68, 21.26, 16.13, 13.00.

**4m**^[15]^

Colorless viscous oil

^1^H NMR (500 MHz, Chloroform-*d*) δ 7.26 (d, *J* = 7.1 Hz, 2H), 7.17 (d, *J* = 8.2 Hz, 2H), 3.88 (q, *J* = 7.3 Hz, 1H), 2.48 (d, *J* = 7.2 Hz, 2H), 1.93 – 1.81 (m, 1H), 1.65 (d, *J* = 7.2 Hz, 3H), 0.91 (d, *J* = 6.6 Hz, 6H).

^13^C NMR (126 MHz, Chloroform-*d*) δ 141.80, 134.43, 129.96, 126.58, 121.96, 45.10, 31.04, 30.32, 22.46, 21.58.

**4o**^[16]^

^1^H NMR (600 MHz, DMSO-*d*_6_) δ 8.98 – 8.88 (m, 1H), 8.23 – 8.10 (m, 2H), 7.80 – 7.72 (m, 1H), 7.69 – 7.63 (m, 1H), 7.56 – 7.50 (m, 1H).

^13^C NMR (151 MHz, DMSO-*d*_6_) δ 148.08, 147.73, 146.02, 129.82, 129.41, 127.61, 123.78, 123.06, 117.92, 113.55.

**4p**^[17]^

^1^H NMR (500 MHz, DMSO-*d*_6_) δ 8.79 – 8.74 (m, 1H), 8.10 – 8.01 (m, 2H), 7.75 (ddd, *J* = 6.9, 4.8, 1.8 Hz, 1H).

^13^C NMR (126 MHz, DMSO-*d*_6_) δ 151.28, 138.03, 132.67, 129.14, 127.93, 117.52.

**4q**^[18]^

^1^H NMR (600 MHz, DMSO-*d*_6_) δ 8.79 (s, 1H), 8.73 (d, *J* = 4.8 Hz, 1H), 7.80 (d, *J* = 7.9 Hz, 1H), 7.29 (dd, *J* = 8.0, 4.9 Hz, 1H).

^13^C NMR (151 MHz, DMSO-*d*_6_) δ 151.90, 151.68, 137.73, 122.48, 114.90, 110.08.

**4r**^[19]^

^1^H NMR (600 MHz, DMSO-*d*_6_) δ 7.48 (dd, *J* = 6.7, 4.4 Hz, 2H), 7.07 – 7.01 (m, 1H).

^13^C NMR (151 MHz, DMSO-*d*_6_) δ 135.60, 130.81, 126.73, 112.10, 110.54.

Figure S23. ^1^H NMR (600 MHz, DMSO-*d*_6_) spectrum of 4a.

Figure S24. ^13^C NMR (151 MHz, DMSO-*d*_6_) spectrum of 4a.

Figure S25. ^1^H NMR (600 MHz, DMSO-*d*_6_) spectrum of 4b (24h).

Figure S26. ^13^C NMR (151 MHz, DMSO-*d*_6_) spectrum of 4b (24h).

Figure S27. ^1^H NMR (500 MHz, DMSO-*d*_6_) spectrum of 4b (48h).

Figure S28. ^13^C NMR (126 MHz, DMSO-*d*_6_) spectrum of 4b (48h).

Figure S29. ^1^H (600 MHz, DMSO-*d*_6_) NMR spectrum of 4c.

Figure S30. ^13^C NMR (151 MHz, DMSO-*d*_6_) spectrum of 4c.

Figure S31. ^1^H (600 MHz, DMSO-*d*_6_) NMR spectrum of 4d.

Figure S32. ^13^C NMR (151 MHz, DMSO-*d*_6_) spectrum of 4d.

Figure S33. ^1^H NMR (500 MHz, DMSO-*d*_6_) spectrum of 4e.

Figure S34. ^13^C NMR (126 MHz, DMSO-*d*_6_) spectrum of 4e.

Figure S35. ^1^H NMR (500 MHz, DMSO-*d*_6_) spectrum of 4f.

Figure S36. ^13^C NMR (126 MHz, DMSO-*d*_6_) spectrum of 4f.

Figure S37. ^1^H NMR (500 MHz, DMSO-*d*_6_) spectrum of 4g.

Figure S38. ^13^C NMR (126 MHz, DMSO-*d*_6_) spectrum of 4g.

Figure S39. ^1^H (600 MHz, DMSO-*d*_6_) NMR spectrum of 4h.

Figure S40. ^13^C NMR (151 MHz, DMSO-*d*_6_) spectrum of 4h.

Figure S41. ^1^H NMR (600 MHz, DMSO-*d*_6_) spectrum of 4i.

Figure S42. ^13^C NMR (151 MHz, DMSO-*d*_6_) spectrum of 4i.

Figure S43. ^1^H NMR (500 MHz, DMSO-*d*_6_) spectrum of 4j.

Figure S44. ^13^C NMR (126 MHz, DMSO-*d*_6_) spectrum of 4j.

Figure S45. ^1^H NMR (600 MHz, DMSO-*d*_6_) spectrum of 4k.

Figure S46. ^13^C NMR (151 MHz, DMSO-*d*_6_) spectrum of 4k.

Figure S47. ^1^H NMR (400 MHz, DMSO-*d*_6_) spectrum of 4l.

Figure S48. ^13^C NMR (101 MHz, DMSO-*d*_6_) spectrum of 4l.

Figure S49. ^1^H NMR (500 MHz, DMSO-*d*_6_) spectrum of 4m.

Figure S50. ^13^C NMR (126 MHz, DMSO-*d*_6_) spectrum of 4m.

Figure S51. ^1^H NMR (600 MHz, DMSO-*d*_6_) spectrum of 4o.

Figure S52. ^13^C NMR (101 MHz, DMSO-*d*_6_) spectrum of 4o.

Figure S53. ^1^H NMR (600 MHz, DMSO-*d*_6_) spectrum of 4p.

Figure S54. ^13^C NMR (101 MHz, DMSO-*d*_6_) spectrum of 4p.

Figure S55. ^1^H NMR (600 MHz, DMSO-*d*_6_) spectrum of 4q.

Figure S56. ^13^C NMR (101 MHz, DMSO-*d*_6_) spectrum of 4q.

Figure S57. ^1^H NMR (600 MHz, DMSO-*d*_6_) spectrum of 4r.

Figure S58. ^13^C NMR (101 MHz, DMSO-*d*_6_) spectrum of 4r.

## 2.5 Gram Scale Synthesis

**Diphenylacetonitrile**^[20]^ (0.72 g, 78% yield)

White solid

^1^H NMR (600 MHz, Chloroform-*d*) δ 7.41 – 7.30 (m, 10H), 5.15 (s, 1H).

^13^C NMR (151 MHz, Chloroform-*d*) δ 136.03, 129.33, 128.39, 127.87, 119.80, 42.72.

**1-Naphthaleneacetonitrile**^[20]^ (0.70 g, 77% yield)

Yellow, viscous oil

^1^H NMR (600 MHz, Chloroform-*d*) δ 7.94 – 7.85 (m, 3H), 7.64 – 7.54 (m, 3H), 7.49 (t, *J* = 8.4 Hz, 1H), 4.14 (s, 2H).

^13^C NMR (151 MHz, Chloroform-*d*) δ 133.88, 130.93, 129.28, 129.21, 127.23, 126.62, 126.52, 125.91, 125.62, 122.54, 117.81, 21.87.

Figure S59. ^1^H NMR (600 MHz, Chloroform-*d*) spectrum of Diphenylacetonitrile.

Figure S60. ^13^C NMR (151 MHz, Chloroform-*d*) spectrum of Diphenylacetonitrile.

Figure S61. ^1^H NMR (600 MHz, Chloroform-*d*) spectrum of 1-Naphthaleneacetonitrile.

Figure S62. ^13^C NMR (151 MHz, Chloroform-*d*) spectrum of 1-Naphthaleneacetonitrile.

3. Experimental Details and Data for Mechanistic Study

## 3.1 Initial-Rate Kinetic Analysis

The kinetic analysis for the NMR scale reactions was conducted by collecting data points of product concentration before the substrate concentration was dramatically depleted, for which the substrate concentration is considered pseudo-zero-order. The spectrometer pulse delay (D1) of 20 s was set to avoid saturation, and a 90-degree pulse was applied to ^1^H NMR measurement to ensure maximum measurement signal.

Specifically following the same procedure as **1.3**, to a J-Young NMR tube was added various concentrations of hexanamide substrate, La[N(SiMe_3_)_2_]_3_ and PhSiH_3_. The toluene solvent (0.5 mL) and mesitylene internal standard (14.7 mg, 17 µL) were also syringed into the NMR tube. The Bruker Avance III 600 NMR instrument was preheated until reaching the desired reaction temperature (60 ºC), and then the sealed NMR tube was placed inside the instrument and allowed for 15 min grace period in order to reach temperature equilibrium before ^1^H NMR measurements. Product concentrations were determined by measuring terminal CH_3_ proton area relative to the mesitylene internal standard, and data were then linear fitted by the least-square analysis (R^2^ > 0.90) using eq **S1**, where [n-hexylnitrile] is the product concentration at time t. Following the rate law equation **S2**, the reaction orders relative to the hexanamide, La[N(SiMe_3_)_2_]_3_ and PhSiH_3_ could be determined by plotting ln(k_obs_) vs. ln(concentration) correspondingly.

$$\left[ hexylnitrile \right]\left( M \right)=k_{obs}\left( M\cdot s^{-1} \right)\cdot t\left( s \right) S1$$

$$k_{obs}=k\cdot\left[ hexanamide \right]^{a}\cdot\left[ {La}^{NTMS} \right]^{b}\cdot\left[ PhSiH_{3} \right]^{c} S2$$

$${ln(k}_{obs})=\ln\left( k \right)+a\cdot ln\left[ hexanamide \right]+b\cdot ln\left[ {La}^{NTMS} \right]+c\cdot ln\left[ PhSiH_{3} \right]$$

Determination of reaction order relative to hexanamide

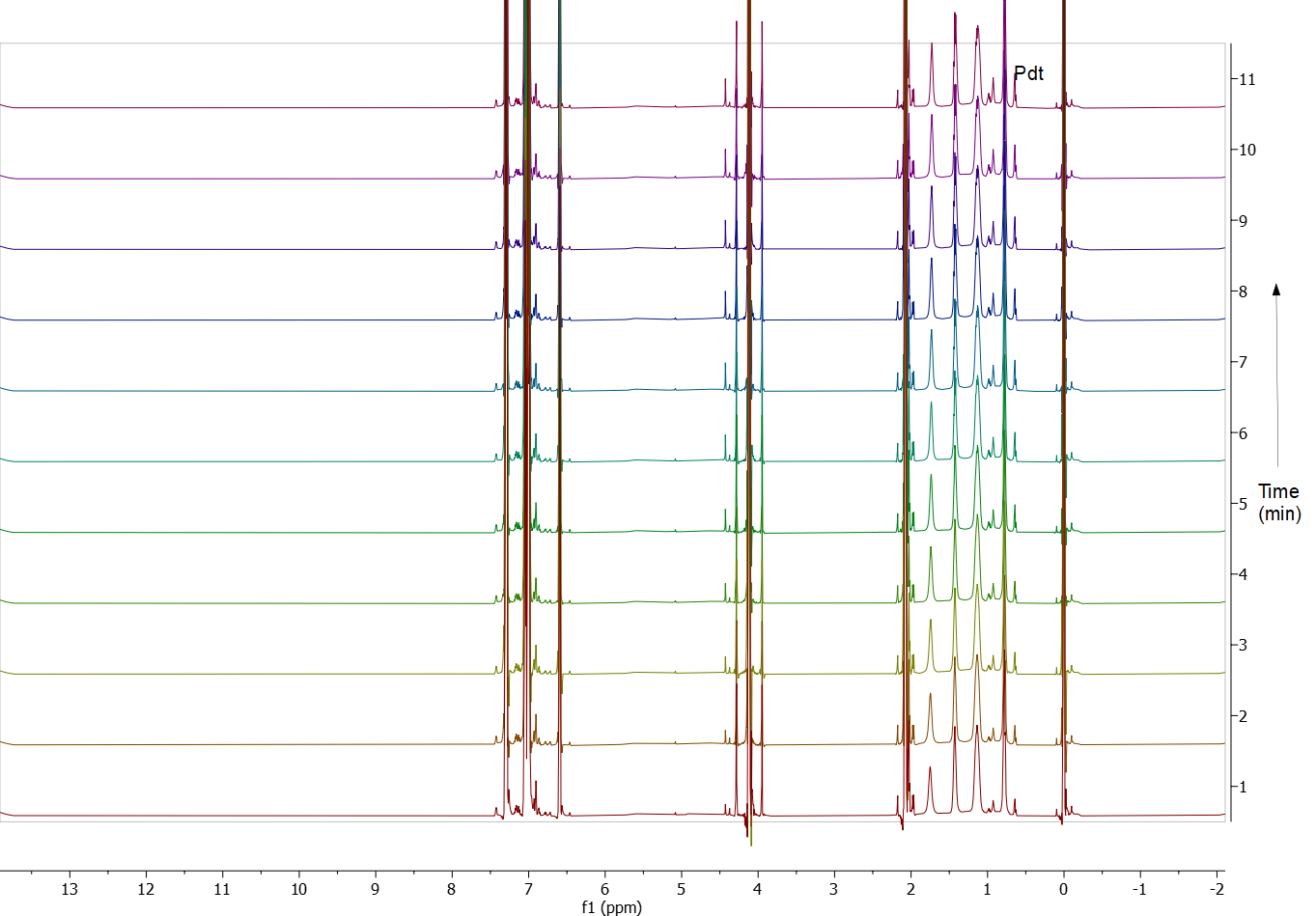


Figure S63. Stacked ^1^H NMR spectra for reaction with 0.15 M substrate.

| Table S1. Raw data of ^1^H NMR spectra for 0.15 M amide | | | | |
| --- | --- | --- | --- | --- |
| Amide | 0.15 M |  |  | k_obs_ (M/s) |
| time (min) | time (sec) | integral | [pdt] (M) | 3.12E-06 |
| 17 | 1020 | 0.03506 | 0.00285871 |  |
| 22 | 1320 | 0.04718 | 0.00384694 |  |
| 27 | 1620 | 0.05234 | 0.00426768 |  |
| 32 | 1920 | 0.07418 | 0.00604846 |  |
| 37 | 2220 | 0.08789 | 0.00716634 |  |
| 42 | 2520 | 0.10065 | 0.00820676 |  |
| 47 | 2820 | 0.11018 | 0.00898381 |  |
| 57 | 3420 | 0.12908 | 0.01052487 |  |
| 62 | 3720 | 0.13796 | 0.01124892 |  |
| 67 | 4020 | 0.15065 | 0.01228363 |  |
| 72 | 4320 | 0.16020 | 0.01306232 |  |

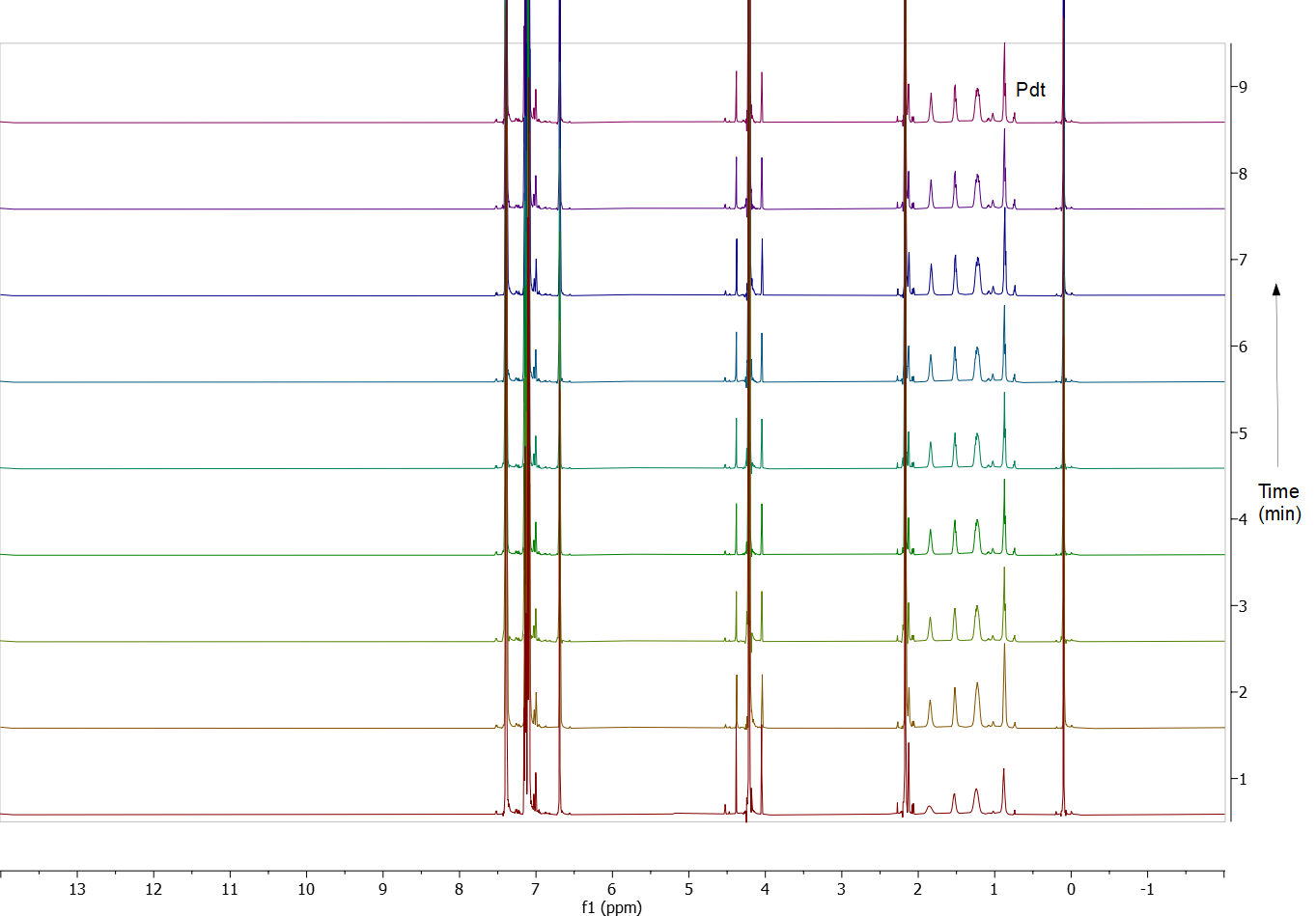


Figure S64. Stacked ^1^H NMR spectra for reaction with 0.17 M substrate.

| Table S2. Raw data of ^1^H NMR spectra for 0.17 M amide | | | | |
| --- | --- | --- | --- | --- |
| Amide | 0.17 M |  |  | k_obs_ (M/s) |
| time (min) | time (sec) | integral | [pdt] (M) | 3.03E-06 |
| 20 | 1200 | 0.03368 | 0.00274619 |  |
| 38 | 2280 | 0.07810 | 0.00636808 |  |
| 46 | 2760 | 0.09920 | 0.00808853 |  |
| 51 | 3060 | 0.11173 | 0.00911019 |  |
| 56 | 3360 | 0.12416 | 0.01012370 |  |
| 61 | 3660 | 0.13012 | 0.01060967 |  |
| 66 | 3960 | 0.13815 | 0.01126441 |  |
| 71 | 4260 | 0.15033 | 0.01225754 |  |
| 76 | 4560 | 0.15998 | 0.01304438 |  |

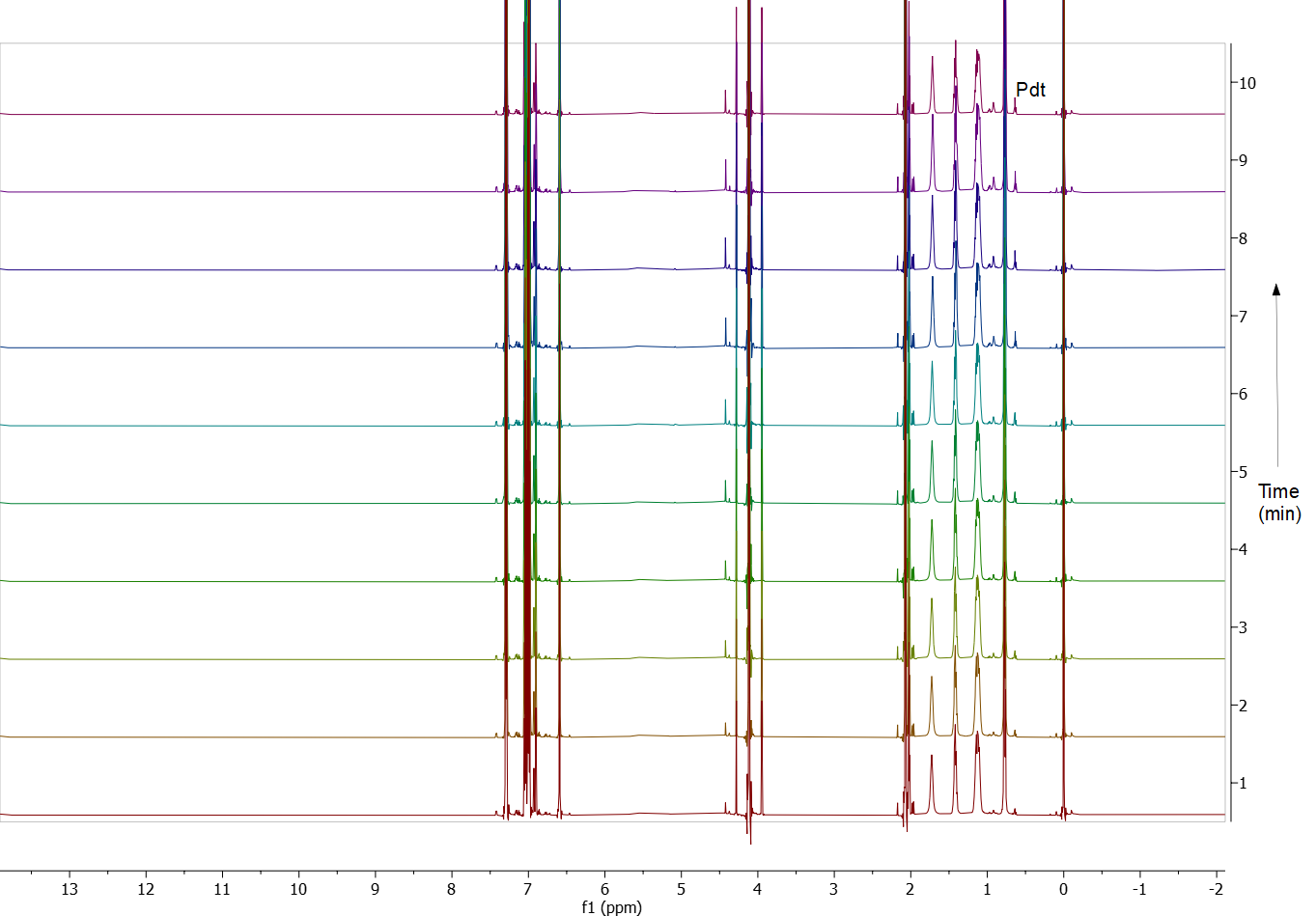


Figure S65. Stacked ^1^H NMR spectra for reaction with 0.22 M substrate.

| Table S3. Raw data of ^1^H NMR spectra for 0.22 M amide | | | | |
| --- | --- | --- | --- | --- |
| Amide | 0.22 M |  |  | k_obs_ (M/s) |
| time (min) | time (sec) | integral | [pdt] (M) | 3.12E-06 |
| 25 | 1500 | 0.03749 | 0.00305684 |  |
| 30 | 1800 | 0.04715 | 0.00384450 |  |
| 38 | 2280 | 0.06051 | 0.00493384 |  |
| 43 | 2580 | 0.06847 | 0.00558288 |  |
| 48 | 2880 | 0.07944 | 0.00647734 |  |
| 53 | 3180 | 0.09290 | 0.00757484 |  |
| 64 | 3840 | 0.12174 | 0.00992638 |  |
| 69 | 4140 | 0.13401 | 0.01092685 |  |
| 74 | 4440 | 0.14984 | 0.01221759 |  |
| 79 | 4740 | 0.15683 | 0.01278754 |  |

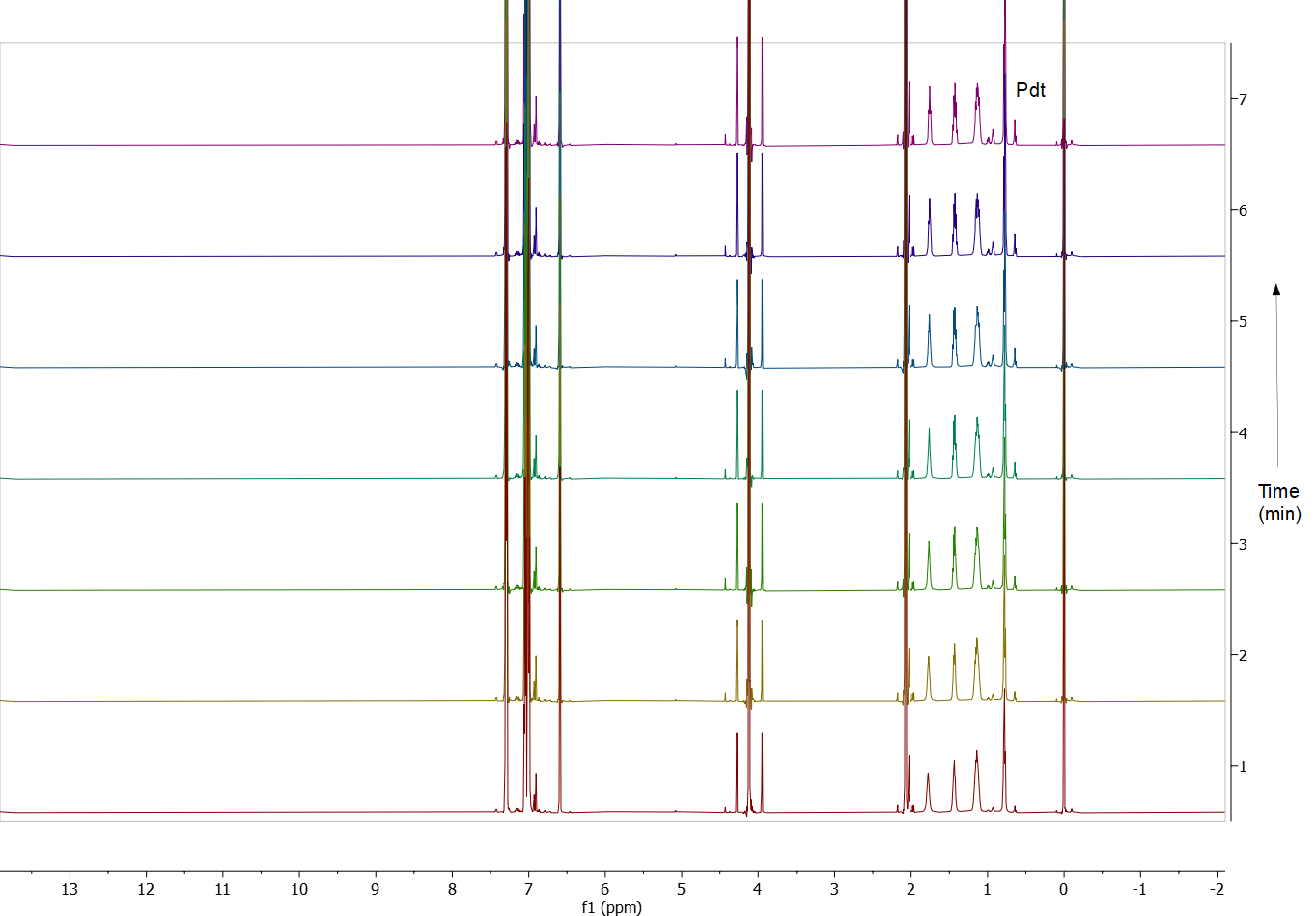


Figure S66. Stacked ^1^H NMR spectra for reaction with 0.26 M substrate.

| Table S4. Raw data of ^1^H NMR spectra for 0.26 M amide | | | | |
| --- | --- | --- | --- | --- |
| Amide | 0.26 M |  |  | k_obs_ (M/s) |
| time (min) | time (sec) | integral | [pdt] (M) | 3.27E-06 |
| 23 | 1380 | 0.06466 | 0.00527222 |  |
| 33 | 1980 | 0.09538 | 0.00777705 |  |
| 43 | 2580 | 0.12722 | 0.01037321 |  |
| 53 | 3180 | 0.15153 | 0.01235539 |  |
| 63 | 3780 | 0.16783 | 0.01368445 |  |
| 73 | 4380 | 0.19930 | 0.01625044 |  |
| 83 | 4980 | 0.20673 | 0.01685626 |  |

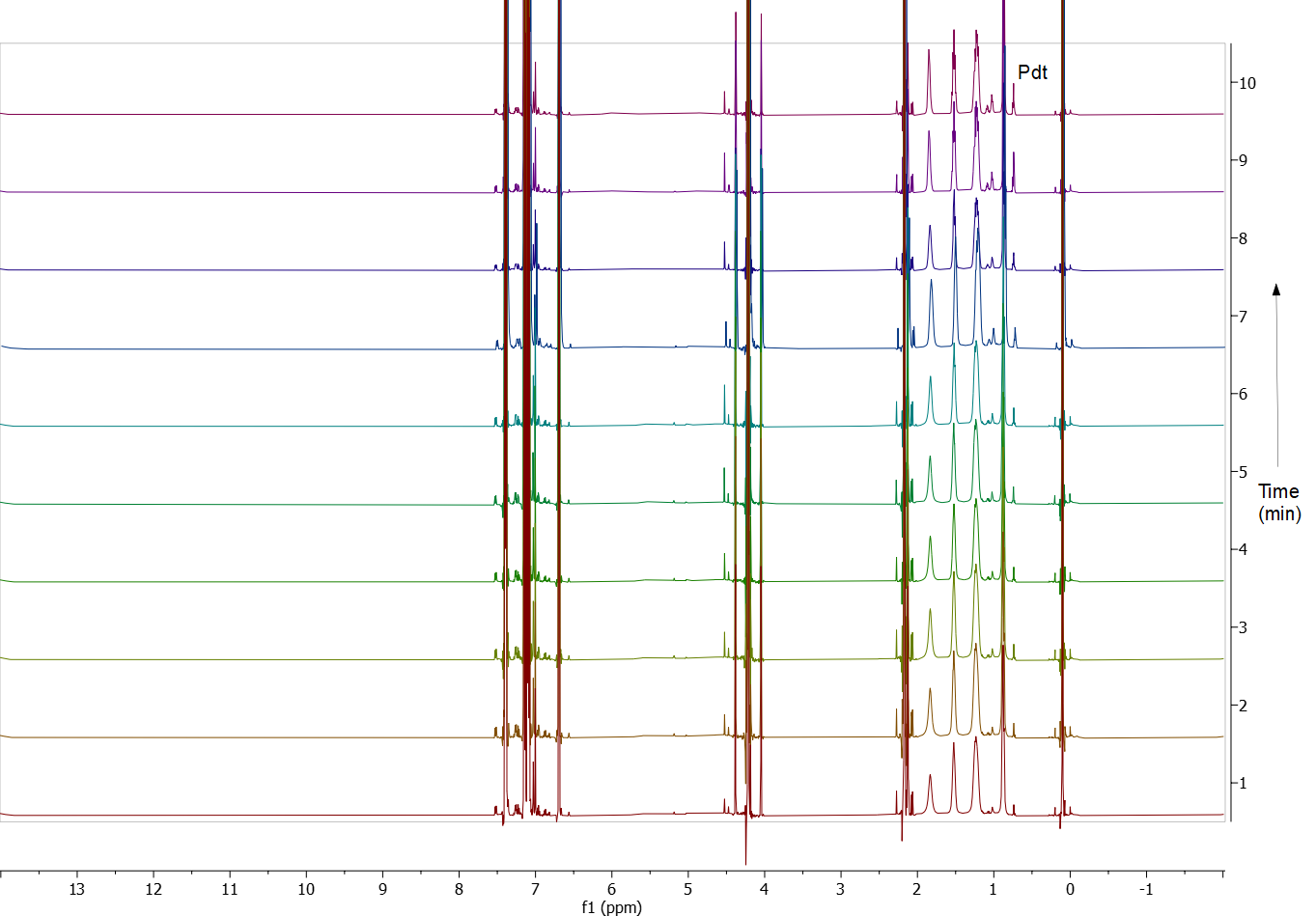


Figure S67. Stacked ^1^H NMR spectra for reaction with 0.35 M substrate.

| Table S5. Raw data of ^1^H NMR spectra for 0.35 M amide | | | | |
| --- | --- | --- | --- | --- |
| Amide | 0.35 M |  |  | k_obs_ (M/s) |
| time (min) | time (sec) | integral | [pdt] (M) | 3.39E-06 |
| 14 | 840 | 0.02613 | 0.00213058 |  |
| 19 | 1140 | 0.03668 | 0.00299080 |  |
| 24 | 1440 | 0.03999 | 0.00326069 |  |
| 30 | 1800 | 0.05124 | 0.00417798 |  |
| 35 | 2100 | 0.06080 | 0.00495748 |  |
| 40 | 2400 | 0.06647 | 0.00541980 |  |
| 54 | 3240 | 0.11823 | 0.00964019 |  |
| 60 | 3600 | 0.13026 | 0.01062108 |  |
| 75 | 4500 | 0.17413 | 0.01419814 |  |
| 80 | 4800 | 0.18300 | 0.01492137 |  |

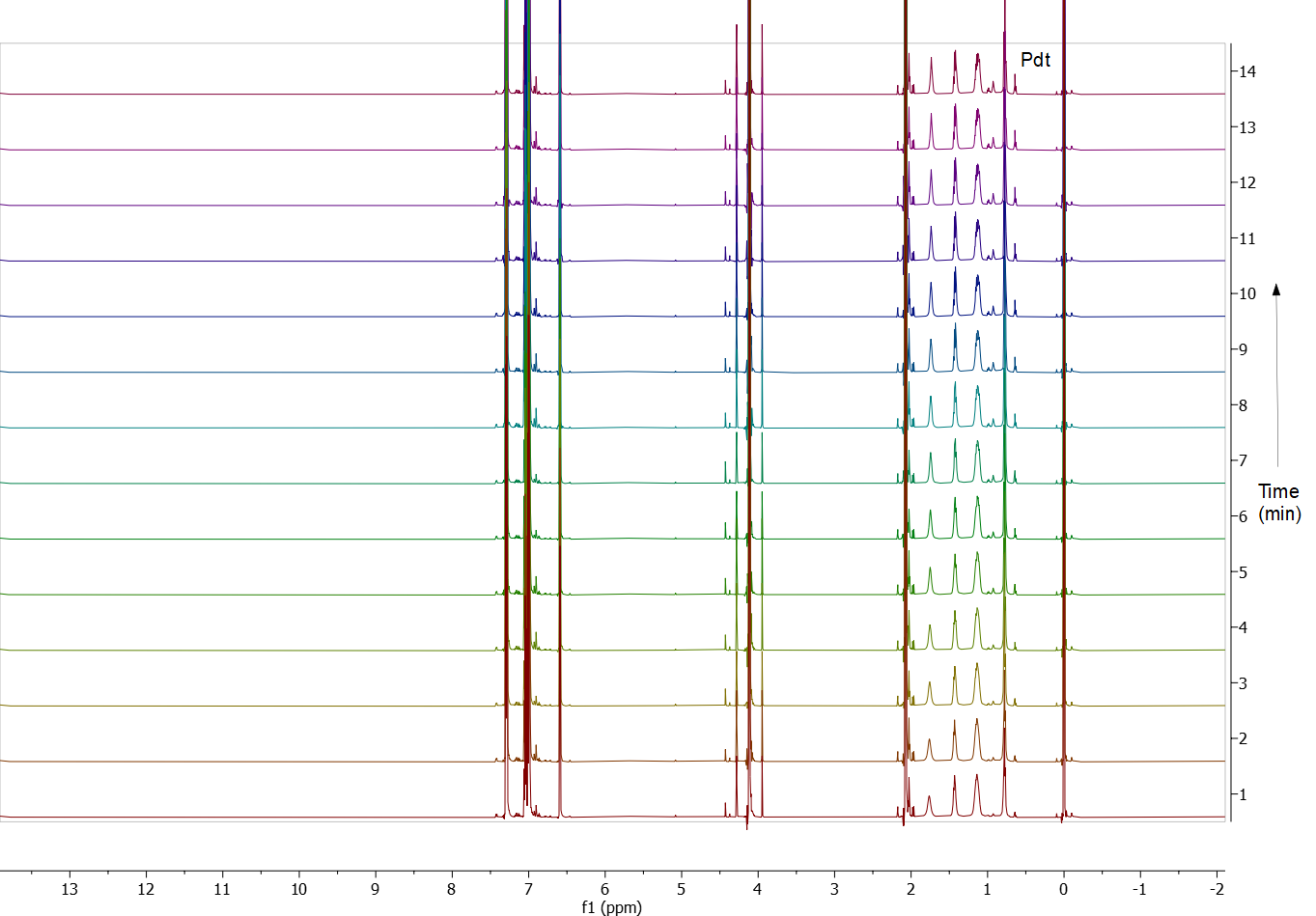


Figure S68. Stacked ^1^H NMR spectra for reaction with 0.45 M substrate.

| Table S6. Raw data of ^1^H NMR spectra for 0.45 M amide | | | | |
| --- | --- | --- | --- | --- |
| Amide | 0.45 M |  |  | k_obs_ (M/s) |
| time (min) | time (sec) | integral | [pdt] (M) | 3.39E-06 |
| 15 | 900 | 0.0209 | 0.00170414 |  |
| 20 | 1200 | 0.03621 | 0.00295248 |  |
| 25 | 1500 | 0.05317 | 0.00433535 |  |
| 30 | 1800 | 0.06003 | 0.00489470 |  |
| 35 | 2100 | 0.07212 | 0.00588049 |  |
| 40 | 2400 | 0.08784 | 0.00716226 |  |
| 45 | 2700 | 0.10038 | 0.00818474 |  |
| 50 | 3000 | 0.11597 | 0.00945591 |  |
| 55 | 3300 | 0.12893 | 0.01051264 |  |
| 60 | 3600 | 0.14226 | 0.01159953 |  |
| 65 | 3900 | 0.15205 | 0.01239779 |  |
| 70 | 4200 | 0.16267 | 0.01326372 |  |
| 80 | 4800 | 0.18129 | 0.01478195 |  |

| Table S7. Data of ln(k_obs_) vs. ln[Amide] | |
| --- | --- |
| ln[Amide] | ln(k_obs_) |
| -1.89712 | -12.677678 |
| -1.7719568 | -12.706948 |
| -1.5278579 | -12.677678 |
| -1.3470736 | -12.630721 |
| -1.0498221 | -12.594681 |
| -0.7985077 | -12.594681 |

Determination of reaction order relative to La^NTMS^

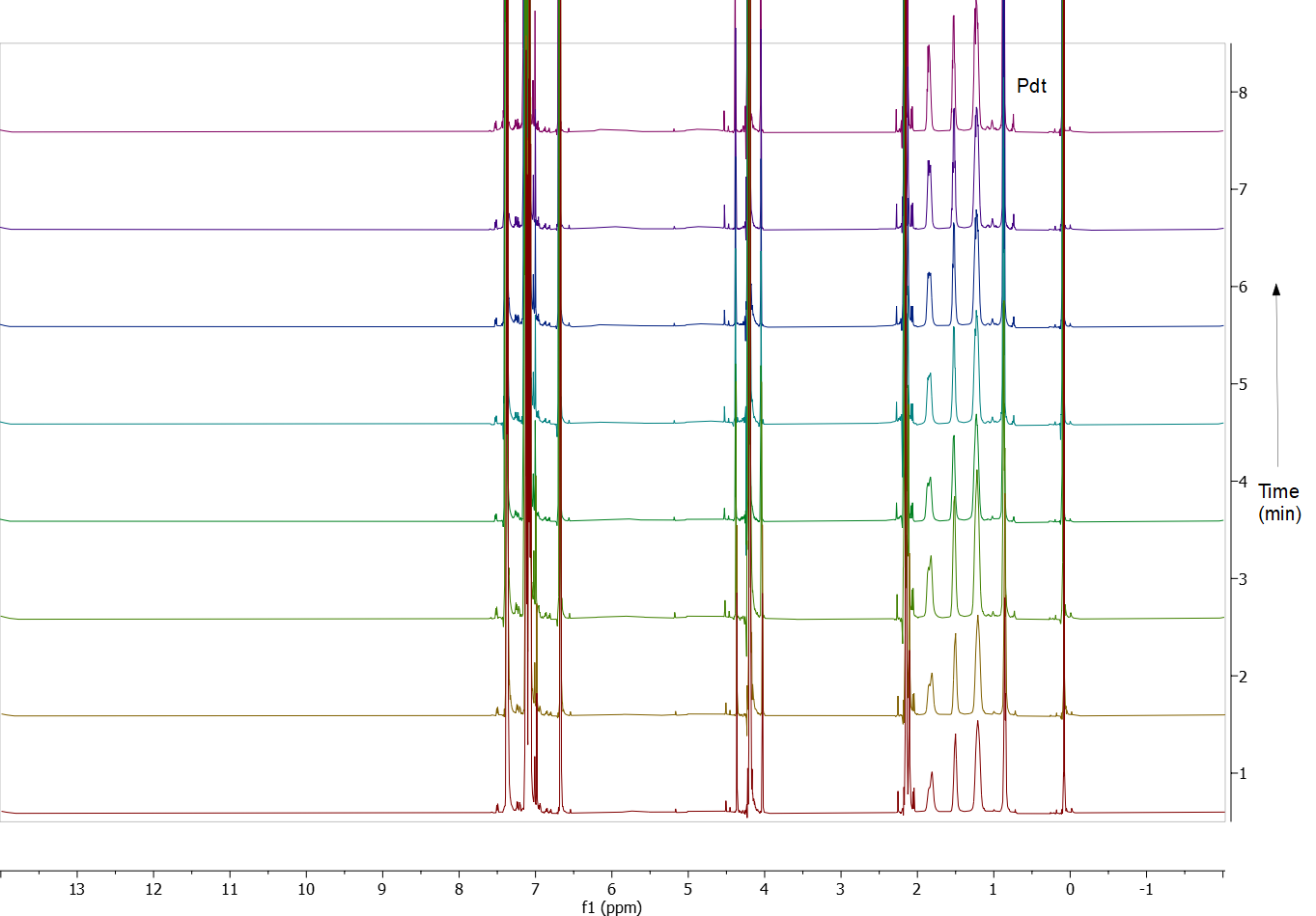


Figure S69. Stacked ^1^H NMR spectra for reaction with 2.5 mol% La^NTMS^.

| Table S8. Raw data of ^1^H NMR spectra for 2.5 mol% La^NTMS^ | | | | |
| --- | --- | --- | --- | --- |
| 2.5 mol% [La] | 6.5 mM |  |  | k_obs_ (M/s) |
| time (min) | time (sec) | integral | [pdt] (M) | 1.50E-06 |
| 15 | 900 | 0.01151 | 0.000938497 |  |
| 25 | 1500 | 0.01486 | 0.001211648 |  |
| 30 | 1800 | 0.02037 | 0.001660920 |  |
| 35 | 2100 | 0.02532 | 0.002064531 |  |
| 50 | 3000 | 0.04101 | 0.003343856 |  |
| 55 | 3300 | 0.05001 | 0.004077694 |  |
| 65 | 3900 | 0.06283 | 0.005123005 |  |
| 70 | 4200 | 0.06852 | 0.005586954 |  |

(Same data for Table S4.)

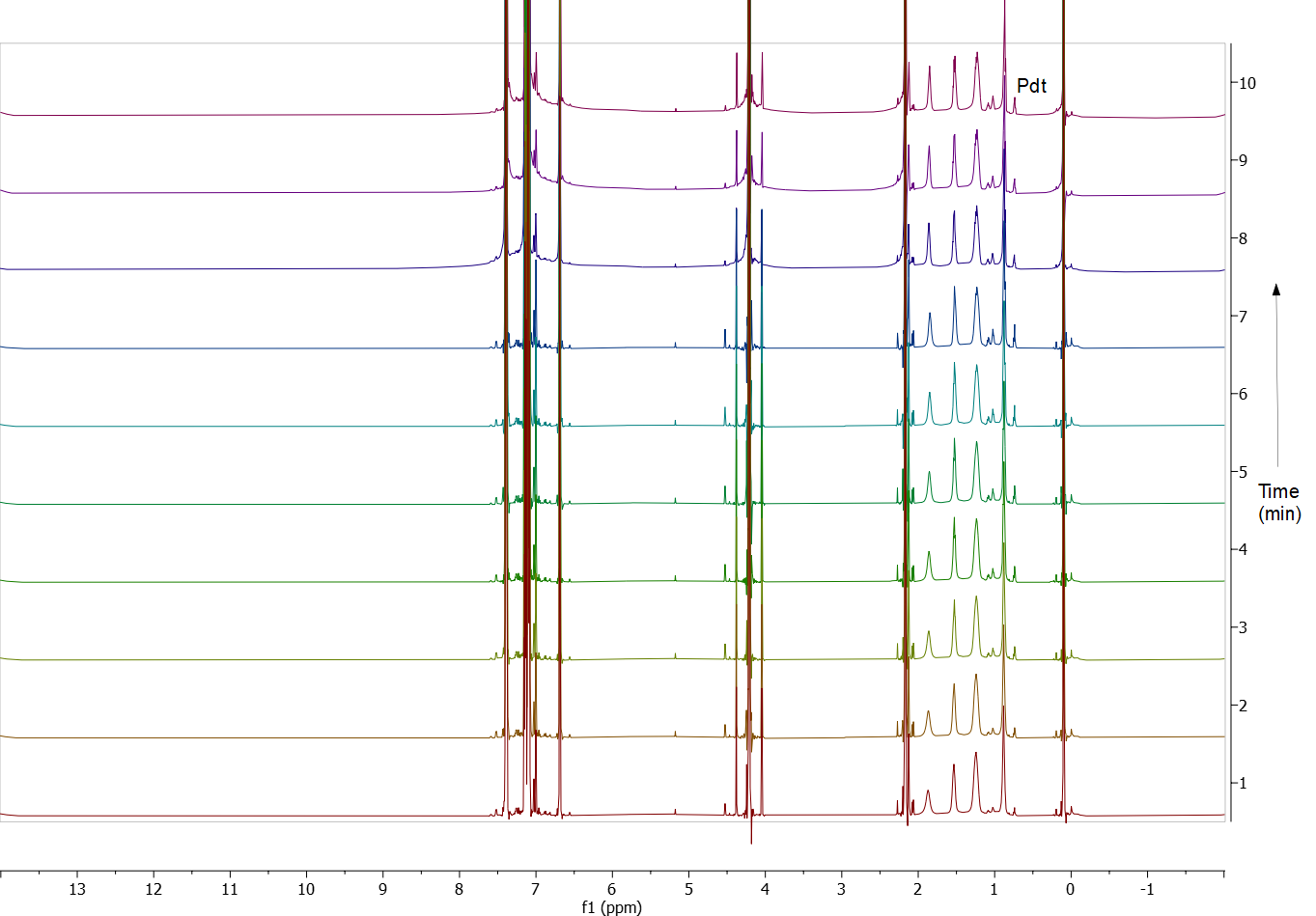


Figure S70. Stacked ^1^H NMR spectra for reaction with 7.5 mol% La^NTMS^.

| Table S9. Raw data of ^1^H NMR spectra for 7.5 mol% La^NTMS^ | | | | |
| --- | --- | --- | --- | --- |
| 7.5 mol% [La] | 19.5 mM |  |  | k_obs_ (M/s) |
| time (min) | time (sec) | integral | [pdt] (M) | 5.26E-06 |
| 13 | 780 | 0.04990 | 0.004068725 |  |
| 18 | 1080 | 0.06519 | 0.005315434 |  |
| 23 | 1380 | 0.08299 | 0.006766803 |  |
| 28 | 1680 | 0.09916 | 0.008085265 |  |
| 33 | 1980 | 0.11904 | 0.009706232 |  |
| 38 | 2280 | 0.13370 | 0.010901573 |  |
| 43 | 2580 | 0.15535 | 0.012666861 |  |
| 52 | 3120 | 0.20053 | 0.016350728 |  |
| 57 | 3420 | 0.21942 | 0.017890973 |  |
| 67 | 4020 | 0.25306 | 0.020633896 |  |


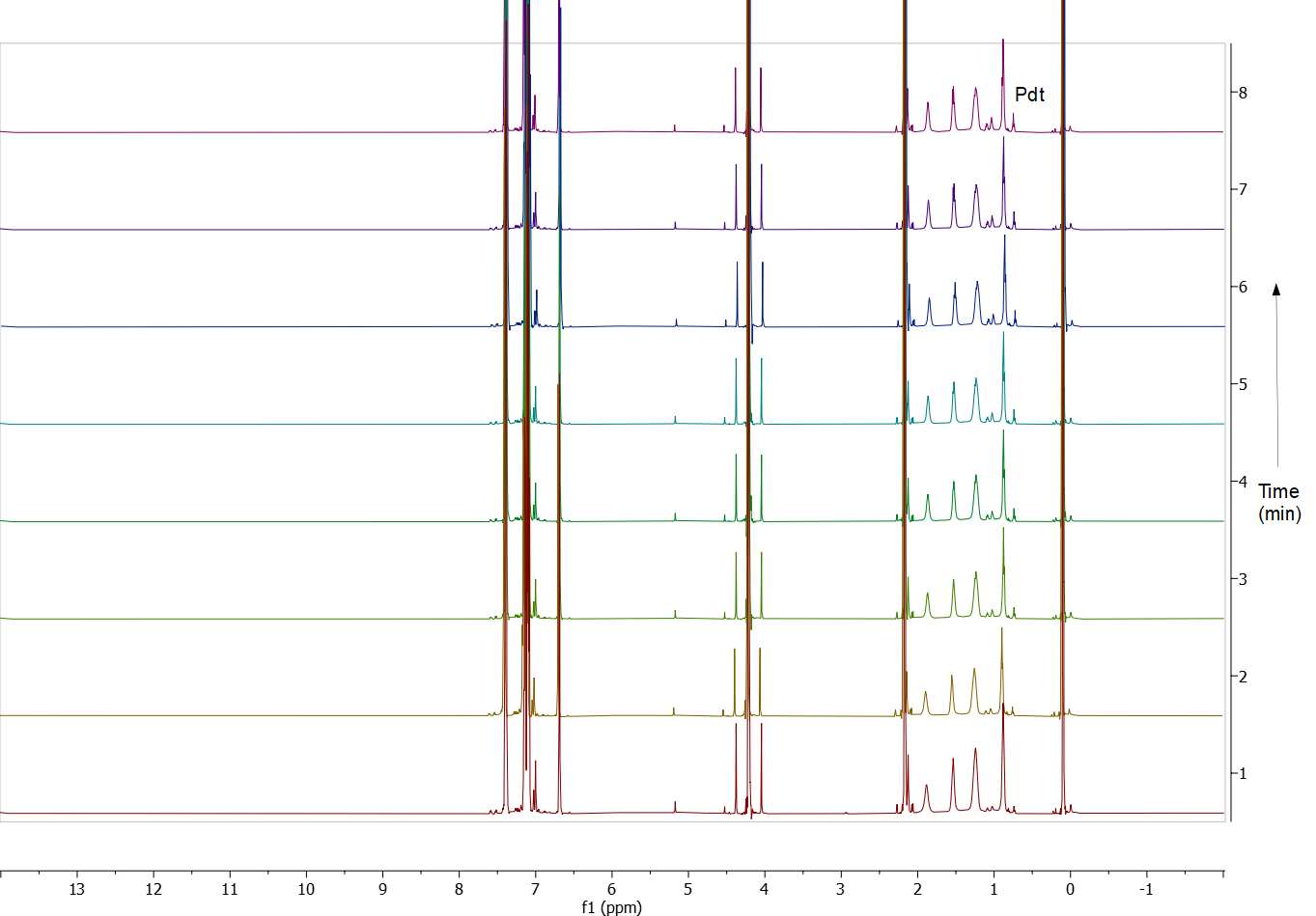


Figure S71. Stacked ^1^H NMR spectra for reaction with 10 mol% La^NTMS^.

| Table S10. Raw data of ^1^H NMR spectra for 10 mol% La^NTMS^ | | | | |
| --- | --- | --- | --- | --- |
| 10 mol% [La] | 26 mM |  |  | k_obs_ (M/s) |
| time (min) | time (sec) | integral | [pdt] (M) | 6.26E-06 |
| 15 | 900 | 0.08391 | 0.006841817 |  |
| 25 | 1500 | 0.13473 | 0.010985556 |  |
| 32 | 1920 | 0.17376 | 0.014167967 |  |
| 37 | 2220 | 0.18579 | 0.015148864 |  |
| 42 | 2520 | 0.21519 | 0.017546069 |  |
| 47 | 2820 | 0.24040 | 0.019601631 |  |
| 52 | 3120 | 0.26199 | 0.021362027 |  |
| 57 | 3420 | 0.27364 | 0.022311939 |  |

| Table S11. Data of ln(k_obs_) vs. ln[La^NTMS^] | |
| --- | --- |
| ln[La^NTMS^] | ln(k_obs_) |
| -5.0359531 | -13.410045 |
| -4.3428059 | -12.630721 |
| -3.9358035 | -12.15538 |
| -3.6496587 | -11.98133 |

Determination of reaction order relative to PhSiH_3_ (limiting condition)


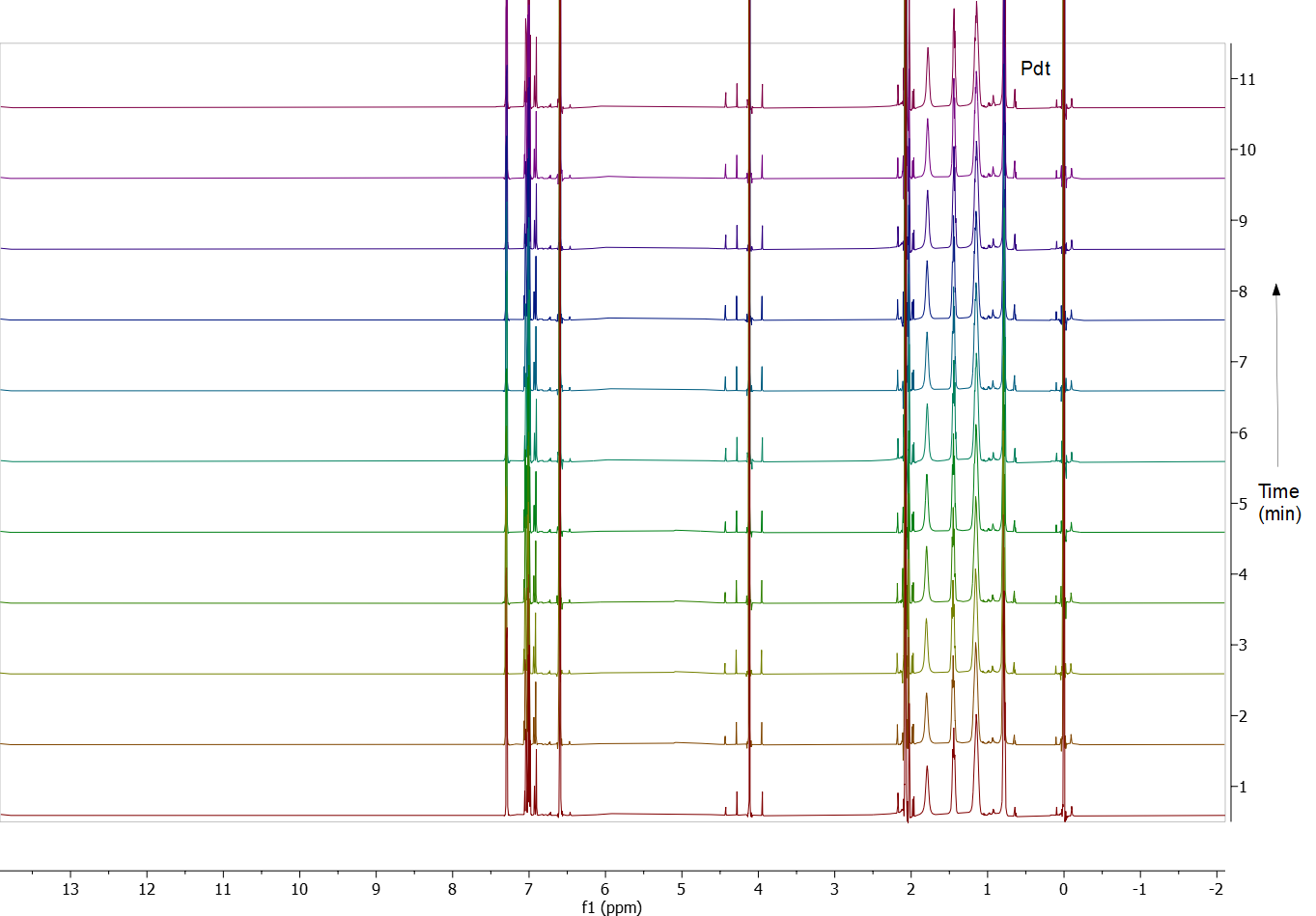


Figure S72. Stacked ^1^H NMR spectra for reaction with 0.5 eq PhSiH_3_.

| Table S12. Raw data of ^1^H NMR spectra for 0.5 eq PhSiH_3_ | | | | |
| --- | --- | --- | --- | --- |
| 0.5 eq PhSiH3 | 0.13 M |  |  | k_obs_ (M/s) |
| time (min) | time (sec) | integral | [pdt] (M) | 7.56E-07 |
| 14 | 840 | 0.03773 | 0.00307641 |  |
| 19 | 1140 | 0.04214 | 0.00343599 |  |
| 29 | 1740 | 0.04598 | 0.00374910 |  |
| 34 | 2040 | 0.05088 | 0.00414863 |  |
| 39 | 2340 | 0.05398 | 0.00440140 |  |
| 44 | 2640 | 0.05515 | 0.00449680 |  |
| 52 | 3120 | 0.06084 | 0.00496075 |  |
| 57 | 3420 | 0.06269 | 0.00511159 |  |
| 62 | 3720 | 0.06581 | 0.00536599 |  |
| 72 | 4320 | 0.07218 | 0.00588538 |  |
| 82 | 4920 | 0.07502 | 0.00611695 |  |


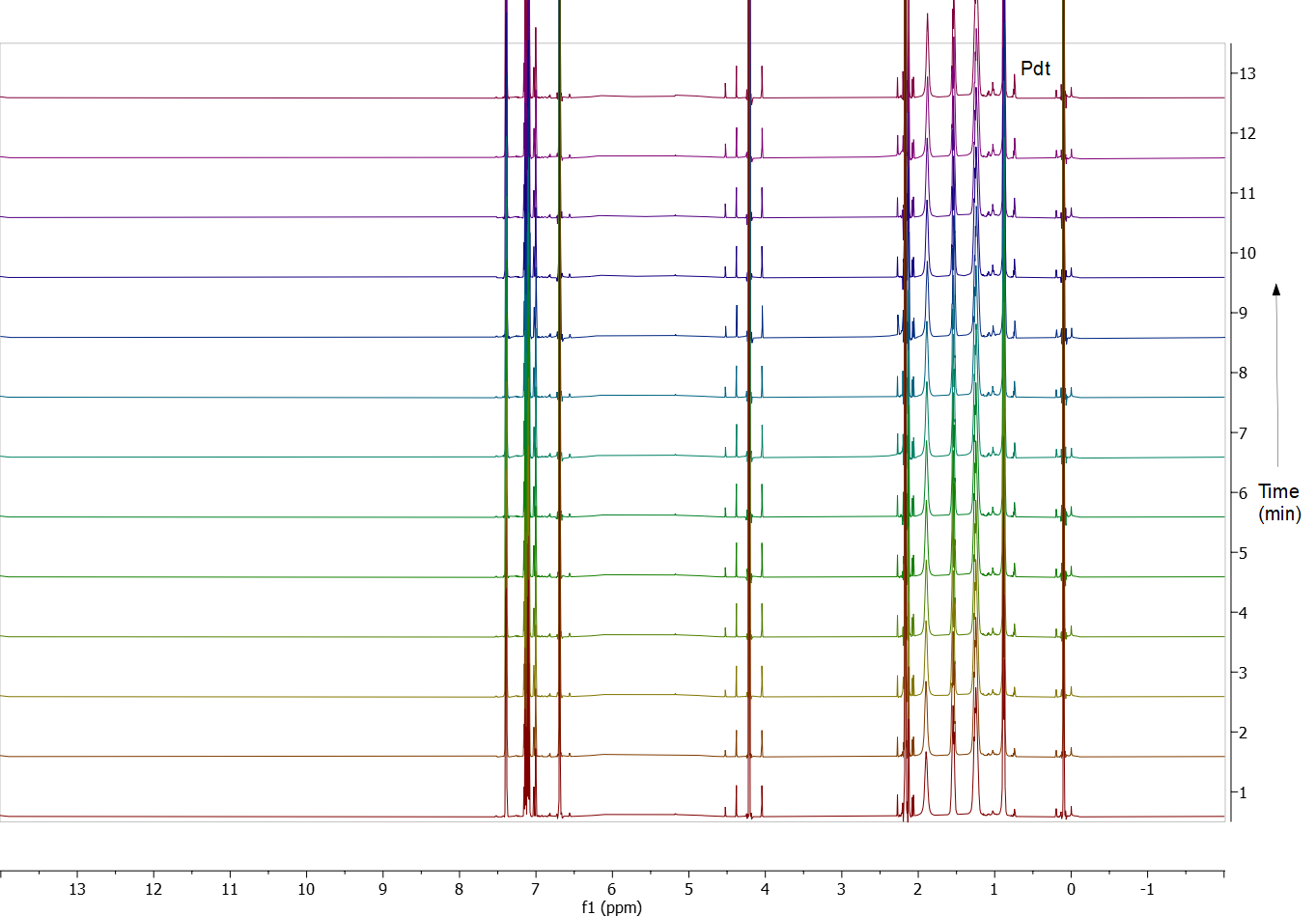


Figure S73. Stacked ^1^H NMR spectra for reaction with 0.75 eq PhSiH_3_.

| Table S13. Raw data of ^1^H NMR spectra for 0.75 eq PhSiH_3_ | | | | |
| --- | --- | --- | --- | --- |
| 0.75 eq PhSiH3 | 0.195 M |  |  | k_obs_ (M/s) |
| time (min) | time (sec) | integral | [pdt] (M) | 1.23E-06 |
| 17 | 1020 | 0.03771 | 0.00307478 |  |
| 22 | 1320 | 0.04303 | 0.00350856 |  |
| 27 | 1620 | 0.04957 | 0.00404182 |  |
| 32 | 1920 | 0.05479 | 0.00446744 |  |
| 37 | 2220 | 0.05932 | 0.00483681 |  |
| 42 | 2520 | 0.06344 | 0.00517274 |  |
| 47 | 2820 | 0.06580 | 0.00536517 |  |
| 52 | 3120 | 0.06923 | 0.00564485 |  |
| 57 | 3420 | 0.07207 | 0.00587641 |  |
| 62 | 3720 | 0.07844 | 0.00639581 |  |
| 67 | 4020 | 0.08404 | 0.00685242 |  |
| 72 | 4320 | 0.08945 | 0.00729354 |  |
| 77 | 4620 | 0.09624 | 0.00784718 |  |


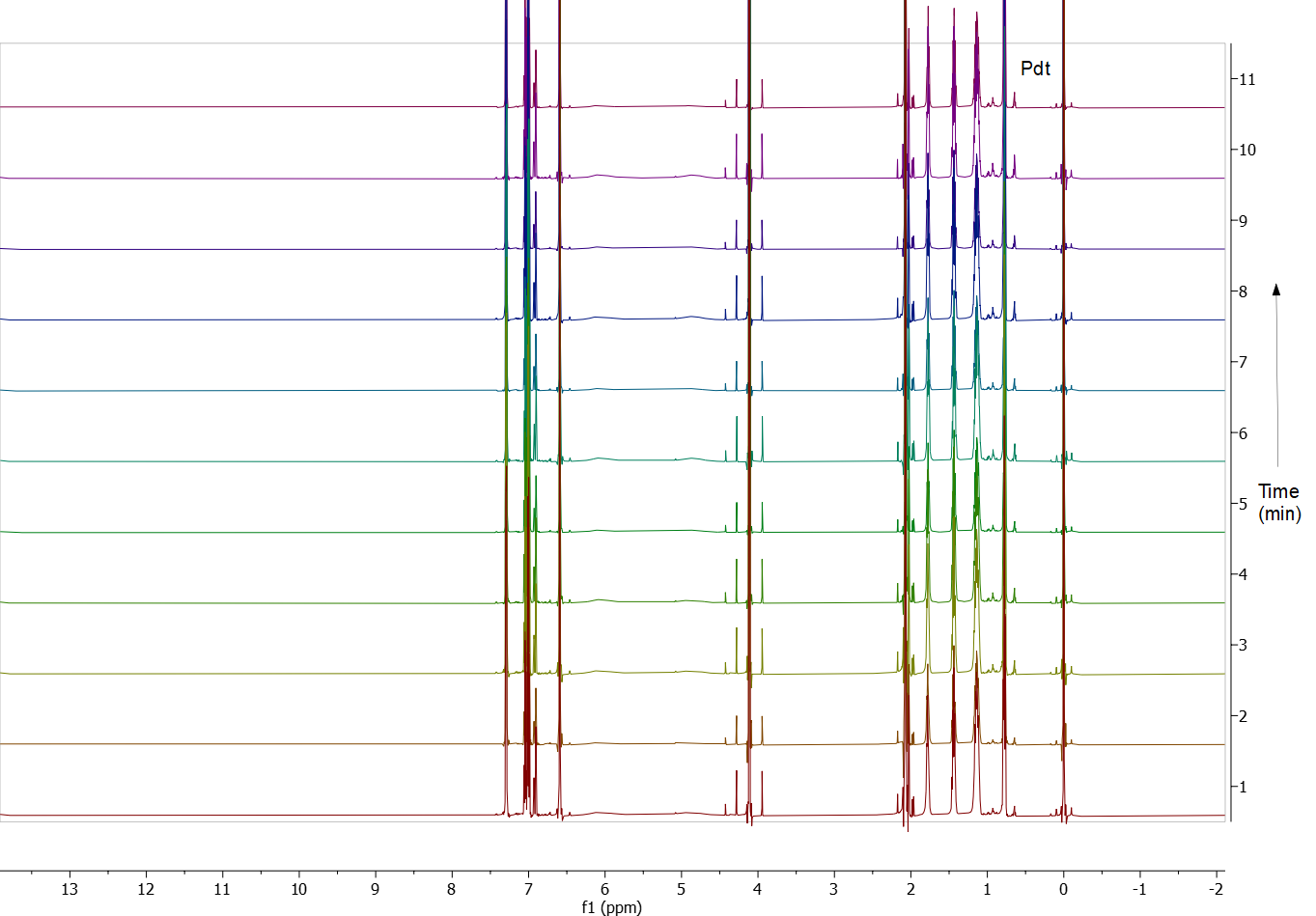


Figure S74. Stacked ^1^H NMR spectra for reaction with 1 eq PhSiH_3_.

| Table S14. Raw data of ^1^H NMR spectra for 1 eq PhSiH_3_ | | | | |
| --- | --- | --- | --- | --- |
| 1 eq PhSiH3 | 0.26 M |  |  | k_obs_ (M/s) |
| time (min) | time (sec) | integral | [pdt] (M) | 1.59E-06 |
| 18 | 1080 | 0.04239 | 0.00345638 |  |
| 23 | 1380 | 0.04974 | 0.00405568 |  |
| 32 | 1920 | 0.05939 | 0.00484252 |  |
| 37 | 2220 | 0.06672 | 0.00544019 |  |
| 42 | 2520 | 0.07220 | 0.00588701 |  |
| 47 | 2820 | 0.07752 | 0.00632079 |  |
| 52 | 3120 | 0.08321 | 0.00678474 |  |
| 58 | 3480 | 0.09005 | 0.00734246 |  |
| 64 | 3840 | 0.09717 | 0.00792301 |  |
| 76 | 4560 | 0.11077 | 0.00903192 |  |
| 86 | 5160 | 0.11144 | 0.00908655 |  |


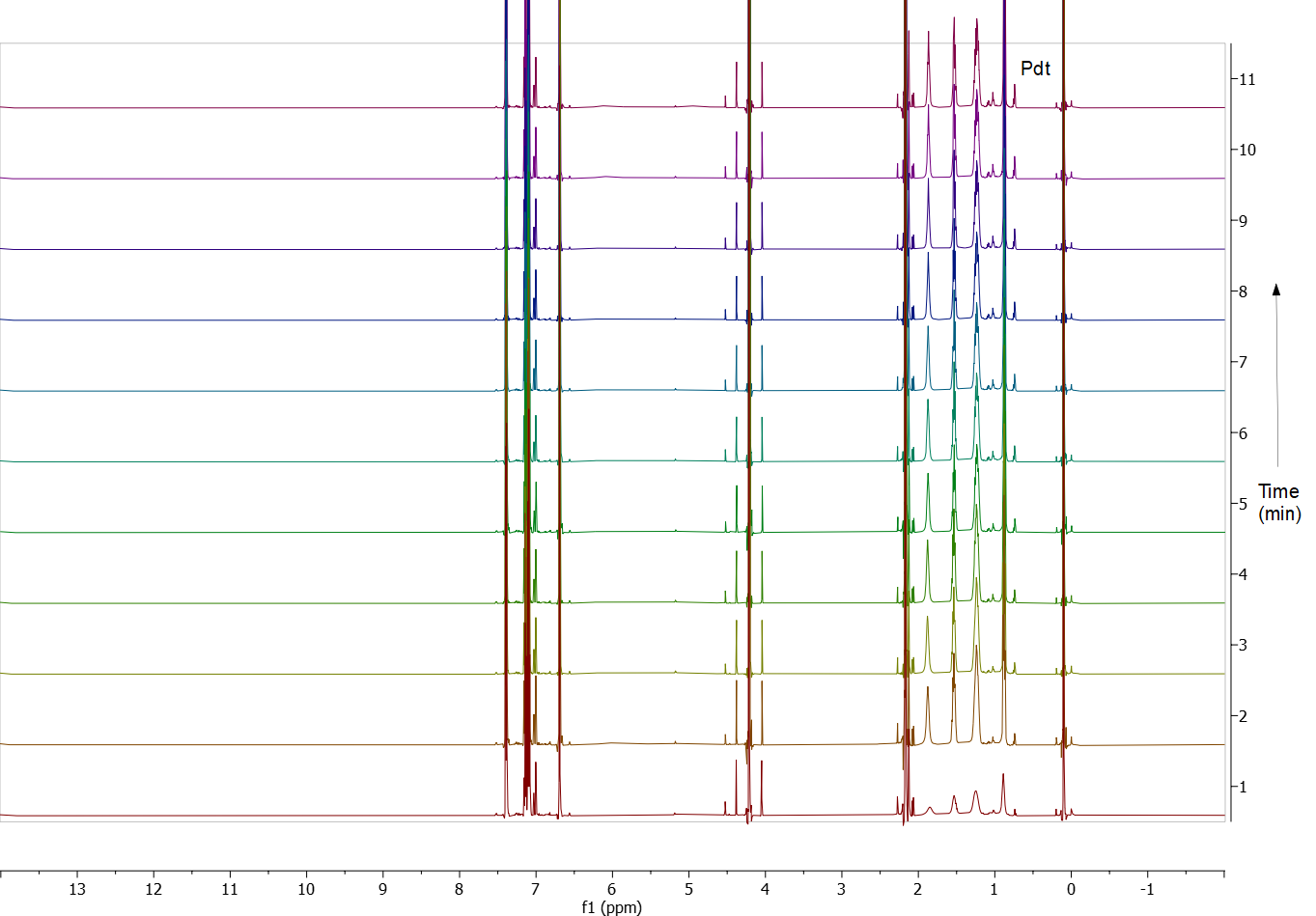


Figure S75. Stacked ^1^H NMR spectra for reaction with 1.5 eq PhSiH_3_.

| Table S15. Raw data of ^1^H NMR spectra for 1.5 eq PhSiH_3_ | | | | |
| --- | --- | --- | --- | --- |
| 1.5 eq PhSiH3 | 0.39 M |  |  | k_obs_ (M/s) |
| time (min) | time (sec) | integral | [pdt] (M) | 2.83E-06 |
| 18 | 1080 | 0.02995 | 0.00244205 |  |
| 30 | 1800 | 0.05278 | 0.00430355 |  |
| 36 | 2160 | 0.06705 | 0.00546709 |  |
| 41 | 2460 | 0.07721 | 0.00629552 |  |
| 46 | 2760 | 0.08213 | 0.00669668 |  |
| 51 | 3060 | 0.09339 | 0.00761479 |  |
| 56 | 3360 | 0.10960 | 0.00893652 |  |
| 61 | 3660 | 0.11968 | 0.00975842 |  |
| 66 | 3960 | 0.12981 | 0.01058439 |  |
| 71 | 4260 | 0.13667 | 0.01114374 |  |
| 76 | 4560 | 0.15040 | 0.01226325 |  |


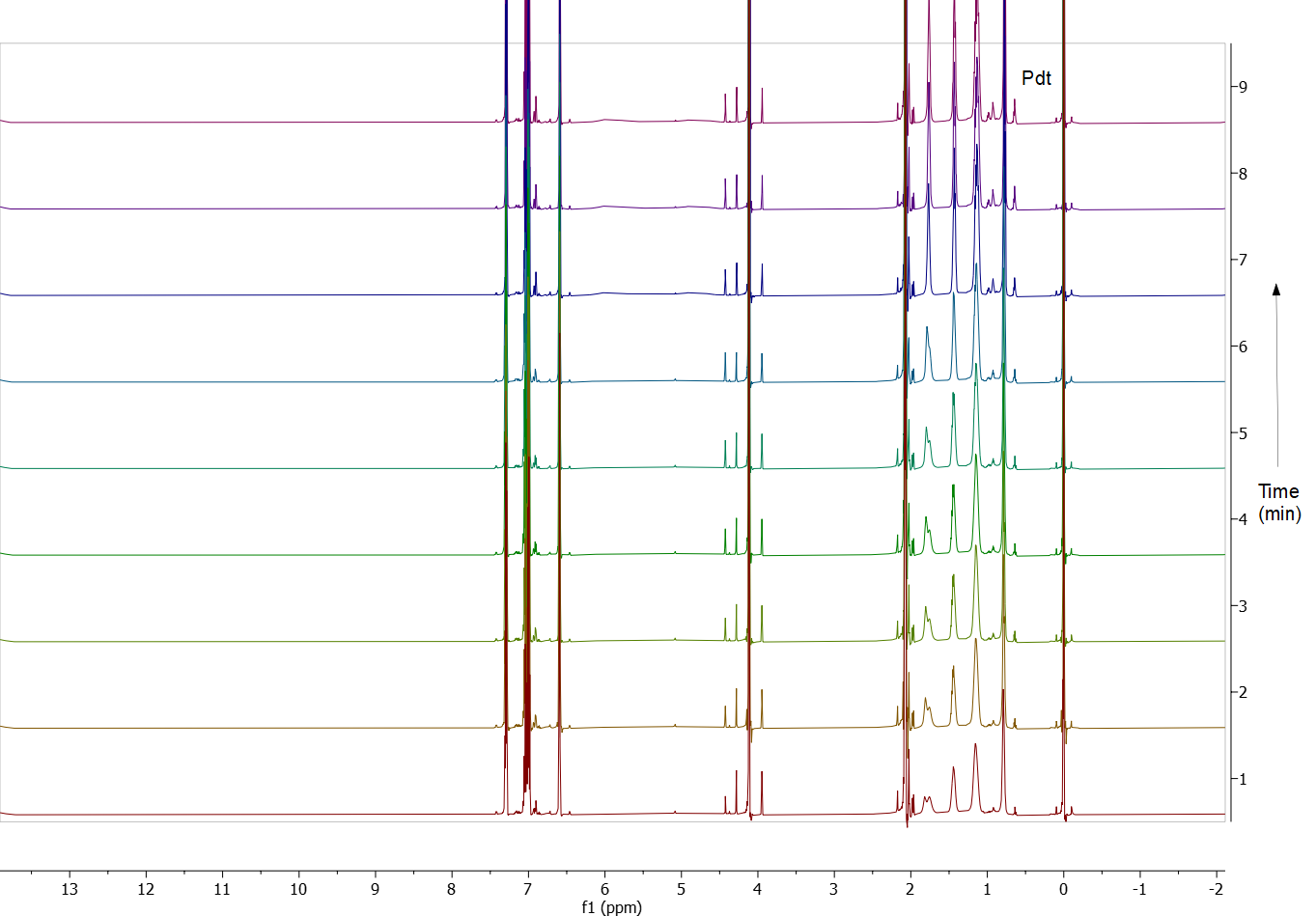


Figure S76. Stacked ^1^H NMR spectra for reaction with 2 eq PhSiH_3_.

| Table S16. Raw data of ^1^H NMR spectra for 2 eq PhSiH_3_ | | | | |
| --- | --- | --- | --- | --- |
| 2 eq PhSiH3 | 0.52 M |  |  | k_obs_ (M/s) |
| time (min) | time (sec) | integral | [pdt] (M) | 3.43E-06 |
| 15 | 900 | 0.03075 | 0.00250728 |  |
| 25 | 1500 | 0.03799 | 0.00309761 |  |
| 30 | 1800 | 0.04428 | 0.00361048 |  |
| 35 | 2100 | 0.06036 | 0.00492161 |  |
| 40 | 2400 | 0.0687 | 0.00560163 |  |
| 50 | 3000 | 0.0953 | 0.00777053 |  |
| 60 | 3600 | 0.12575 | 0.01025335 |  |
| 70 | 4200 | 0.14805 | 0.01207164 |  |
| 75 | 4500 | 0.15965 | 0.01301747 |  |

| Table S17. Data of ln(k_obs_) vs. ln[PhSiH_3_] | |
| --- | --- |
| ln[Ph-SiH_3_] | ln(k_obs_) |
| -1.3470736 | -13.351777 |
| -2.0402208 | -14.095224 |
| -0.9390477 | -12.775234 |
| -1.6347557 | -13.608496 |
| -0.6539265 | -12.58295 |

Determination of reaction order relative to PhSiH_3_ (excess condition)


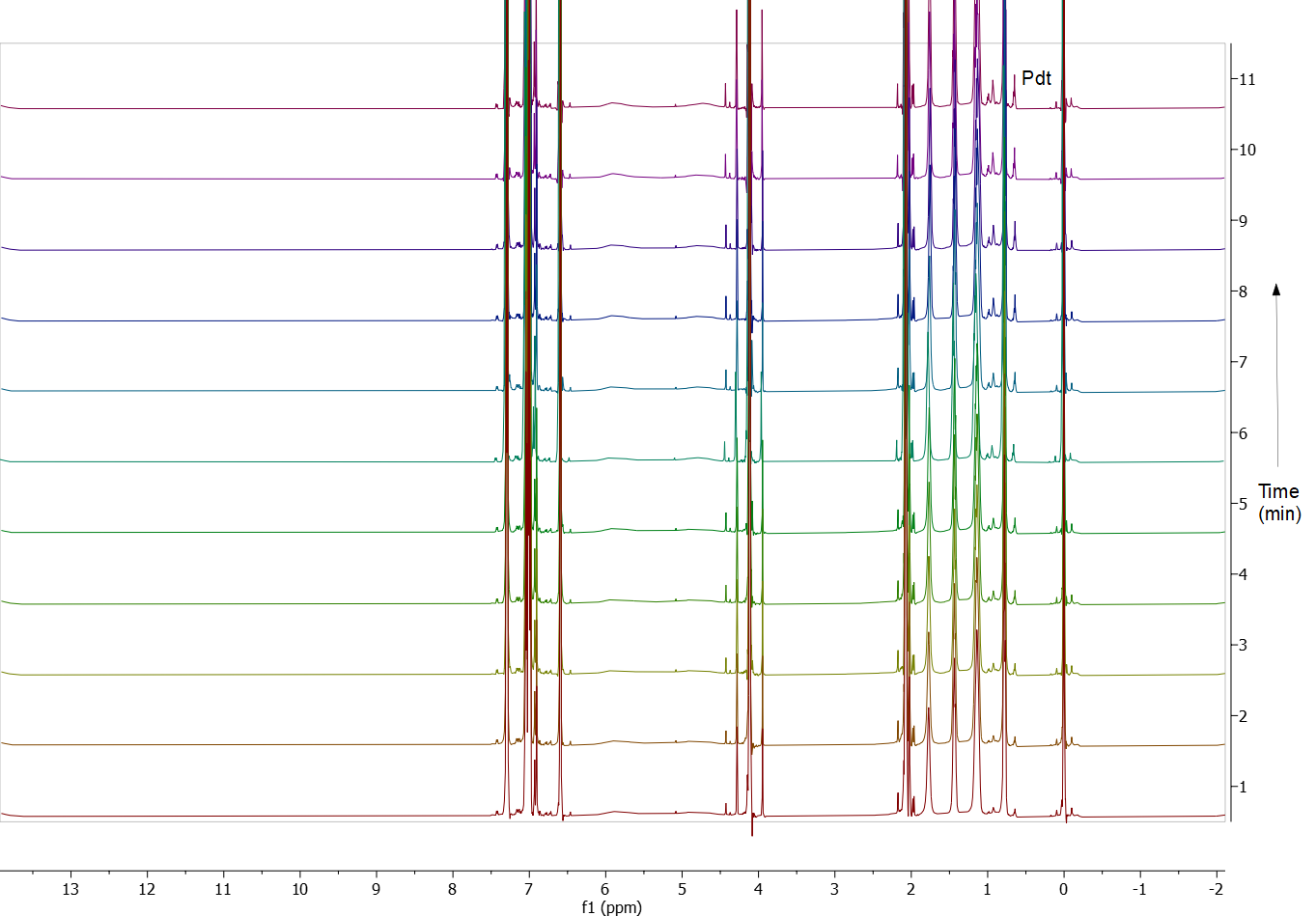


Figure S77. Stacked ^1^H NMR spectra for reaction with 2.5 eq PhSiH_3_.

| Table S18. Raw data of ^1^H NMR spectra for 2.5 eq PhSiH_3_ | | | | |
| --- | --- | --- | --- | --- |
| 2.5 eq PhSiH3 | 0.525 M |  |  | k_obs_ (M/s) |
| time (min) | time (sec) | integral | [pdt] (M) | 2.18E-06 |
| 17 | 1020 | 0.03750 | 0.00246387 |  |
| 22 | 1320 | 0.04410 | 0.00289751 |  |
| 27 | 1620 | 0.05017 | 0.00329633 |  |
| 32 | 1920 | 0.06510 | 0.00427727 |  |
| 37 | 2220 | 0.07406 | 0.00486597 |  |
| 42 | 2520 | 0.08235 | 0.00541065 |  |
| 47 | 2820 | 0.09084 | 0.00596847 |  |
| 58 | 3480 | 0.11791 | 0.00774705 |  |
| 63 | 3780 | 0.12428 | 0.00816558 |  |
| 68 | 4080 | 0.13463 | 0.00884561 |  |
| 73 | 4380 | 0.14841 | 0.00975100 |  |


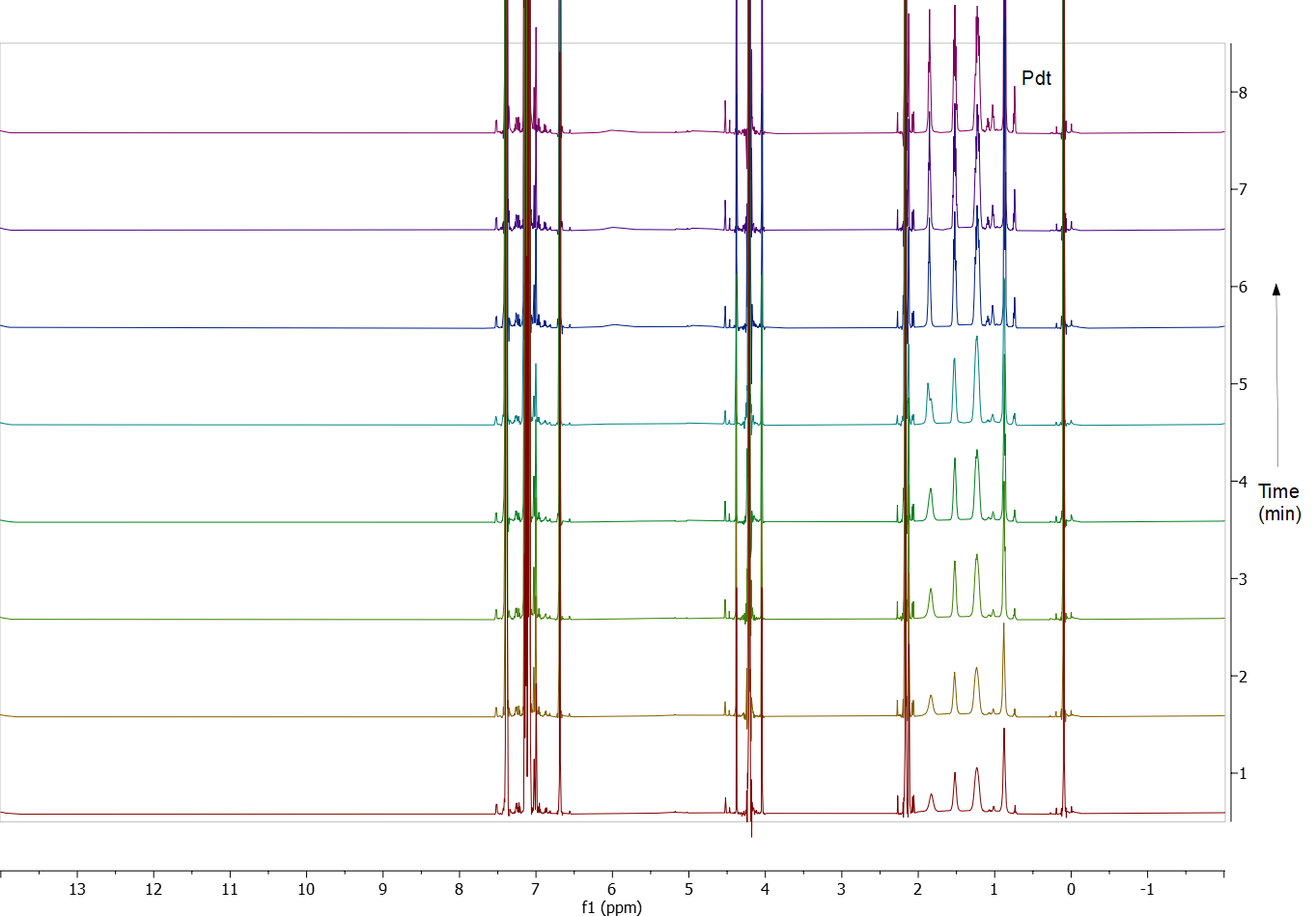


Figure S78. Stacked ^1^H NMR spectra for reaction with 7.5 eq PhSiH_3_.

| Table S19. Raw data of ^1^H NMR spectra for 7.5 eq PhSiH_3_ | | | | |
| --- | --- | --- | --- | --- |
| 7.5 eq PhSiH3 | 1.574 M |  |  | k_obs_ (M/s) |
| time (min) | time (sec) | integral | [pdt] (M) | 3.29E-06 |
| 15 | 900 | 0.04129 | 0.00271288 |  |
| 20 | 1200 | 0.04541 | 0.00298358 |  |
| 30 | 1800 | 0.07132 | 0.00468595 |  |
| 35 | 2100 | 0.08861 | 0.00582195 |  |
| 40 | 2400 | 0.11008 | 0.00723260 |  |
| 64 | 3840 | 0.18813 | 0.01236073 |  |
| 70 | 4200 | 0.20313 | 0.01334627 |  |
| 80 | 4800 | 0.22159 | 0.01455915 |  |


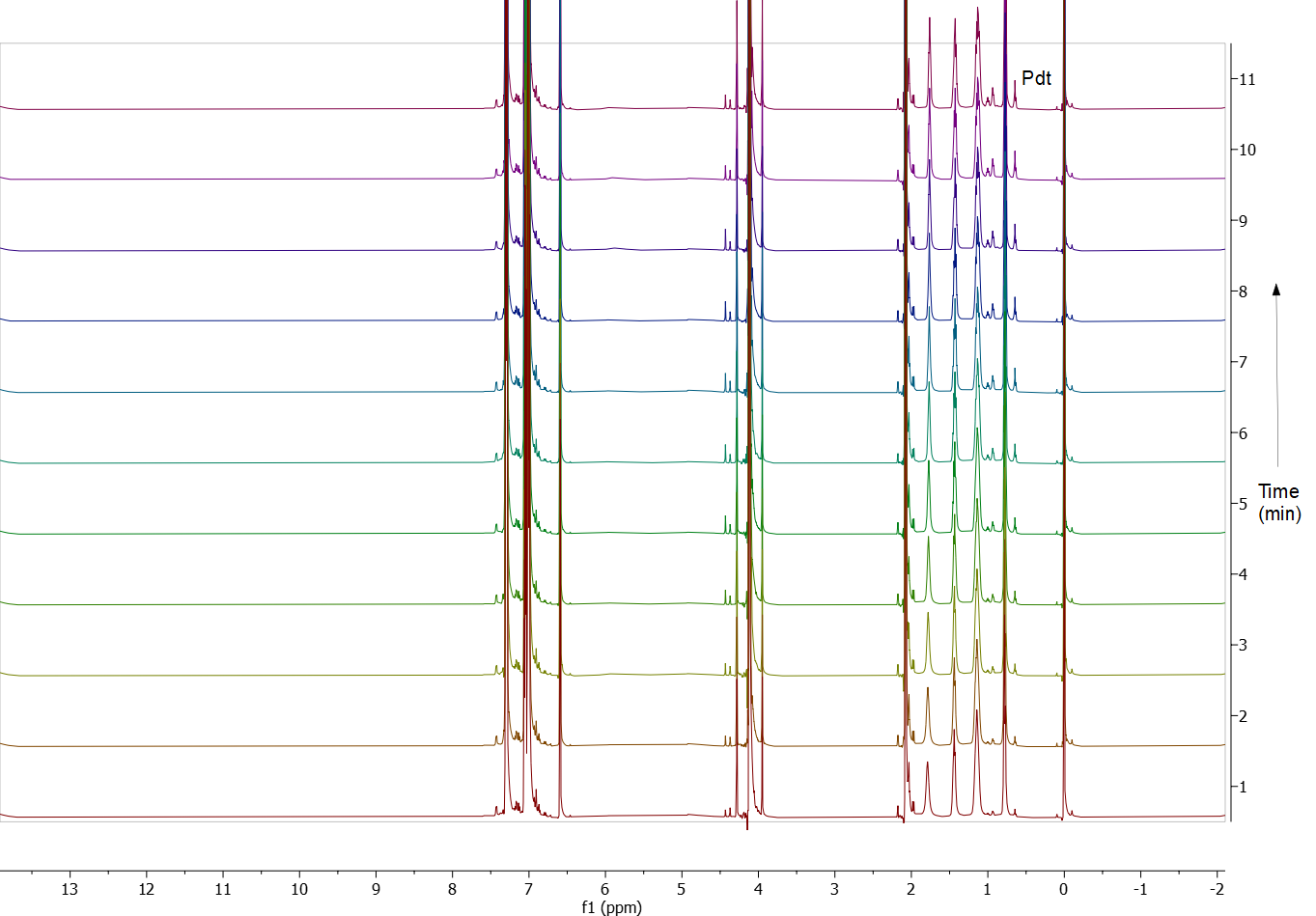


Figure S79. Stacked ^1^H NMR spectra for reaction with 10 eq PhSiH_3_.

| Table S20. Raw data of ^1^H NMR spectra for 10 eq PhSiH_3_ | | | | |
| --- | --- | --- | --- | --- |
| 10 eq PhSiH3 | 2.1 M |  |  | k_obs_ (M/s) |
| time (min) | time (sec) | integral | [pdt] (M) | 3.72E-06 |
| 15 | 900 | 0.04054 | 0.00266360 |  |
| 20 | 1200 | 0.05697 | 0.00374311 |  |
| 25 | 1500 | 0.07615 | 0.00500329 |  |
| 30 | 1800 | 0.09580 | 0.00629436 |  |
| 35 | 2100 | 0.12082 | 0.00793825 |  |
| 45 | 2700 | 0.14545 | 0.00955652 |  |
| 50 | 3000 | 0.17038 | 0.01119450 |  |
| 55 | 3300 | 0.18492 | 0.01214982 |  |
| 65 | 3900 | 0.21477 | 0.01411106 |  |
| 70 | 4200 | 0.22860 | 0.01501973 |  |
| 75 | 4500 | 0.24089 | 0.01582722 |  |

| Table S21. Data of ln(k_obs_) vs. ln[PhSiH_3_] | |
| --- | --- |
| ln[Ph-SiH_3_] | ln(k_obs_) |
| 0.11511281 | -12.764689 |
| 0.45362015 | -12.624623 |
| -0.64511920 | -13.036186 |
| 0.74193734 | -12.501787 |

## 3.2 Investigation of the Siloxane Byproducts

Identification of the siloxane byproducts was classified into two categories, for which the silane with single Si-H bonds yields R_3_Si-O-SiR_3_ product, while the silane with multiple Si-H bonds yields a mixture of polymeric siloxane products.

Analysis of the R_3_Si-O-SiR_3_ product was conducted by the standard catalytic conditions detailed in **1.3** where dimethoxymethylsilane was used as shown in Figure **S80**. ^29^Si NMR (Figure **S81**) identified the corresponding DMM-siloxane byproduct at -48.52 ppm.^[21]^ The peak at -40.14 ppm is attributed to the impurity already present in the starting DMMS material, most likely a hydrolyzed DMMS impurity. Moreover, the reaction mixture was quenched with water and then extracted with DCM to perform Gas Chromatography Mass Spectroscopy (GCMS), where GCMS confirmed the corresponding siloxane species as well (Figure **S82**).

Figure S80. The scheme of silylative dehydration with dimethoxymethylsilane (DMMS).


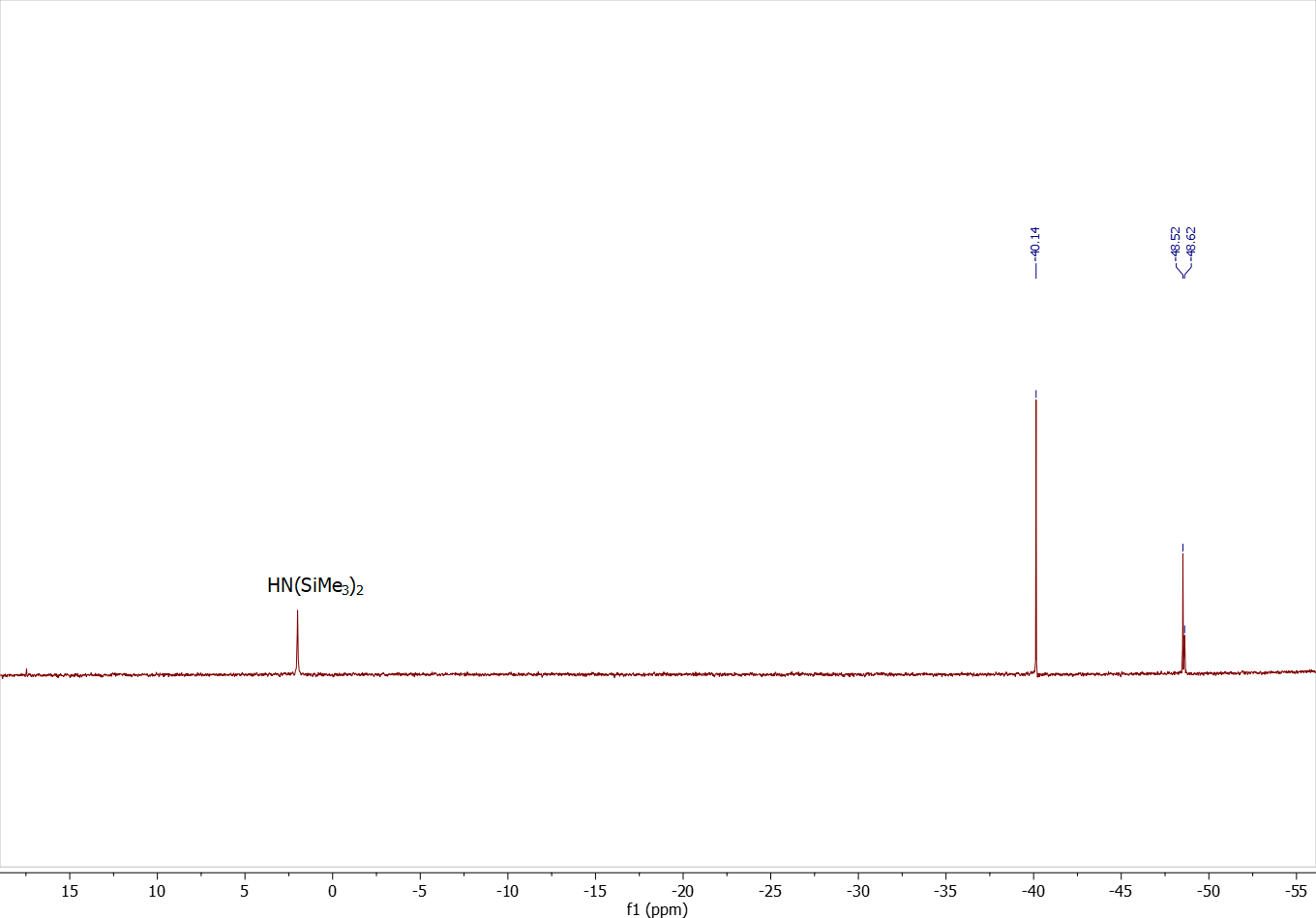


Figure S81. The ^29^Si NMR (99 MHz, Toluene-*d*_8_) of reaction in Figure S80 using DMMS.


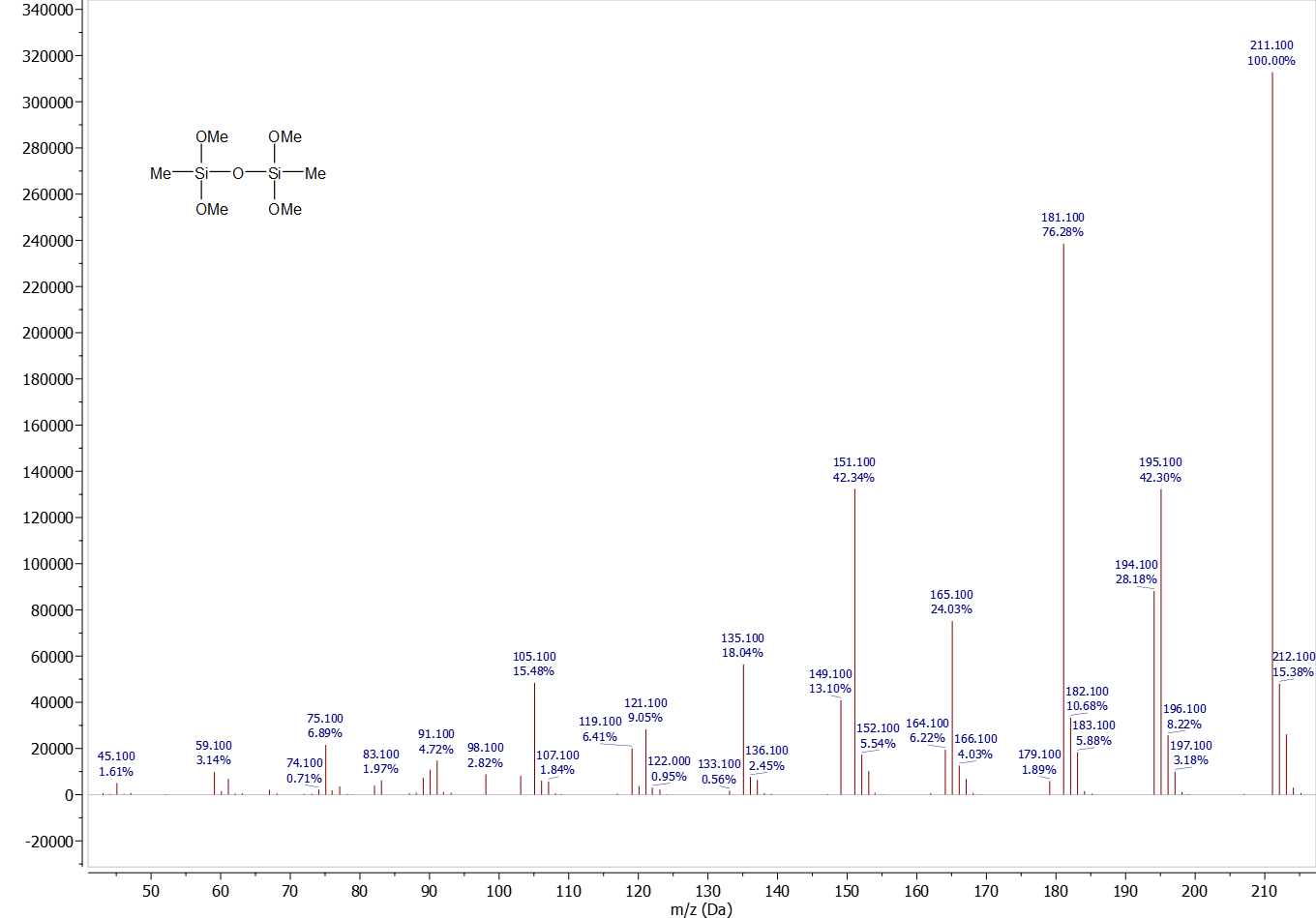


Figure S82. The HRMS spectrum of dimethyltetramethoxydisiloxane identified.

In contrast, the regular reaction conditions using PhSiH_3_ yields a mixture of polymeric siloxane products, since multiple Si-H bonds are available to react. Spectroscopic details of the sub-stoichiometric reaction are presented below.


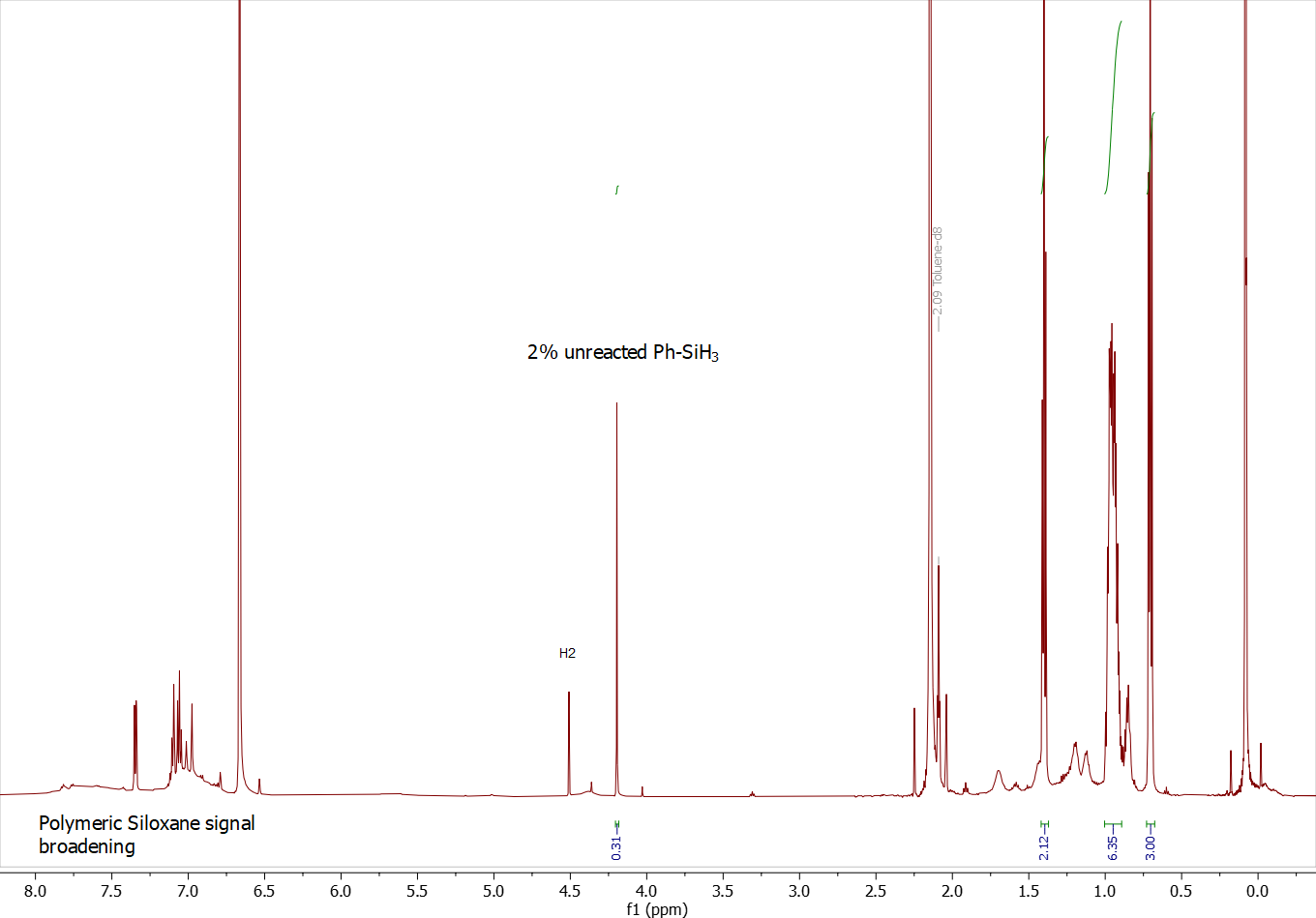


Figure S83. The ^1^H NMR of sub-stoichiometric reaction in Figure S80 but using 0.6 equiv PhSiH_3_.


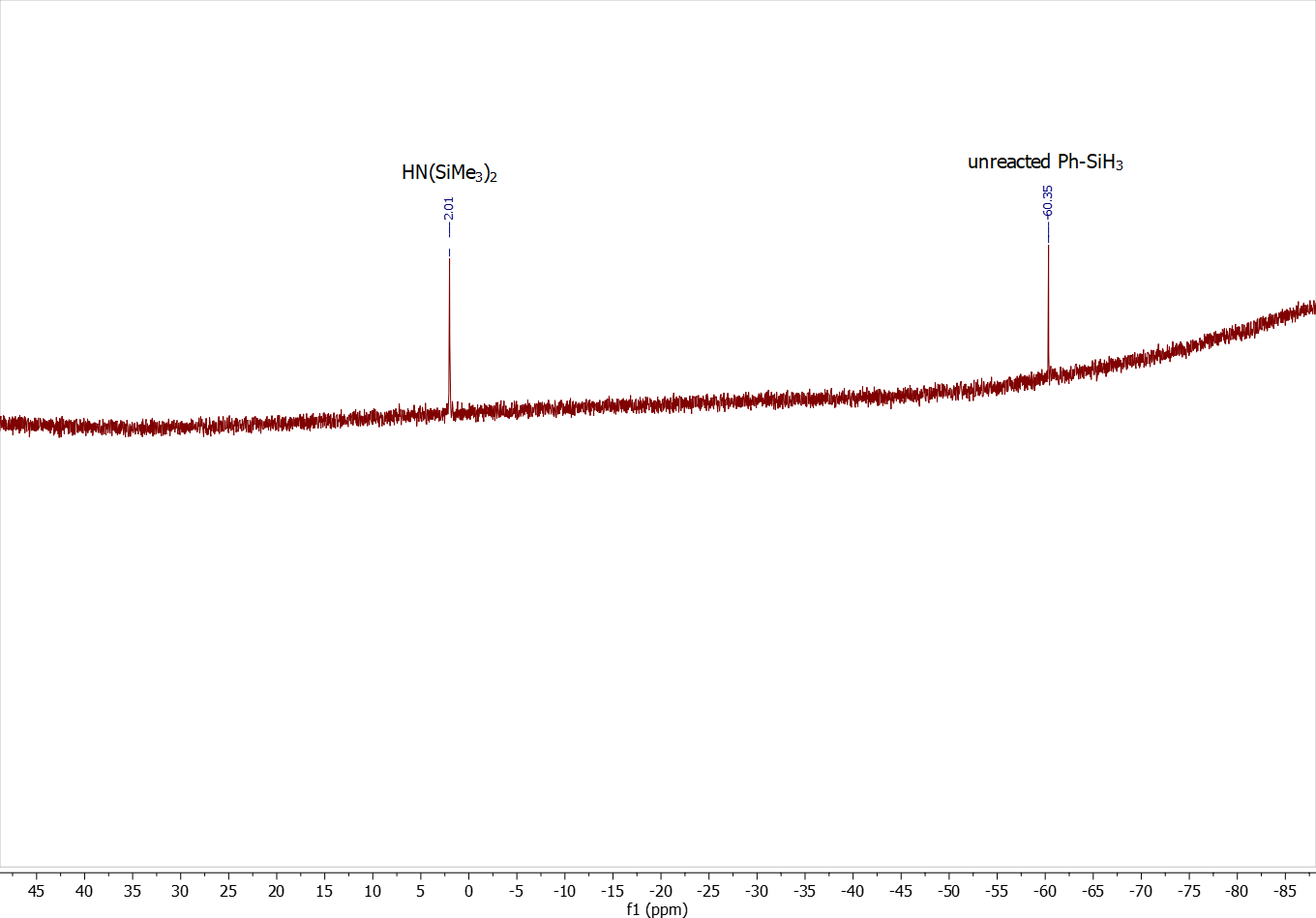


Figure S84. The ^29^Si NMR of sub-stoichiometric reaction in Figure S80 but using 0.6 equiv PhSiH_3_.

## 3.3 Determination of Activation Parameters

Plotting the data points of product concentrations at different temperatures from 40 to 80 ºC while keeping other parameters consistent will yield specific k_obs_ values, which were converted to coefficient $k$ using equation **S3**. The activation parameters enthalpy of activation $\Delta H^{\ddagger} (kcal{\cdot mol}^{-1})$, entropy of activation $\Delta S^{\ddagger} (kcal{\cdot K}^{-1}{\cdot mol}^{-1})$ and activation energy $E_{a} (kcal{\cdot mol}^{-1})$ were determined then by analyzing the correlation between coefficient $k$ ($M^{-1}s^{-1})$ and temperature $T$ ($K)$ as suggested by equations Eyring equation **S4** and Arrhenius equation **S5**, where $k_{B}$($J{\cdot K}^{-1})$ is Boltzmann constant, $h$ ($J{\cdot s}^{-1})$ is Planck’s constant, $R (kcal{\cdot K}^{-1}{\cdot mol}^{-1})$ is Ideal Gas constant, A is pre-exponential factor ($M^{-1}s^{-1})$, $M$ is molarity and $m (m=2)$ is the molecularity of the reaction for unit balancing.

$$k=\frac{k_{obs}}{\left[ hexanamide \right]^{0}\cdot\left[ {La}^{NTMS} \right]^{1}\cdot\left[ PhSiH_{3} \right]^{1}} S3$$

$ln\frac{k}{T(M^{1-m})}=-\frac{\Delta H^{\ddagger}}{R}\frac{1}{T}+ln\frac{k_{B}}{h}+\frac{\Delta S^{\ddagger}}{R} S4$

$$lnk=-\frac{E_{a}}{RT}+lnA S5$$

| Table S22. Reaction rate and raw data at different temperatures | | | | | |
| --- | --- | --- | --- | --- | --- |
| T ($K$) | k_obs_ ($Ms^{-1}$) | k ($M^{-1}s^{-1})$ | ln (k/T) | 1/T ($K^{-1})$ | ln (k) |
| 313.15 | 2.43e-7 | 7.19e-7 | -19.89 | 3.19e-3 | -14.15 |
| 323.15 | 1.28e-6 | 3.79e-6 | -18.26 | 3.09e-3 | -12.48 |
| 328.15 | 2.22e-6 | 6.57e-6 | -17.73 | 3.05e-3 | -11.93 |
| 333.15 | 3.27e-6 | 9.67e-6 | -17.35 | 3.00e-3 | -11.55 |
| 343.15 | 5.41e-6 | 1.60e-5 | -16.88 | 2.91e-3 | -11.04 |
| 353.15 | 1.40e-5 | 4.14e-5 | -15.96 | 2.83e-3 | -10.09 |

Referring to Figure S85 and S86, calculations of the activation parameters were made available using equations S4 and S5 that yield $\Delta H^{\ddagger}=20.1 (1.9) \mathrm{kcal}\mathrm{mol}^{-1}$, $\Delta S^{\ddagger}=-21.7 (5.7) cal\mathrm{mol}^{-1}K^{-1}$ and $E_{a}=20.8 (1.9) kcal\mathrm{mol}^{-1}$.


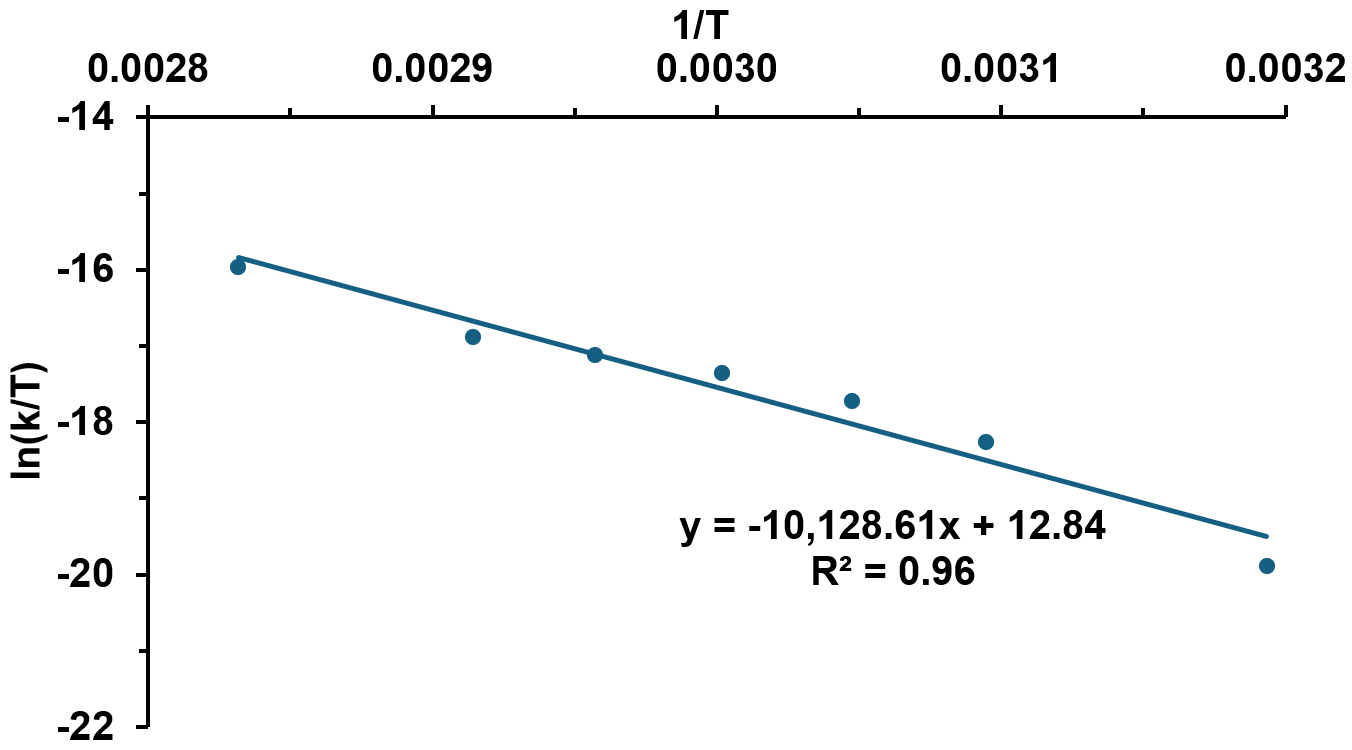


Figure S85. Eyring plot of ln(k/T) vs. 1/T.


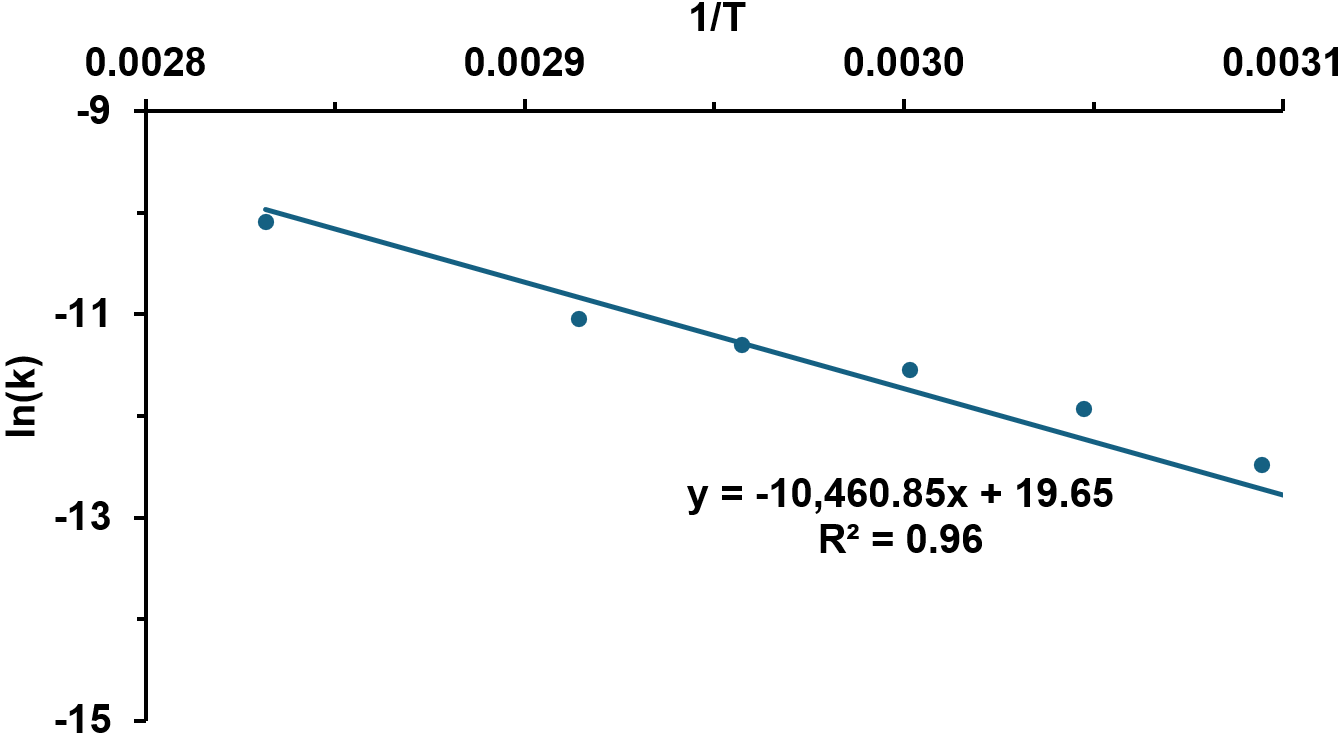


Figure S86. Arrhenius plot of ln(k) vs. 1/T.

## 3.4 Kinetic Isotope Effect Experiment for Phenylsilane

The general procedure of the KIE experiment follows NMR scale kinetic analysis in **3.1** for the catalytic conditions as shown in Figure **S87**, where the PhSiD_3_ was synthesized using previously reported procedures.^[22]^ The reaction rate k_Si-H_ for PhSiH_3_ is $1.59\times{10}^{-6}M\cdot s^{-1}$, and the reaction rate k_Si-D_ for PhSiD_3_ is $1.13\times{10}^{-6}M\cdot s^{-1}$. The experimental KIE value is determined as k_Si-H_/ k_Si-D_ = 1.4$\pm$0.1.


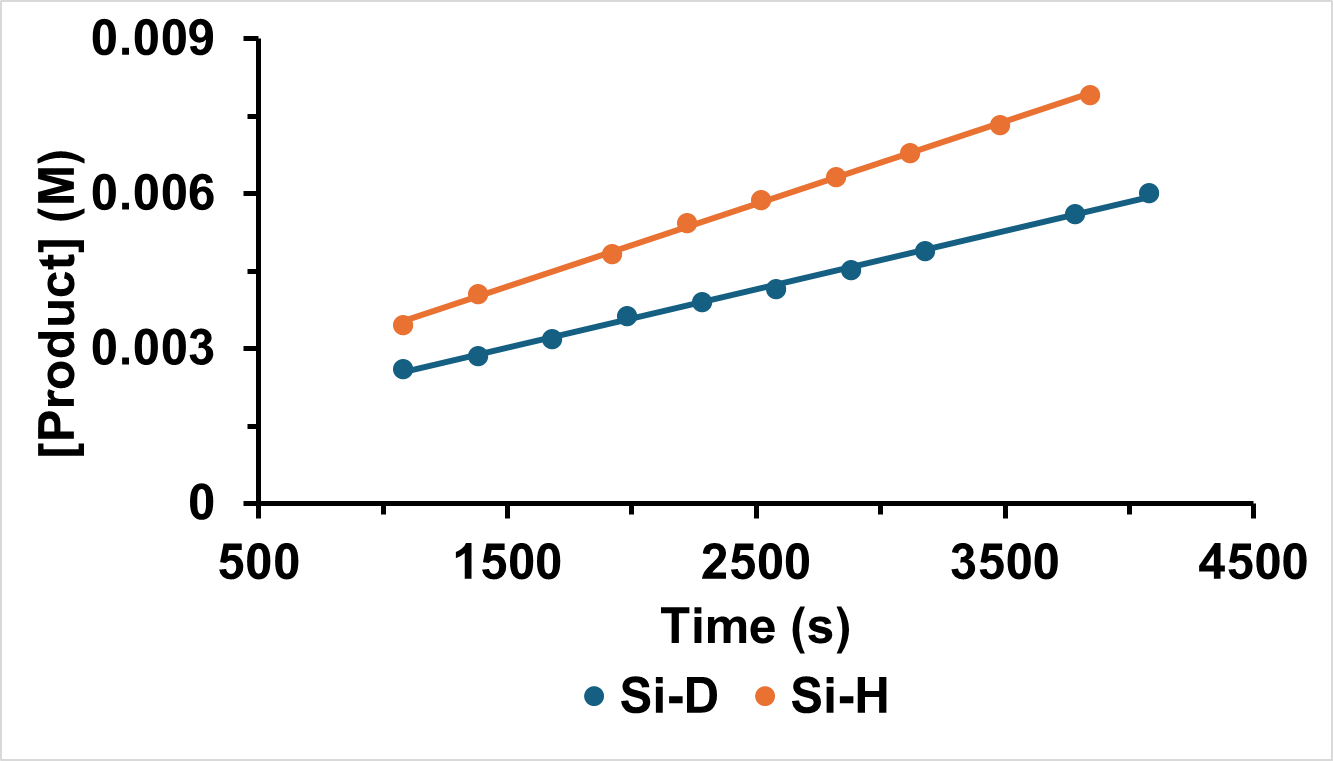


Figure S87. Plot of initial rates for PhSiD_3_ and PhSiH_3_.

**PhSiD_3_**

^1^H NMR (400 MHz, Toluene-*d*_8_) δ 7.39 – 7.30 (m, 2H), 7.12 – 7.01 (m, 3H).

^13^C NMR (101 MHz, Toluene-*d*_8_) δ 137.43, 136.06, 129.96, 128.29.

^2^H NMR (92 MHz, Toluene-*d*_8_) δ 4.24.

^29^Si NMR (99 MHz, Toluene-*d*_8_) δ -60.92 (hept, *J* = 30.6 Hz).

Figure S88. ^1^H NMR (400 MHz, Toluene-*d*_8_) of PhSiD_3_.

Figure S89. ^13^C NMR (101 MHz, Toluene-*d*_8_) of PhSiD_3_.

Figure S90. ^2^H NMR (92 MHz, Toluene-*d*_8_) of PhSiD_3_.

Figure S91. ^29^Si NMR (99 MHz, Toluene-*d*_8_) of PhSiD_3_.

## 3.5 Protonolysis Experiments

A set of experiments was performed to investigate structural and compositional aspects of the possible catalyst resting state. A protonolysis experiment was conducted because the strongly basic N(SiMe_3_)_2_ ligands are likely to deprotonate the primary amide under stoichiometric conditions, leading to amidate coordination to the La center. Therefore, various equivs of hexanamide and 1 equiv of La[N(SiMe_3_)_2_]_3_ were mixed in toluene-d_8_ and allowed to react at room temperature for 24 h. As indicated in Figure **S92**, ^1^H NMR reveals that the corresponding nitrile is readily produced under such stochiometric conditions, following a general trend that increased amounts of the starting hexanamide suppress the amounts of nitrile produced, reflecting increased amounts of amidate coordination (Figure **S93**). Note that 3 equivalents of HN(SiMe_3_)_2_ free ligand are always produced, regardless of the stoichiometric variation, indicating full ligand displacement from La[N(SiMe_3_)_2_]_3_. When reaching the catalytic stoichiometry of 20 equiv of amide relative to La[N(SiMe_3_)_2_]_3_, the amount of nitrile product is negligible so that majority of the hexanamide is converted to the amidate ligands coordinating to the La metal center. Note that nitrile is further generated upon heating -- up to 1 equiv of nitrile. Based on a previously reported crystal structure which sheds light on Y-trisamidate interactions,^[23]^ we propose a full ligand exchange during the reaction that yields La-trisamidate structure as the catalyst resting state as in Figure **S94**.

|  |
| --- |
|  |
| Figure S92. Equivalents of nitrile produced relative to the initial equivalents of added amides.  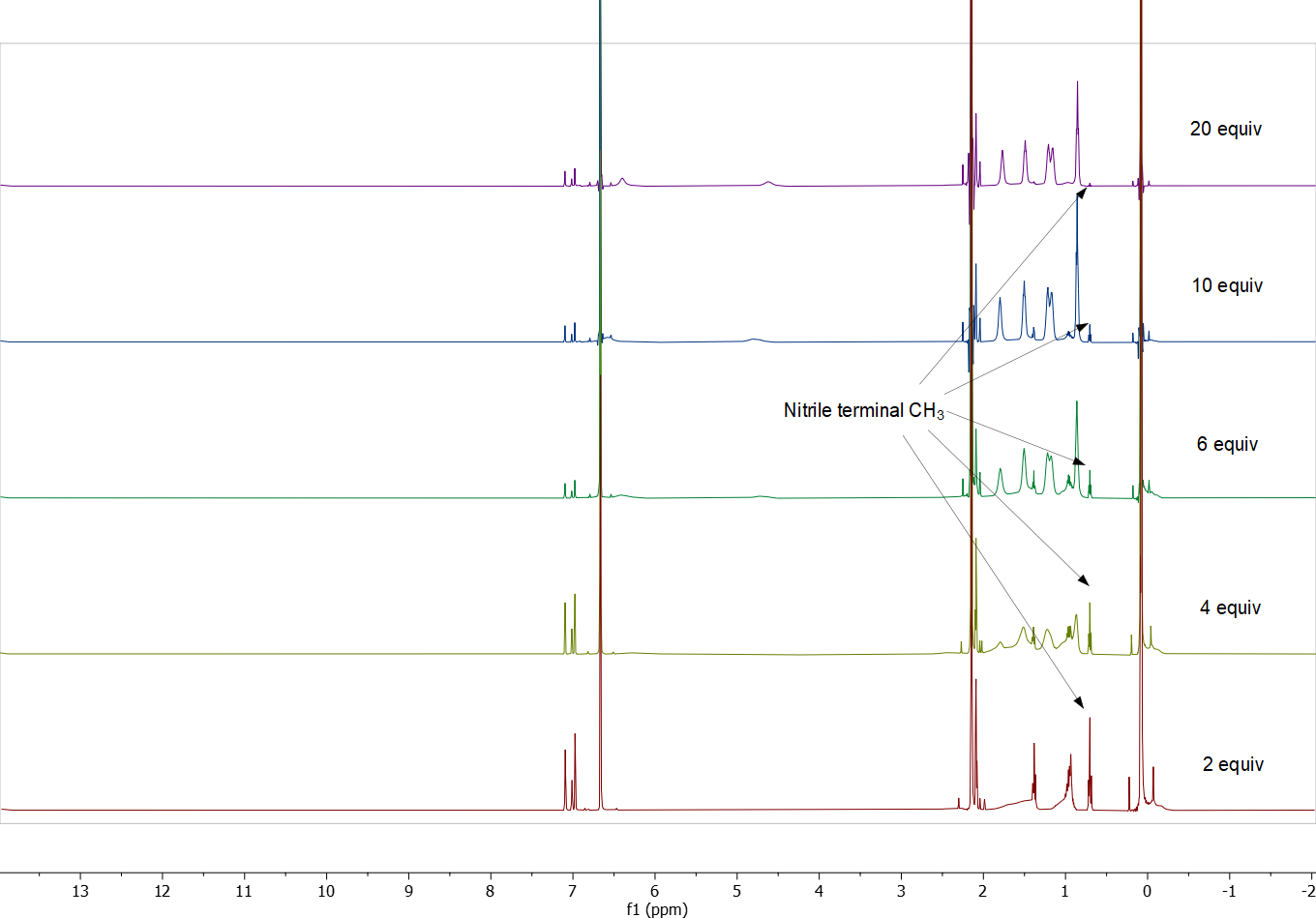  Figure S93. Comparison of ^1^H NMR spectra in toluene-d_8_ with increasing equivs of hexanamide added. |
|  |
| Figure S94. Proposed simplest structure of the La-amidate resting state under catalytic conditions (up to 20 equiv hexanamide to 1 equiv La^NTMS^). R = CH_3_(CH2)_4_- |

## 3.6 Isolation and Characterization of the La-acetamidate active species

A scaled up synthesis of the La-acetamidate species is outlined in Figure **S95**, for which 200 mg acetamide and 105 mg La^NTMS^ were added to an oven-dried 50 mL Schlenk tube in the glovebox. Then dry THF was vacuum transferred from the storage container to the Schlenk tube using a high-vac line, and the solution was allowed to stir at room temperature for 24 h. The acetamide substrate was dried previously by dissolving in THF solvent, adding 3Å molecular sieves, allowing to stand for 5 days, then removing the THF solvent under high-vac to obtain dry acetamide.

Workup procedures to isolate the La-acetamidate solid had two steps. The first involved removal of THF and other volatiles, which was done by connecting the Schlenk tube to the high-vac line and then opening to the vacuum and transferring the volatiles to a liquid nitrogen trap. This yielded a white solid mixture of La-acetamidate, unreacted acetamide, and HNTMS_2_ free ligand. The second step involved removal of unreacted acetamide and HNTMS_2_ ligand, for which the Schlenk tube with white powder mixture at the bottom was submerged in an oil bath and heated to 80ºC while still connected to the active vacuum at 10^-5^ Torr. This leads to vacuum transfer of the HNTMS_2_ and slow sublimation of unreacted acetamide to condense as crystals in the upper part of the Schlenk flask, leaving the La-acetamidate species at the bottom of the flask. (Figure **S96**) The flask was left under vacuum overnight for full separation of species, and then transferred to the glovebox so that La-acetamidate white powder was scratched off the bottom of the flask and collected with a spatula. Further characterization of the La-trisacetamidate white solid was conducted by a hydrolysis experiment, elemental anlaysis, and testing catalytic performance. (Figure **S97-S102**)

**Figure S95.** Scheme of the synthesis of La-acetamidate species.


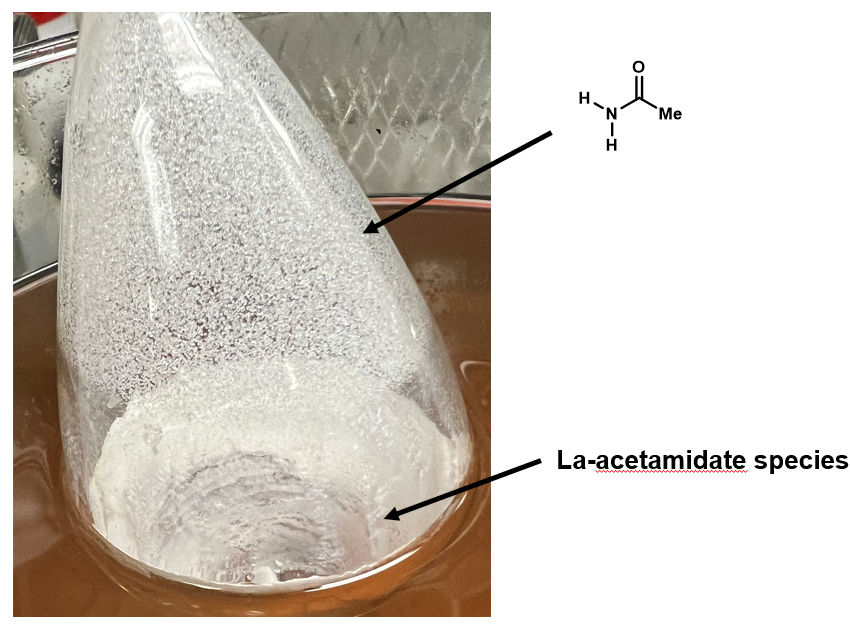


**Figure S96.** The La-acetamidate powder at bottom, the crystals of unreacted acetamide at the top.

Based on the protonolysis experiment that nitrile formation is negligible at 20 eq. amide vs. 1 eq. La^NTMS^ conditions, we propose a full ligand exchange into three acetamidate ligands coordinating the La center, yielding La-trisacetamidate. However, the hydrolysis experiment revealed 2.5 eq. acetamides that are displaced from the La center (Figure **S97**), indicating that additional nitrile formation during the synthesis and isolation offers lower than expected stoichiometry for the amidate ligands on La. The observation is further validated by the results obtained from ICP-OES and combustion elemental analysis as shown in **Table S23** that C,H,N values are consistently lower thane proposed La-trisamidate, while consistent with the fitting of 2.5 equivalents of acetamidate and an additional 0.5 equivalent of oxygen due to the nitrile formation, yielding LaC_5_H_10.5_O_3_N_2.5_ for the isolated species. More importantly, the isolated species is catalytically active as shown in Figure **S100**, and full ligand exchange with the hexanamide occurs in the presence of silane because the trace amount of MeCN that comes from the initial acetamidate ligands are observed in the NMR of final products (Figure **S101**, **S102**).

**Figure S97.** Hydrolysis experiment of the La-acetamidate analysis.


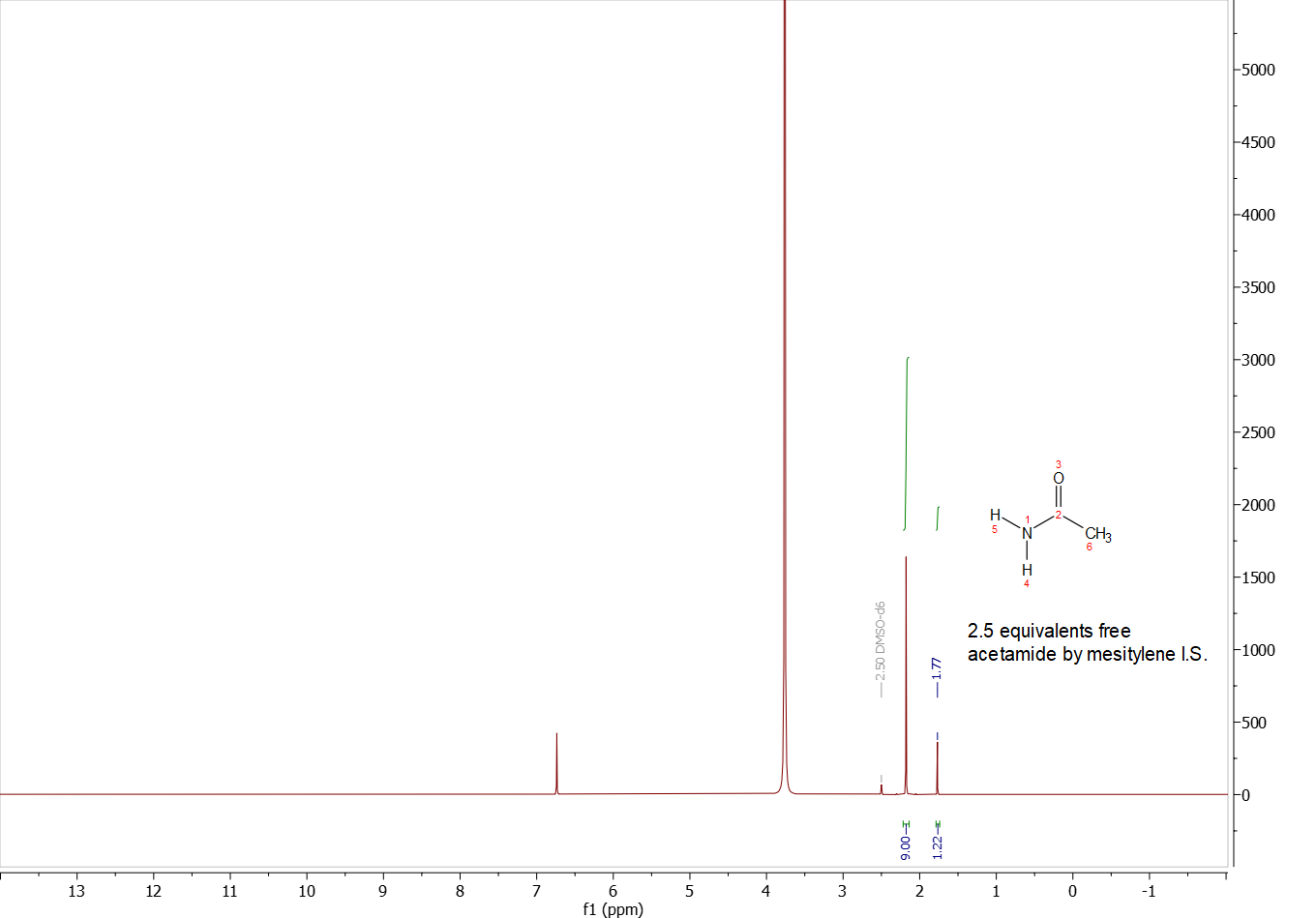


**Figure S98.** ^1^H NMR (600 MHz, Toluene-*d*_8_) spectrum of the hydrolysis experiment.


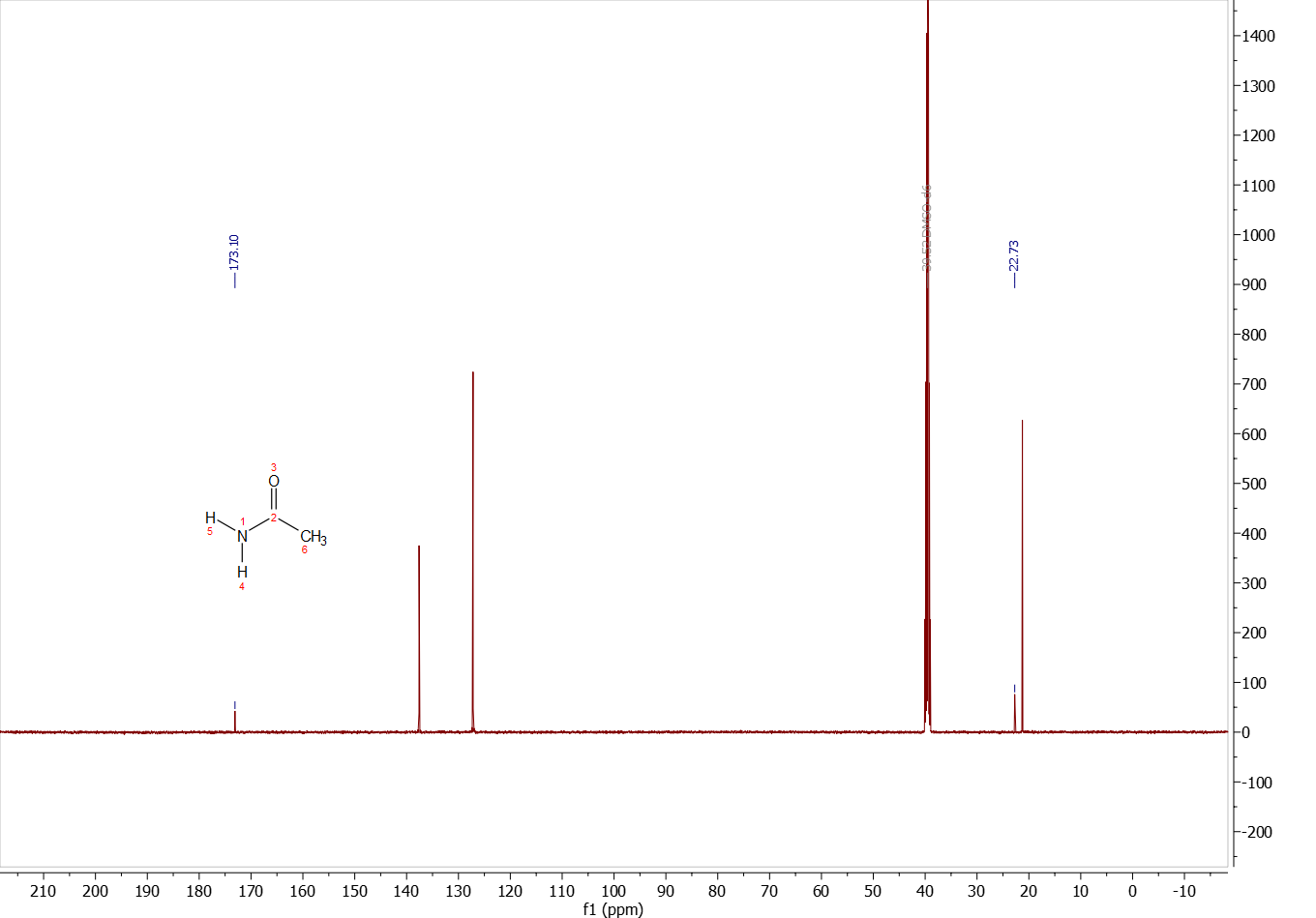


**Figure S99**. ^13^C (151 MHz, DMSO-*d*_6_) NMR spectrum of the hydrolysis experiment.

| Table S23. Comparison of the weight percentage for different elements | | | | |
| --- | --- | --- | --- | --- |
| Elements | La-trisamiadate (wt%) | ICP-OES (wt%) | Elemental Analysis (wt%)  (4 trials) | LaC_5_H_10.5_O_3_N_2.5_ |
| La | 44.37 | 46.11% ($\pm$5.3%) | / | 47.48 |
| C | 23.02 | / | 19.29, 19.31, 19.56, 20.92 | 20.53 |
| H | 3.86 | / | 3.67, 3.73, 3.73, 5.06 | 3.62 |
| O | 13.42 | / | / | 16.41 |
| N | 15.33 | / | 10.24, 10.56, 11.01, 14.25 | 11.97 |

**Figure S100.** Amide to nitrile conversion using the isolated La-acetamidate as the catalyst.


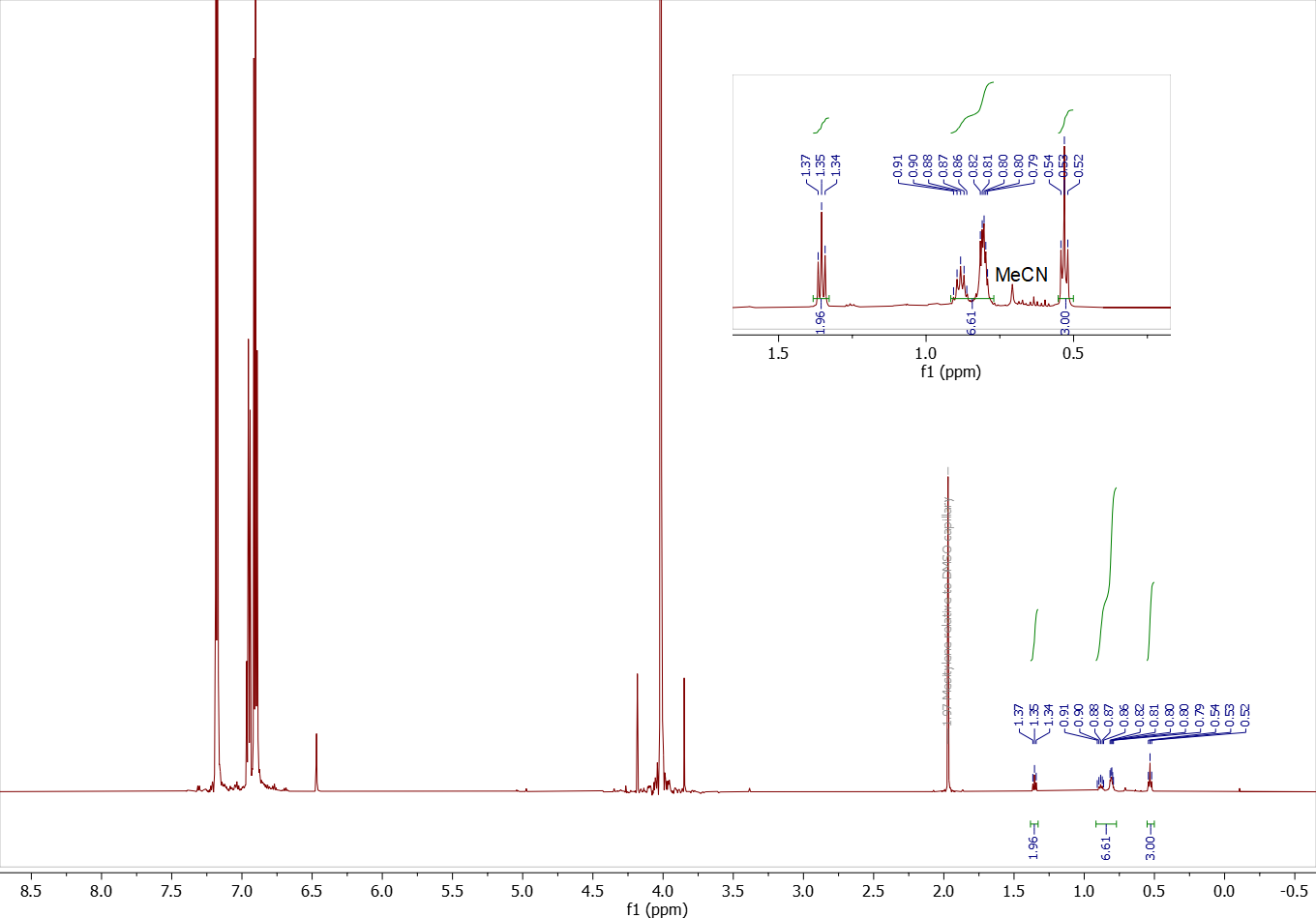


**Figure S101.** ^1^H NMR (600 MHz, Toluene-*d*_8_) spectrum of the amide to nitrile conversion catalytic reaction using LaC_5_H_10.5_O_3_N_2.5_ as the catalyst.


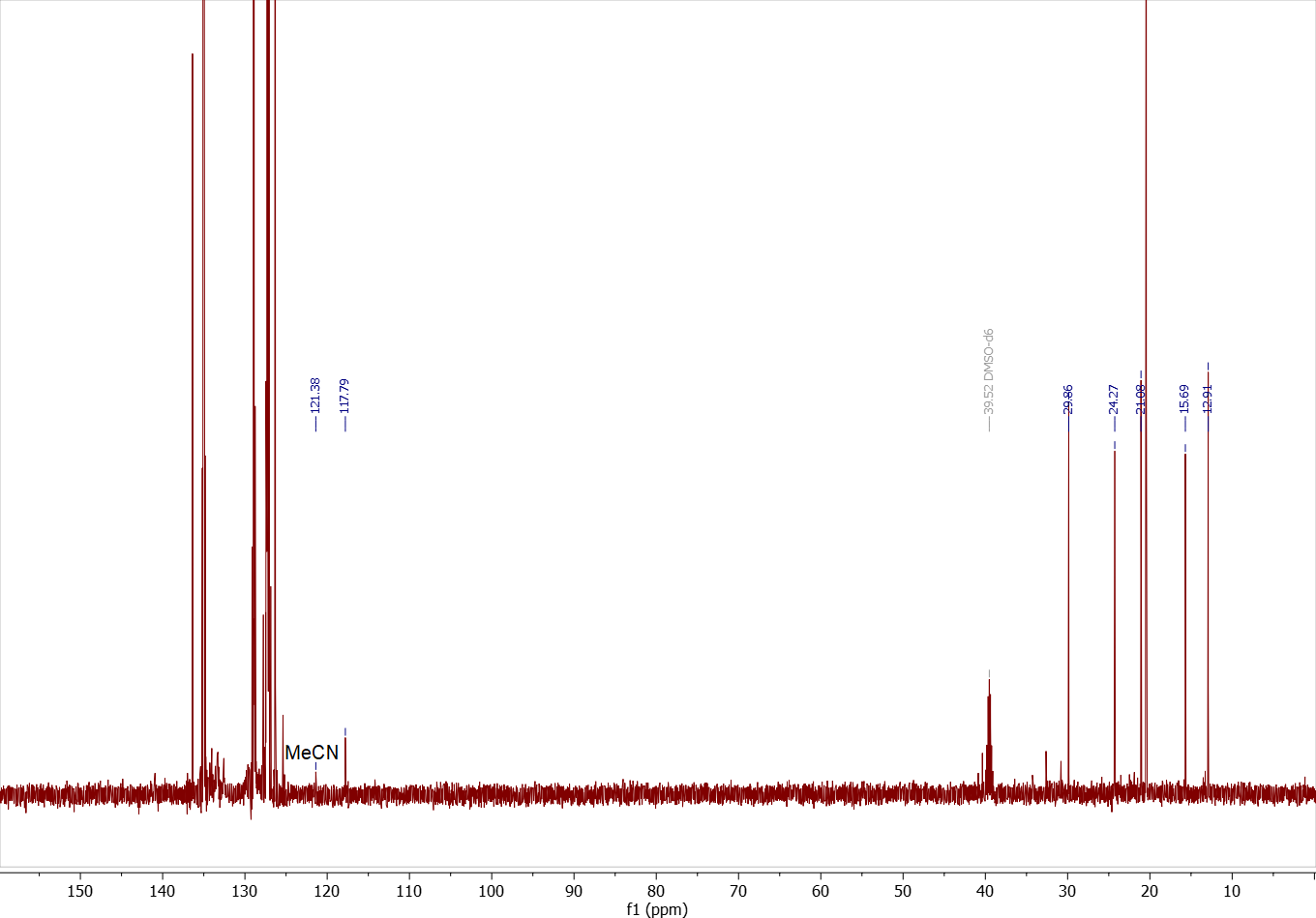


**Figure S102.** ^13^C NMR (151 MHz, DMSO-*d*_6_) spectrum of the amide to nitrile conversion catalytic reaction using LaC_5_H_10.5_O_3_N_2.5_ as the catalyst.

## 3.7 Computational Details

Geometry optimizations of all reactants, products, intermediates and transition states were carried out along the entire catalytic cycle. Calculations were performed adopting the M06 hybrid meta-GGA functional.^[24]^ The effective core potential of Hay and Wadt (LANL2DZ) and the relative basis set were used for the lanthanum and silicon atoms.^[25]^ The standard all-electron 6-31G** basis was used for all remaining atoms.^[26]^ Molecular geometry optimization of stationary points was carried out without symmetry constrains and used analytical gradient techniques. The transition states were searched with the “distinguished reaction coordinate procedure” along the emerging bonds. Frequency analysis was performed to obtain thermochemical information about the reaction pathways at 298 K upon the harmonic approximation. All calculations were performed using G16 code on Linux cluster systems.^[27]^ Predicted KIE values are evaluated at 60°C starting from the frequency analysis (as computed by G16 package) of the reactants and transition states involved in all the process steps in order to obtain the Bigeleisen-Mayer Reduced Isotopic Partition Function Ratios by means of the kinisot code.^[28]^

**Figure S103**. Calculated energy barriers of the turn-over determining step for La-trisamidate complexes with different coordination numbers of neutral amides.

## 3.8 Cartesian Coordinates (Å) of all Presented Species

Species 1

LaC10N5H22O5

La 0.073921000 0.160745000 -0.210657000

O -2.024106000 1.127708000 -1.376044000

C -1.556891000 1.008132000 -2.563152000

N -0.325846000 0.584529000 -2.720405000

H -0.021800000 0.523618000 -3.684023000

O -1.368145000 -1.955508000 -0.286765000

C -0.532728000 -2.706147000 0.317849000

N 0.639305000 -2.224766000 0.679811000

H 1.213924000 -2.888969000 1.184970000

C -2.471020000 1.367846000 -3.704152000

H -1.989723000 1.271595000 -4.681813000

H -3.353422000 0.719796000 -3.674030000

H -2.822336000 2.396462000 -3.572817000

C -0.952936000 -4.132508000 0.577242000

H -1.898506000 -4.136612000 1.129423000

H -1.145186000 -4.626368000 -0.381103000

H -0.206395000 -4.707040000 1.134648000

O -1.644462000 0.374404000 1.661134000

C -2.850640000 0.081696000 1.690612000

N -3.694141000 0.393703000 0.702456000

H -4.638956000 0.046149000 0.720686000

C -3.403918000 -0.657138000 2.874431000

H -3.038922000 -1.689185000 2.824792000

H -3.004298000 -0.213565000 3.789503000

H -4.496938000 -0.668236000 2.914135000

H -3.301740000 0.751766000 -0.178487000

O 1.667638000 1.052644000 1.630442000

C 1.438826000 2.298376000 1.402205000

N 0.648341000 2.631756000 0.414609000

H 0.492416000 3.628719000 0.327399000

C 2.107160000 3.291865000 2.315613000

H 1.867668000 4.329423000 2.064606000

H 1.803442000 3.089614000 3.347910000

H 3.192917000 3.153941000 2.268416000

O 2.435187000 -0.261463000 -1.133134000

C 3.411806000 -0.779138000 -0.568182000

N 3.777514000 -0.453276000 0.675601000

H 4.539309000 -0.932459000 1.126842000

C 4.214470000 -1.826283000 -1.284109000

H 3.596600000 -2.728392000 -1.355313000

H 4.413055000 -1.487264000 -2.303736000

H 5.156649000 -2.075601000 -0.787539000

H 3.158895000 0.174024000 1.213283000

Transition State 1-2

LaSiC16N5H30O5

La -1.072045000 -0.158144000 -0.111253000

O -2.321134000 1.352619000 1.552966000

C -2.393368000 0.479283000 2.493565000

N -2.185092000 -0.781966000 2.210584000

H -2.153357000 -1.373888000 3.033210000

O -0.498842000 -2.638658000 -0.475134000

C 0.030931000 -2.473138000 -1.637060000

N -0.074107000 -1.309139000 -2.229180000

H 0.406215000 -1.260145000 -3.119886000

C -2.676841000 0.993424000 3.880374000

H -1.840419000 1.621610000 4.205011000

H -3.567784000 1.629491000 3.857805000

H -2.823030000 0.191294000 4.609887000

C 0.797178000 -3.634537000 -2.208312000

H 1.105216000 -3.471413000 -3.245296000

H 1.689493000 -3.797132000 -1.592609000

H 0.191881000 -4.544328000 -2.145529000

H 0.751820000 -0.186948000 1.302514000

O 0.503678000 1.800770000 -0.415862000

C 1.566277000 2.245036000 0.060183000

N 2.540454000 1.492879000 0.577518000

H 3.383259000 2.001226000 0.819896000

C 1.775664000 3.737415000 0.023088000

H 2.710525000 4.067694000 0.484055000

H 1.749706000 4.071376000 -1.018849000

H 0.933017000 4.213097000 0.534679000

Si 2.679329000 -0.349019000 0.794147000

H 2.727666000 -0.979100000 2.132312000

H 2.187030000 -1.108488000 -0.379148000

C 4.599155000 -0.311403000 0.454539000

C 5.498291000 -0.800726000 1.405570000

C 5.134444000 0.166203000 -0.749018000

C 6.873116000 -0.810415000 1.177978000

C 6.504093000 0.162134000 -0.992502000

C 7.378244000 -0.326924000 -0.024046000

H 5.111684000 -1.185813000 2.352084000

H 4.459812000 0.550435000 -1.518441000

H 7.551684000 -1.194817000 1.937824000

H 6.894783000 0.537047000 -1.937338000

H 8.450817000 -0.332360000 -0.208768000

O -3.346281000 -1.264211000 -0.639384000

C -3.807882000 -2.240548000 -0.028136000

N -3.126342000 -3.385010000 0.109024000

H -3.484726000 -4.120967000 0.695638000

C -5.165110000 -2.156119000 0.604278000

H -5.056969000 -1.539628000 1.504323000

H -5.847210000 -1.638366000 -0.073847000

H -5.582550000 -3.128343000 0.882409000

H -2.131377000 -3.379364000 -0.153558000

O -2.239016000 1.574124000 -1.607207000

C -2.635951000 2.741940000 -1.497907000

N -2.899330000 3.310589000 -0.315072000

H -3.227746000 4.260491000 -0.265399000

C -2.837722000 3.574270000 -2.732337000

H -1.877463000 3.665420000 -3.248656000

H -3.514354000 3.040205000 -3.405250000

H -3.237011000 4.572682000 -2.532295000

H -2.784094000 2.755537000 0.541868000

Species 2

LaSiC16N5H30O5

La 1.072483000 -0.135822000 -0.221468000

O 2.066715000 2.229867000 0.070700000

C 3.033561000 2.021044000 -0.750940000

N 3.462575000 0.798303000 -0.942202000

H 4.128437000 0.742902000 -1.706503000

O 1.671591000 -2.508106000 -1.044976000

C 0.601936000 -3.071262000 -0.611388000

N -0.323426000 -2.345393000 -0.029456000

H -1.127655000 -2.883711000 0.272828000

C 3.541279000 3.212404000 -1.519371000

H 2.803122000 3.460200000 -2.292720000

H 3.628062000 4.077450000 -0.855021000

H 4.503114000 3.024933000 -2.006228000

C 0.491186000 -4.560528000 -0.807235000

H -0.453930000 -4.970847000 -0.439301000

H 0.596842000 -4.790761000 -1.872356000

H 1.320549000 -5.053718000 -0.288314000

H 0.649099000 0.501790000 -2.380140000

O -1.165076000 1.170951000 -0.411084000

C -1.374367000 1.775567000 -1.482442000

N -2.336254000 1.326545000 -2.313040000

H -2.440651000 1.800342000 -3.200292000

C -0.699927000 3.076037000 -1.802176000

H -0.471014000 3.150655000 -2.869182000

H -1.366492000 3.903447000 -1.524437000

H 0.227777000 3.147649000 -1.226470000

Si -3.009278000 -0.326268000 -2.030564000

H -3.905277000 -0.511681000 -3.190136000

H -1.888670000 -1.277215000 -2.020803000

C -4.050328000 -0.458703000 -0.468325000

C -5.446491000 -0.454544000 -0.563795000

C -3.469224000 -0.616403000 0.798463000

C -6.245761000 -0.592713000 0.567527000

C -4.265867000 -0.761755000 1.929384000

C -5.654032000 -0.747572000 1.816117000

H -5.920725000 -0.346388000 -1.539892000

H -2.383251000 -0.615610000 0.900761000

H -7.329552000 -0.584677000 0.473095000

H -3.800204000 -0.890516000 2.904924000

H -6.274549000 -0.861872000 2.702555000

O 0.088186000 0.617591000 2.040565000

C -0.376357000 1.718752000 2.366758000

N 0.246668000 2.873092000 2.091241000

H 1.057838000 2.853882000 1.464599000

H -0.195676000 3.751681000 2.305532000

C -1.695690000 1.795278000 3.075845000

H -2.476806000 1.581155000 2.334631000

H -1.739075000 1.010527000 3.835411000

H -1.894625000 2.767622000 3.536078000

O 2.805332000 -1.073973000 1.468991000

C 3.945728000 -1.426229000 1.131346000

N 4.151973000 -2.401548000 0.233752000

H 3.338258000 -2.710553000 -0.314755000

H 5.081700000 -2.580828000 -0.109763000

C 5.148096000 -0.743601000 1.709730000

H 5.224735000 0.225285000 1.201260000

H 4.980131000 -0.553131000 2.772039000

H 6.079650000 -1.300702000 1.571562000

Species 3

LaSiC16N5H28O5

La -0.464704000 0.150681000 0.388607000

O -0.986507000 2.572320000 1.155777000

C -0.038300000 2.645964000 2.016911000

N 0.801395000 1.644046000 2.114106000

H 1.517217000 1.763918000 2.820836000

O -0.431705000 -1.803839000 2.057862000

C 0.760954000 -2.201016000 1.758628000

N 1.425862000 -1.574635000 0.826463000

H 2.367000000 -1.914718000 0.663393000

C 0.036729000 3.892531000 2.857075000

H 0.118723000 4.763948000 2.199203000

H -0.895601000 4.003516000 3.420807000

H 0.879154000 3.885865000 3.554894000

C 1.284163000 -3.393334000 2.513808000

H 2.316666000 -3.642342000 2.251868000

H 1.214959000 -3.202093000 3.589422000

H 0.644361000 -4.258071000 2.299671000

O -1.628652000 1.096491000 -1.738828000

C -0.557016000 1.121788000 -2.414936000

N 0.596428000 0.782955000 -1.847169000

C -0.633874000 1.529781000 -3.866689000

H -0.306750000 0.701760000 -4.508284000

H -1.655877000 1.806351000 -4.134692000

H 0.034624000 2.377095000 -4.062858000

Si 2.165064000 0.707463000 -2.624648000

H 2.255368000 -0.331338000 -3.683066000

H 2.517823000 2.009582000 -3.248294000

C 3.426319000 0.263256000 -1.294917000

C 3.434270000 0.940018000 -0.069905000

C 4.345651000 -0.773236000 -1.483774000

C 4.331188000 0.593120000 0.934617000

C 5.249423000 -1.123868000 -0.483672000

C 5.242358000 -0.439078000 0.728370000

H 2.710873000 1.734746000 0.120682000

H 4.350541000 -1.323492000 -2.425384000

H 4.315640000 1.125851000 1.883744000

H 5.957845000 -1.933406000 -0.648666000

H 5.945754000 -0.711739000 1.512653000

O -2.959342000 0.153365000 0.923266000

C -3.711811000 0.914712000 0.288573000

C -4.673004000 0.353469000 -0.712689000

H -4.084019000 0.172324000 -1.621429000

H -5.060767000 -0.601344000 -0.349060000

H -5.500448000 1.028114000 -0.951334000

N -3.671720000 2.243449000 0.447545000

H -4.156347000 2.824883000 -0.218726000

H -2.823707000 2.622486000 0.885784000

O -1.513503000 -1.882095000 -0.889805000

C -2.202492000 -2.864747000 -0.584721000

C -3.042720000 -3.537457000 -1.635003000

H -3.775175000 -2.815851000 -2.010438000

H -2.399051000 -3.807056000 -2.476947000

H -3.565695000 -4.429357000 -1.278187000

N -2.234126000 -3.370906000 0.654174000

H -2.842513000 -4.139928000 0.880810000

H -1.681607000 -2.898683000 1.385986000

Transition State 3-4

LaSiC16N5H28O5

La -1.520935000 0.511870000 0.314893000

O -0.387260000 2.311508000 -1.012755000

C -0.283805000 3.149297000 -0.034461000

N -0.973109000 2.925634000 1.055228000

H -0.809639000 3.602009000 1.791291000

O -3.637066000 0.260578000 -1.151390000

C -4.447808000 0.640104000 -0.227904000

N -3.979026000 0.904685000 0.968221000

H -4.678060000 1.229887000 1.624999000

C 0.653179000 4.305513000 -0.224847000

H 1.672052000 3.911132000 -0.322208000

H 0.409327000 4.823864000 -1.157599000

H 0.626764000 5.013856000 0.607818000

C -5.900913000 0.757942000 -0.598351000

H -6.524920000 1.107484000 0.229145000

H -6.001533000 1.444418000 -1.445009000

H -6.266103000 -0.219222000 -0.932981000

O 2.284436000 -1.368521000 1.951761000

C 0.965033000 -1.438770000 1.953718000

N 0.376700000 -0.332472000 1.649829000

C 0.357211000 -2.750744000 2.320389000

H -0.713841000 -2.757327000 2.106585000

H 0.859007000 -3.558139000 1.773821000

H 0.525423000 -2.940058000 3.386542000

Si 2.341856000 0.405493000 1.590076000

H 1.862421000 1.663890000 0.894154000

H 2.697495000 0.873796000 2.947019000

C 3.891743000 0.184092000 0.450462000

C 4.263630000 1.225739000 -0.409571000

C 4.668072000 -0.982052000 0.415529000

C 5.342454000 1.110853000 -1.284177000

C 5.751678000 -1.108472000 -0.450386000

C 6.087195000 -0.065463000 -1.308956000

H 3.679537000 2.149968000 -0.398901000

H 4.400324000 -1.809672000 1.069258000

H 5.604935000 1.936920000 -1.943060000

H 6.335524000 -2.027334000 -0.459955000

H 6.927695000 -0.166448000 -1.992701000

O -0.250249000 -0.782122000 -1.502260000

C 0.972914000 -0.659934000 -1.728606000

C 1.874890000 -1.855906000 -1.656933000

H 2.010930000 -2.112489000 -0.596081000

H 1.395688000 -2.702104000 -2.157513000

H 2.865293000 -1.676752000 -2.086560000

N 1.529317000 0.517988000 -2.011577000

H 2.538793000 0.602674000 -2.007577000

H 0.960479000 1.361444000 -1.864218000

O -2.241761000 -1.989986000 0.428650000

C -2.358234000 -2.743537000 -0.552665000

C -1.583008000 -4.026039000 -0.606460000

H -0.527828000 -3.759232000 -0.744393000

H -1.668116000 -4.541735000 0.353804000

H -1.892508000 -4.695738000 -1.413683000

N -3.150059000 -2.445316000 -1.587956000

H -3.138439000 -3.015895000 -2.417881000

H -3.540308000 -1.493738000 -1.623013000

Species 4

LaSiC16N5H28O5

La 0.677831000 -0.221200000 -0.402052000

O 1.710480000 -2.108204000 1.059055000

C 0.875060000 -3.009041000 0.681895000

N 0.034771000 -2.741997000 -0.289198000

H -0.595511000 -3.505912000 -0.503621000

O 0.417633000 1.943480000 -1.799867000

C -0.697024000 1.551271000 -2.305594000

N -0.991558000 0.273784000 -2.276966000

H -1.920776000 0.051442000 -2.619653000

C 0.893930000 -4.316020000 1.428340000

H 1.912880000 -4.716146000 1.444095000

H 0.606144000 -4.129004000 2.468952000

H 0.218395000 -5.062192000 0.999525000

C -1.614860000 2.603552000 -2.867002000

H -2.498670000 2.174551000 -3.349162000

H -1.941336000 3.263027000 -2.054604000

H -1.065312000 3.219405000 -3.586085000

O -2.344838000 0.526448000 2.897456000

C -1.246450000 1.163210000 2.338883000

N -0.563892000 0.654698000 1.422698000

C -1.070698000 2.500122000 3.009375000

H -0.271048000 3.064911000 2.521615000

H -2.003798000 3.073911000 2.966894000

H -0.831356000 2.365999000 4.071185000

Si -2.734483000 -0.950085000 2.135261000

H -1.675338000 -1.976540000 2.116329000

H -3.841557000 -1.421306000 2.992317000

C -3.389377000 -0.662807000 0.386997000

C -3.902930000 0.581916000 0.006272000

C -3.416308000 -1.702422000 -0.547261000

C -4.437647000 0.778448000 -1.262662000

C -3.955447000 -1.516159000 -1.817652000

C -4.469522000 -0.272997000 -2.176845000

H -3.879731000 1.410283000 0.715035000

H -3.004438000 -2.676766000 -0.280235000

H -4.834956000 1.753160000 -1.540930000

H -3.971576000 -2.339485000 -2.528894000

H -4.896833000 -0.123760000 -3.166792000

O 2.517886000 1.345991000 0.531373000

C 2.255651000 2.468454000 0.995531000

N 1.561819000 3.382264000 0.307408000

H 1.298537000 4.251226000 0.743836000

C 2.711076000 2.827901000 2.378651000

H 2.585548000 3.886535000 2.624249000

H 2.122908000 2.224768000 3.080500000

H 3.758746000 2.541336000 2.499685000

H 1.107357000 3.096096000 -0.571670000

O 2.900559000 -0.781230000 -1.560148000

C 4.032811000 -0.879422000 -1.058913000

N 4.260783000 -1.601049000 0.043198000

H 5.168156000 -1.592809000 0.479645000

C 5.190357000 -0.160423000 -1.684198000

H 6.163540000 -0.467112000 -1.290536000

H 5.163371000 -0.313028000 -2.765816000

H 5.048566000 0.910537000 -1.501508000

H 3.445536000 -1.972860000 0.551128000

Transition State 4-5

LaSiC16N5H28O5

La -0.528815000 -0.173757000 -0.273217000

O -1.123799000 -2.507615000 -1.213254000

C -0.023417000 -2.688826000 -1.844709000

N 0.936725000 -1.806245000 -1.703091000

H 1.766028000 -2.003173000 -2.251290000

O -0.293225000 1.587261000 -2.131279000

C 0.930533000 1.875226000 -1.834228000

N 1.406106000 1.516455000 -0.671579000

H 2.392410000 1.719091000 -0.542582000

C 0.080402000 -3.913217000 -2.714207000

H -0.114112000 -4.802639000 -2.105949000

H -0.698214000 -3.874608000 -3.483333000

H 1.057200000 -4.012009000 -3.196881000

C 1.739202000 2.592327000 -2.881837000

H 2.778571000 2.747816000 -2.575138000

H 1.716430000 2.012815000 -3.810426000

H 1.278258000 3.562011000 -3.099819000

N -1.800042000 -1.053088000 1.819401000

C -0.848518000 -1.149246000 2.560171000

O 0.620048000 -0.762023000 1.892267000

C -0.562157000 -1.537663000 3.965868000

H -0.107000000 -0.708108000 4.521405000

H -1.499649000 -1.812131000 4.455821000

H 0.124239000 -2.391056000 4.007533000

Si 2.119639000 -0.557896000 2.615095000

H 2.118786000 0.555334000 3.591798000

H 2.499169000 -1.810185000 3.308043000

C 3.397711000 -0.151797000 1.293984000

C 3.592270000 -1.010753000 0.206268000

C 4.126458000 1.041186000 1.337770000

C 4.478689000 -0.682624000 -0.813758000

C 5.020385000 1.373380000 0.322352000

C 5.193236000 0.511358000 -0.757610000

H 3.015975000 -1.934137000 0.136017000

H 3.982669000 1.729655000 2.171117000

H 4.613499000 -1.357784000 -1.656934000

H 5.579772000 2.305454000 0.371334000

H 5.887673000 0.769445000 -1.554536000

O -2.970907000 -0.023544000 -1.004753000

C -3.798478000 -0.752161000 -0.427660000

C -4.778717000 -0.154810000 0.532555000

H -4.213760000 0.017672000 1.458690000

H -5.125891000 0.807061000 0.146530000

H -5.633439000 -0.802676000 0.747738000

N -3.828814000 -2.077652000 -0.619436000

H -4.369771000 -2.643409000 0.016310000

H -2.986250000 -2.494575000 -1.029388000

O -1.541234000 1.949959000 0.851476000

C -2.152629000 2.936139000 0.418707000

C -3.024066000 3.734517000 1.347926000

H -3.808956000 3.078064000 1.736286000

H -2.422000000 4.053513000 2.203307000

H -3.483334000 4.610375000 0.880723000

N -2.073681000 3.335166000 -0.855787000

H -2.618291000 4.114026000 -1.186787000

H -1.497026000 2.785904000 -1.509295000

Species 5

LaSiC14N4H25O5

La -0.975943000 0.441607000 -0.160769000

O 0.624594000 -0.802111000 1.430689000

C 0.742872000 -1.846052000 0.694841000

N -0.050313000 -1.984459000 -0.343292000

H 0.137848000 -2.817580000 -0.888629000

O -2.847565000 -0.310122000 -1.749925000

C -3.572626000 0.745883000 -1.602403000

N -3.097448000 1.760922000 -0.926426000

H -3.738965000 2.541893000 -0.856449000

O 0.784404000 1.418821000 -1.224447000

C 1.795812000 -2.844291000 1.088417000

H 2.777059000 -2.356232000 1.055208000

H 1.620794000 -3.162224000 2.121967000

H 1.818175000 -3.721373000 0.433952000

C -4.949979000 0.709113000 -2.207789000

H -5.489287000 1.654491000 -2.098589000

H -4.873632000 0.453098000 -3.269210000

H -5.528541000 -0.087505000 -1.724649000

H 2.760072000 2.250870000 -2.658297000

Si 2.387187000 1.715697000 -1.326410000

H 2.873574000 2.684870000 -0.297664000

C 3.425000000 0.154404000 -1.022707000

C 4.494230000 0.153380000 -0.122226000

C 3.153986000 -1.031186000 -1.718275000

C 5.281122000 -0.980420000 0.069883000

C 3.935417000 -2.167734000 -1.536740000

C 5.003895000 -2.142780000 -0.643032000

H 4.727172000 1.064752000 0.432379000

H 2.315270000 -1.063960000 -2.415299000

H 6.112695000 -0.956935000 0.771768000

H 3.712726000 -3.077706000 -2.091625000

H 5.617973000 -3.029814000 -0.500944000

O -0.453740000 2.131018000 1.663326000

C 0.692494000 2.494715000 1.986106000

N 1.673295000 1.625551000 2.246469000

H 1.505753000 0.621894000 2.090503000

H 2.610435000 1.956536000 2.411840000

C 1.011262000 3.957043000 2.070180000

H 1.013977000 4.356169000 1.049530000

H 0.215030000 4.464938000 2.620059000

H 1.978340000 4.168235000 2.534886000

O -2.651859000 -0.841699000 1.371154000

C -3.115479000 -1.976582000 1.188233000

N -3.620908000 -2.361767000 0.007753000

H -3.899661000 -3.318562000 -0.137260000

C -3.114527000 -2.975036000 2.308844000

H -3.712851000 -3.867720000 2.105345000

H -2.075365000 -3.272650000 2.489158000

H -3.473731000 -2.488334000 3.218825000

H -3.476670000 -1.740241000 -0.801152000

Species 6

LaSiC16N5H30O6

La -0.883853000 0.336118000 0.234284000

O 0.682822000 -1.634564000 -0.571766000

C 1.011719000 -1.054595000 -1.664985000

N 0.353077000 0.020376000 -2.032497000

H 0.707455000 0.451565000 -2.878313000

O -2.929228000 1.251371000 -1.168240000

C -3.690625000 1.333031000 -0.143560000

N -3.177999000 1.228068000 1.062152000

H -3.869362000 1.273248000 1.801470000

O 1.016351000 0.812698000 1.574121000

C 2.140722000 -1.660246000 -2.453595000

H 3.032182000 -1.703683000 -1.816618000

H 1.882048000 -2.691353000 -2.722435000

H 2.376013000 -1.098324000 -3.362676000

C -5.166546000 1.505341000 -0.402508000

H -5.751964000 1.638377000 0.512408000

H -5.321803000 2.365152000 -1.061965000

H -5.535593000 0.619573000 -0.933920000

H 3.003251000 0.454123000 3.170132000

Si 2.321143000 -0.096948000 1.966828000

H 2.029118000 -1.526623000 2.252554000

C 3.585031000 -0.085009000 0.548207000

C 4.569440000 -1.072441000 0.443591000

C 3.558500000 0.922732000 -0.421573000

C 5.507841000 -1.052120000 -0.584663000

C 4.490617000 0.949753000 -1.456437000

C 5.469431000 -0.036764000 -1.537279000

H 4.601884000 -1.879260000 1.178386000

H 2.784461000 1.691161000 -0.367440000

H 6.267387000 -1.829354000 -0.647620000

H 4.454292000 1.741304000 -2.203203000

H 6.199599000 -0.017662000 -2.344050000

O -1.151891000 -1.428885000 2.087497000

C -0.931880000 -2.636852000 2.251787000

N -0.191472000 -3.364202000 1.408253000

H 0.238951000 -2.906670000 0.595498000

H 0.001206000 -4.332058000 1.604383000

C -1.507642000 -3.335908000 3.451325000

H -1.141896000 -2.837392000 4.353700000

H -2.594683000 -3.216204000 3.434262000

H -1.261630000 -4.400429000 3.502092000

O -2.331307000 -1.673291000 -0.646603000

C -2.444025000 -1.917480000 -1.856600000

N -3.026224000 -1.057697000 -2.704405000

H -2.973064000 -1.215374000 -3.697816000

C -1.920747000 -3.216981000 -2.396522000

H -2.049605000 -3.339939000 -3.475682000

H -0.856184000 -3.262179000 -2.136974000

H -2.413975000 -4.042067000 -1.874037000

H -3.185922000 -0.104412000 -2.342257000

O -0.409143000 2.847031000 -0.208797000

C 0.245824000 3.748338000 0.339358000

N 1.097476000 3.504145000 1.337813000

H 1.610997000 4.250979000 1.775504000

C 0.081388000 5.167700000 -0.127256000

H 0.722860000 5.880287000 0.399222000

H 0.293797000 5.210474000 -1.199341000

H -0.965516000 5.457290000 0.002743000

H 1.200164000 2.507331000 1.627593000

Transition State 6-7

LaSi2C22N5H38O6

La -0.450305000 -0.930772000 0.226904000

O -1.290543000 -1.033533000 -2.299710000

C -2.305850000 -0.280536000 -2.096536000

N -2.581944000 0.098966000 -0.871969000

H -3.391162000 0.704542000 -0.793042000

O -2.371631000 -1.824949000 1.896150000

C -1.521442000 -2.480931000 2.616051000

N -0.246824000 -2.245107000 2.472757000

H 0.347193000 -2.821944000 3.057444000

O 0.793922000 1.039689000 -0.690162000

C -3.116666000 0.125744000 -3.299674000

H -2.481549000 0.712591000 -3.974017000

H -3.426783000 -0.769914000 -3.849752000

H -4.001600000 0.715570000 -3.040598000

C -2.085406000 -3.526537000 3.540124000

H -1.327369000 -3.980021000 4.185073000

H -2.875326000 -3.089201000 4.159332000

H -2.548873000 -4.313528000 2.932270000

H 1.532062000 2.721081000 -2.523746000

Si 0.354709000 2.208867000 -1.791642000

H -0.605076000 1.631322000 -2.759389000

C -0.520923000 3.662314000 -0.960919000

C -1.898011000 3.852645000 -1.117788000

C 0.186235000 4.573088000 -0.165936000

C -2.550154000 4.928161000 -0.519118000

C -0.457910000 5.647337000 0.439740000

C -1.827130000 5.828790000 0.259176000

H -2.466555000 3.148652000 -1.728677000

H 1.260163000 4.440493000 -0.024410000

H -3.619890000 5.069149000 -0.663296000

H 0.107996000 6.348355000 1.049861000

H -2.331054000 6.673486000 0.724867000

O 1.562380000 -1.898332000 -1.014758000

C 2.122451000 -1.555943000 -2.068467000

N 1.424978000 -1.163383000 -3.144464000

H 0.404930000 -1.082114000 -3.073893000

H 1.901105000 -0.839307000 -3.970548000

C 3.617196000 -1.543666000 -2.166374000

H 3.989131000 -0.638286000 -1.666328000

H 4.020428000 -2.393668000 -1.610797000

H 3.981601000 -1.558570000 -3.197786000

O -1.172588000 -3.355422000 -0.451653000

C -2.290885000 -3.598038000 -0.926528000

N -3.416321000 -3.364854000 -0.230813000

H -4.319039000 -3.465656000 -0.665498000

C -2.423145000 -4.132119000 -2.320413000

H -3.426843000 -4.493092000 -2.563879000

H -2.148846000 -3.306440000 -2.989206000

H -1.693697000 -4.931943000 -2.469087000

H -3.316711000 -2.843182000 0.649060000

O -1.003900000 1.061483000 1.729575000

C -1.957861000 1.546091000 2.349613000

N -3.020937000 0.809454000 2.706999000

H -3.772324000 1.211216000 3.242584000

C -1.957856000 3.004588000 2.693180000

H -2.681141000 3.270448000 3.469991000

H -0.948601000 3.286794000 3.005178000

H -2.181175000 3.576507000 1.783060000

H -3.004691000 -0.193323000 2.486209000

H 1.601608000 -0.005298000 1.154397000

Si 2.276285000 1.268949000 0.533533000

H 2.792907000 2.253662000 -0.513896000

C 4.012613000 0.428589000 0.772356000

C 4.142656000 -0.805558000 1.416076000

C 5.164784000 1.000229000 0.222039000

C 5.375657000 -1.442722000 1.522103000

C 6.404916000 0.374064000 0.320424000

C 6.510501000 -0.852768000 0.970616000

H 3.245570000 -1.281919000 1.819498000

H 5.078855000 1.949939000 -0.310659000

H 5.453932000 -2.405818000 2.024495000

H 7.289322000 0.835886000 -0.116074000

H 7.475059000 -1.352641000 1.040376000

H 1.923494000 2.206834000 1.639269000

Species 7

LaSi2C22N5H38O6

La 0.641720000 -0.858241000 -0.279461000

O 1.356463000 -0.822799000 2.292435000

C 2.175223000 0.152512000 2.157104000

N 2.387116000 0.643059000 0.960714000

H 3.037630000 1.420187000 0.933406000

O 2.860214000 -1.216949000 -1.774213000

C 2.256546000 -2.026128000 -2.581973000

N 0.955343000 -2.101306000 -2.553933000

H 0.566739000 -2.775839000 -3.203003000

O -1.083771000 0.789559000 0.584510000

C 2.835024000 0.676470000 3.406102000

H 2.062128000 1.074142000 4.074882000

H 3.322121000 -0.150524000 3.935039000

H 3.570762000 1.461718000 3.205714000

C 3.131176000 -2.871467000 -3.468247000

H 2.561596000 -3.468576000 -4.186085000

H 3.841145000 -2.235303000 -4.006724000

H 3.718571000 -3.546545000 -2.833575000

H -2.256695000 2.203972000 2.434801000

Si -0.970985000 1.999683000 1.740218000

H 0.047033000 1.581199000 2.727561000

C -0.370361000 3.630241000 1.000047000

C 0.918452000 4.097738000 1.282450000

C -1.185461000 4.403653000 0.163202000

C 1.377205000 5.306476000 0.764203000

C -0.733361000 5.610802000 -0.360095000

C 0.547517000 6.066088000 -0.056639000

H 1.571856000 3.504778000 1.925485000

H -2.189445000 4.052076000 -0.078349000

H 2.378325000 5.659983000 1.004317000

H -1.381944000 6.200868000 -1.004343000

H 0.899491000 7.013525000 -0.460122000

O -1.147790000 -2.260367000 0.892215000

C -1.816469000 -2.072803000 1.920730000

N -1.282272000 -1.526049000 3.022979000

H -0.304764000 -1.215611000 2.999564000

H -1.853415000 -1.347562000 3.832760000

C -3.270850000 -2.429046000 1.959060000

H -3.832293000 -1.628956000 1.456934000

H -3.432510000 -3.340269000 1.378828000

H -3.656522000 -2.554685000 2.975167000

O 1.841852000 -3.082091000 0.415610000

C 2.939162000 -3.078391000 0.990432000

N 4.029903000 -2.536875000 0.423221000

H 4.890008000 -2.452531000 0.939965000

C 3.076378000 -3.651366000 2.368197000

H 4.113029000 -3.804007000 2.683074000

H 2.582918000 -2.940261000 3.042365000

H 2.527501000 -4.594426000 2.421891000

H 3.888041000 -2.007785000 -0.446838000

O 0.822561000 1.266033000 -1.697791000

C 1.684596000 1.979015000 -2.224206000

N 2.914990000 1.520615000 -2.501602000

H 3.592415000 2.105543000 -2.961824000

C 1.377911000 3.411741000 -2.540141000

H 2.062685000 3.851683000 -3.271592000

H 0.348079000 3.475391000 -2.901536000

H 1.428066000 3.992760000 -1.609915000

H 3.119809000 0.536075000 -2.293532000

H -1.476845000 -0.375895000 -1.317668000

Si -2.381800000 0.830855000 -0.729964000

H -3.105099000 1.886011000 0.135033000

C -3.936779000 -0.310055000 -0.861850000

C -3.887097000 -1.552852000 -1.501822000

C -5.147262000 0.073532000 -0.274394000

C -5.004855000 -2.378962000 -1.566986000

C -6.271370000 -0.747834000 -0.326027000

C -6.200022000 -1.978075000 -0.973695000

H -2.941121000 -1.876242000 -1.940640000

H -5.198763000 1.034570000 0.240699000

H -4.945422000 -3.341704000 -2.072424000

H -7.203151000 -0.431846000 0.140592000

H -7.073696000 -2.626405000 -1.012446000

H -2.120951000 1.701756000 -1.916235000

Transition State 7-8

LaSi2C22N5H38O6

La -1.156474000 -0.255492000 0.451050000

O -1.652740000 -0.107091000 -2.201880000

C -1.726306000 1.172795000 -2.146252000

N -1.117672000 1.797646000 -1.171532000

H -1.294598000 2.796325000 -1.137985000

O -3.528805000 0.796370000 0.785074000

C -3.669917000 0.348336000 1.983811000

N -2.740981000 -0.418595000 2.486838000

H -2.884460000 -0.695108000 3.450216000

O 1.476365000 0.221755000 -0.232402000

C -2.544618000 1.856013000 -3.209348000

H -2.145689000 1.594416000 -4.195724000

H -3.570198000 1.472035000 -3.164497000

H -2.562309000 2.944337000 -3.104030000

C -4.923029000 0.745988000 2.718685000

H -4.954429000 0.363312000 3.743260000

H -5.003111000 1.837719000 2.734890000

H -5.794344000 0.367517000 2.171484000

H 3.141336000 0.424213000 -2.166426000

Si 1.856043000 1.004997000 -1.693799000

H 0.828303000 0.698385000 -2.692116000

C 2.168720000 2.827874000 -1.356922000

C 1.603617000 3.470311000 -0.247263000

C 3.013781000 3.558128000 -2.198768000

C 1.873022000 4.814042000 -0.002358000

C 3.274749000 4.903783000 -1.960166000

C 2.702527000 5.532731000 -0.858840000

H 0.943068000 2.919045000 0.426953000

H 3.484230000 3.068923000 -3.053637000

H 1.428344000 5.304201000 0.862253000

H 3.931036000 5.459227000 -2.626953000

H 2.908356000 6.583537000 -0.665024000

O 0.058130000 -2.370072000 -0.456643000

C 0.471664000 -2.774940000 -1.549243000

N 0.117713000 -2.202230000 -2.711909000

H -0.513719000 -1.390334000 -2.702407000

H 0.487087000 -2.547129000 -3.582583000

C 1.433111000 -3.925613000 -1.615086000

H 2.420180000 -3.572860000 -1.287830000

H 1.116377000 -4.692041000 -0.903789000

H 1.522122000 -4.359621000 -2.615479000

O -2.921233000 -1.982710000 -0.421360000

C -3.778595000 -1.768471000 -1.288122000

N -4.676115000 -0.775653000 -1.182432000

H -5.293491000 -0.572175000 -1.951726000

C -3.826417000 -2.608621000 -2.530490000

H -4.808370000 -2.618585000 -3.013801000

H -3.092234000 -2.175625000 -3.220884000

H -3.514448000 -3.627733000 -2.292984000

H -4.507303000 -0.070278000 -0.450069000

O -0.381636000 1.716844000 1.860692000

C 0.079097000 1.736798000 3.035694000

N 0.212384000 0.639184000 3.754085000

H 0.685328000 0.737383000 4.643827000

C 0.542735000 3.069068000 3.567108000

H 0.723070000 3.068994000 4.646290000

H 1.477537000 3.332698000 3.054511000

H -0.194243000 3.837362000 3.315900000

H 0.199664000 -0.381008000 3.031283000

H 0.229677000 -1.091155000 2.244905000

Si 2.723108000 -0.105552000 0.890545000

H 3.904038000 0.636186000 0.387019000

C 3.247204000 -1.911856000 0.895646000

C 2.583967000 -2.904325000 1.629896000

C 4.379871000 -2.276118000 0.154646000

C 3.049023000 -4.215110000 1.630745000

C 4.840479000 -3.590451000 0.143105000

C 4.175516000 -4.560030000 0.887745000

H 1.688532000 -2.637680000 2.192354000

H 4.917221000 -1.516828000 -0.415851000

H 2.528210000 -4.973203000 2.212153000

H 5.722550000 -3.854728000 -0.436542000

H 4.537032000 -5.586502000 0.890372000

H 2.331366000 0.402348000 2.209845000

Species 8

LaSi2C22N5H36O6

La -1.136406000 -0.187085000 0.460786000

O -1.596510000 0.404671000 -2.087037000

C -1.625531000 1.661582000 -1.828067000

N -0.981028000 2.104055000 -0.778043000

H -1.124402000 3.090465000 -0.587618000

O -3.522595000 0.866648000 0.651461000

C -3.849478000 0.255070000 1.736043000

N -2.992892000 -0.560450000 2.288398000

H -3.324399000 -0.996561000 3.141011000

O 1.563598000 0.361639000 -0.201432000

C -2.451099000 2.532711000 -2.735273000

H -2.202253000 2.314367000 -3.779040000

H -3.509038000 2.283530000 -2.592044000

H -2.311129000 3.599864000 -2.541116000

C -5.233044000 0.515520000 2.271987000

H -5.420561000 0.019132000 3.228942000

H -5.382102000 1.593972000 2.385452000

H -5.970880000 0.162589000 1.541031000

H 3.183137000 0.900445000 -2.109315000

Si 1.900380000 1.376280000 -1.523392000

H 0.864381000 1.213867000 -2.548121000

C 2.190427000 3.122273000 -0.891532000

C 1.716112000 3.513605000 0.367082000

C 2.926639000 4.039221000 -1.648163000

C 1.965091000 4.797208000 0.844263000

C 3.166717000 5.326152000 -1.177132000

C 2.684203000 5.705060000 0.071955000

H 1.145336000 2.811537000 0.978273000

H 3.326738000 3.745324000 -2.620334000

H 1.594270000 5.089415000 1.824461000

H 3.737346000 6.030313000 -1.779233000

H 2.875267000 6.708625000 0.446791000

O 0.120875000 -2.143262000 -0.678527000

C 0.498056000 -2.392414000 -1.829911000

N 0.084968000 -1.685273000 -2.895455000

H -0.527342000 -0.869840000 -2.760727000

H 0.455017000 -1.886808000 -3.810039000

C 1.481762000 -3.496366000 -2.083332000

H 2.479420000 -3.139840000 -1.795363000

H 1.246203000 -4.342809000 -1.434247000

H 1.506866000 -3.821146000 -3.127806000

O -2.812101000 -1.859622000 -0.688836000

C -3.701756000 -1.657496000 -1.524198000

N -4.588629000 -0.657051000 -1.413433000

H -5.221904000 -0.461283000 -2.171448000

C -3.809607000 -2.549218000 -2.728632000

H -4.748719000 -2.437720000 -3.278727000

H -2.973889000 -2.301829000 -3.393455000

H -3.685008000 -3.588919000 -2.416548000

H -4.399671000 0.059690000 -0.697246000

O -0.255299000 1.207110000 2.381877000

C -0.018869000 0.199583000 3.125977000

N 0.206178000 -0.979157000 2.573972000

H 0.277186000 -1.718653000 3.266331000

C -0.067787000 0.415387000 4.615348000

H 0.304444000 -0.440772000 5.186703000

H 0.512998000 1.307085000 4.871110000

H -1.107841000 0.613387000 4.900210000

Si 2.841183000 -0.075456000 0.844717000

H 4.019475000 0.684395000 0.364113000

C 3.327494000 -1.883447000 0.645055000

C 2.635742000 -2.942029000 1.247580000

C 4.455244000 -2.185293000 -0.130345000

C 3.066295000 -4.255076000 1.092074000

C 4.884581000 -3.499962000 -0.297913000

C 4.190846000 -4.535967000 0.319646000

H 1.744589000 -2.724674000 1.832674000

H 5.012846000 -1.376340000 -0.604879000

H 2.521475000 -5.065000000 1.573175000

H 5.764691000 -3.713890000 -0.900863000

H 4.527532000 -5.563859000 0.200020000

H 2.527020000 0.305309000 2.221332000

References

[1] J. O. Rothbaum, A. Motta, Y. Kratish, T. J. Marks, *J. Am. Chem. Soc.* **2022**, *144*, 17086-17096.

[2] R. Ray, S. Chandra, V. Yadav, P. Mondal, D. Maiti, G. K. Lahiri, *Chem. Commun.* **2017**, *53*, 4006-4009.

[3] D. J. Quinn, G. J. Haun, G. Moura-Letts, *Tetrahedron Lett.* **2016**, *57*, 3844-3847.

[4] M. M. Guru, T. Shima, Z. Hou, *Angew. Chem. Int. Ed.* **2016**, *55*, 12316-12320.

[5] A. Nagendiran, H. Sörensen, M. J. Johansson, C.-W. Tai, J.-E. Bäckvall, *Green Chem.* **2016**, *18*, 2632-2637.

[6] S. Nandi, P. Patel, A. Jakhar, N. H. Khan, A. V. Biradar, R. I. Kureshy, H. C. Bajaj, *ChemistrySelect* **2017**, *2*, 9911-9919.

[7] S. A. Shipilovskikh, V. Y. Vaganov, E. I. Denisova, A. E. Rubtsov, A. V. Malkov, *Org. Lett.* **2018**, *20*, 728-731.

[8] S. M. Ghodse, B. S. Takale, N. T. Hatvate, V. N. Telvekar, *ChemistrySelect* **2018**, *3*, 4168-4172.

[9] J. Xiao, Q. Li, T. Chen, L.-B. Han, *Tetrahedron Lett.* **2015**, *56*, 5937-5940.

[10] J. Jouha, M. Khouili, M.-A. Hiebel, G. Guillaumet, F. Suzenet, *Tetrahedron Lett.* **2018**, *59*, 3108-3111.

[11] C. Zhu, F. Chen, C. Liu, H. Zeng, Z. Yang, W. Wu, H. Jiang, *J. Org. Chem.* **2018**, *83*, 14713-14722.

[12] A. Chatupheeraphat, H.-H. Liao, S.-C. Lee, M. Rueping, *Org. Lett.* **2017**, *19*, 4255-4258.

[13] K. Hyodo, K. Togashi, N. Oishi, G. Hasegawa, K. Uchida, *Org. Lett.* **2017**, *19*, 3005-3008.

[14] R. Takise, K. Itami, J. Yamaguchi, *Org. Lett.* **2016**, *18*, 4428-4431.

[15] S. Chakraborty, A. Biswas, S. K. Mandal, *ChemCatChem* **2023**, *15*, e202300462.

[16] A. Yousri, M. Haukka, M. A. M. Abu-Youssef, M. Salah Ayoup, M. M. F. Ismail, N. G. El Menofy, S. M. Soliman, A. Barakat, F. M. Amombo Noa, L. Öhrström, *CrystEngComm* **2023**, *25*, 3922-3930.

[17] K. Zheng, C. Liang, H. Chen, Y. Zhao, Z. Wang, J. Cheng, *Org. Lett.* **2024**, *26*, 3935-3939.

[18] R. Wamser, S. Pach, C. Arkona, M. Baumgardt, U. B. A. Aziz, A. C. Hocke, G. Wolber, J. Rademann, *ChemMedChem* **2023**, *18*, e202200635.

[19] U. Dasmahapatra, B. Maiti, K. Chanda, *Org. Biomol. Chem.* **2024**, *22*, 8459-8471.

[20] R. Y. Liu, M. Bae, S. L. Buchwald, *J. Am. Chem. Soc.* **2018**, *140*, 1627-1631.

[21] M. Kurfürst, V. Blechta, J. Schraml, *Magn. Reson. Chem.* **2011**, *49*, 492-501.

[22] X. Wu, G. Ding, W. Lu, L. Yang, J. Wang, Y. Zhang, X. Xie, Z. Zhang, *Org. Lett.* **2021**, *23*, 1434-1439.

[23] J. Zhang, Y. Wang, X. Zhou, *Chem. Commun.* **2023**, *59*, 3253-3256.

[24] Y. Zhao, D. G. Truhlar, *Theor. Chem. Acc.* **2008**, *120*, 215-241.

[25] P. J. Hay, W. R. Wadt, *J. Chem. Phys.* **1985**, *82*, 299-310.

[26] W. J. Hehre, R. Ditchfield, J. A. Pople, *J. Chem. Phys.* **1972**, *56*, 2257-2261.

[27] M. J. Frisch, G. W. Trucks, H. B. Schlegel, G. E. Scuseria, M. A. Robb, J. R. Cheeseman, G. Scalmani, V. Barone, G. A. Petersson, H. Nakatsuji, X. Li, M. Caricato, A. V. Marenich, J. Bloino, B. G. Janesko, R. Gomperts, B. Mennucci, H. P. Hratchian, J. V. Ortiz, A. F. Izmaylov, J. L. Sonnenberg, Williams, F. Ding, F. Lipparini, F. Egidi, J. Goings, B. Peng, A. Petrone, T. Henderson, D. Ranasinghe, V. G. Zakrzewski, J. Gao, N. Rega, G. Zheng, W. Liang, M. Hada, M. Ehara, K. Toyota, R. Fukuda, J. Hasegawa, M. Ishida, T. Nakajima, Y. Honda, O. Kitao, H. Nakai, T. Vreven, K. Throssell, J. A. Montgomery Jr., J. E. Peralta, F. Ogliaro, M. J. Bearpark, J. J. Heyd, E. N. Brothers, K. N. Kudin, V. N. Staroverov, T. A. Keith, R. Kobayashi, J. Normand, K. Raghavachari, A. P. Rendell, J. C. Burant, S. S. Iyengar, J. Tomasi, M. Cossi, J. M. Millam, M. Klene, C. Adamo, R. Cammi, J. W. Ochterski, R. L. Martin, K. Morokuma, O. Farkas, J. B. Foresman, D. J. Fox, Gaussian 16 Rev. C.01, Wallingford, CT, **2016**.

[28] H. S. Rzepa, KINISOT. A Basic Program to Calculate Kinetic Isotope Effects Using Normal Coordinate Analysis of Transition State and Reactants*,* Zenodo, **2015**.
